# Synthesis of CHF_2_-Containing Heterocycles through Oxy-difluoromethylation Using Low-Cost 3D Printed PhotoFlow Reactors

**DOI:** 10.1021/acs.orglett.3c03997

**Published:** 2024-01-08

**Authors:** Jinlei Zhang, Elias Selmi-Higashi, Shen Zhang, Alexandre Jean, Stephen T. Hilton, Xacobe C. Cambeiro, Stellios Arseniyadis

**Affiliations:** † Department of Chemistry, Queen Mary University of London, Mile End Road, London E1 4NS, United Kingdom; ‡ School of Science, University of Greenwich, Central Avenue, Gillingham ME4 4TB, United Kingdom; § Industrial Research Centre, Oril Industrie, 13 rue Desgenétais, Bolbec 76210, France; ∥ UCL School of Pharmacy, University College London, 29-39 Brunswick Square, London WC1N 1AX, United Kingdom

## Abstract

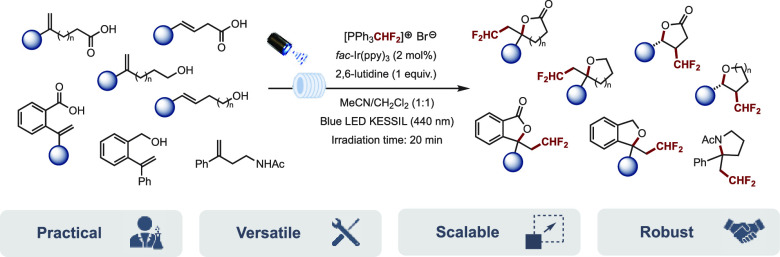

We report here a highly straightforward access to a variety of CHF_2_-containing heterocycles, including lactones, tetrahydrofurans, tetrahydropyrans, benzolactones, phthalanes, and pyrrolidines, through a visible light-mediated intramolecular oxy-difluoromethylation under continuous flow. The method, which relies on the use of readily available starting materials, low-cost 3D printed photoflow reactors, and difluoromethyltriphenylphosphonium bromide used here as a CHF_2_ radical precursor, is practical and scalable and provides the desired products in moderate to excellent yields and excellent regio- and stereoselectivities.

The addition of fluorine-containing groups can dramatically alter the properties of bioactive molecules, enhancing their lipophilicity and often improving their metabolic stability, their pharmacokinetic properties, and their bioavailability.^1−4^ For all of these reasons, tremendous efforts have been dedicated over the past few decades to the development of efficient synthetic methods enabling the incorporation of these groups, with a special emphasis given to late-stage functionalization strategies,^5^ which remains an area ripe for exploration. While a large body of work has been focused on the development of effective fluorination^6^ and trifluoromethylation reactions,^7^ the synthetic community has recently turned their attention to the difluoromethyl group as it has emerged as a promising bioisosteric substitute for hydroxyls, thiols, amines and hydroxamic acids due to its ability to act as a weak hydrogen bond donor.^8^

As heterocycles are ubiquitous in medicinal chemistry, their synthesis and functionalization have always been an area of intense scrutiny.^9^ Several groups around the world have tackled the challenging task of developing methods that provide a direct access to (per)fluoroalkylated heterocycles, particularly lactones, starting from linear precursors, but the number of effective methods are limited (Figure 1A and B).^10^ Over the years, our group has been interested in developing new synthetic methods to access a variety of diversely functionalized heterocyclic scaffolds,^11^ including one that allows access to a variety of tertiary difluoromethylated lactones, lactams, glutaramides, succinimides, and quinolinones via a sequential sulfoximine-mediated difluoromethylation/palladium-catalyzed decarboxylative protonation.^12^ Surprisingly, despite the number of methods reporting the fluorination and fluoroalkylation of alkenes/alkynes to construct fluoro-containing heterocyclic scaffolds,^13^ methods affording CHF_2_-substituted heterocycles are rather scarce. One such example was recently reported by Xu and co-workers featuring an electrochemical oxy-difluoromethylation of alkenes to form the corresponding lactones, albeit in only moderate yields (Figure 1C).^14^

**Figure 1:**
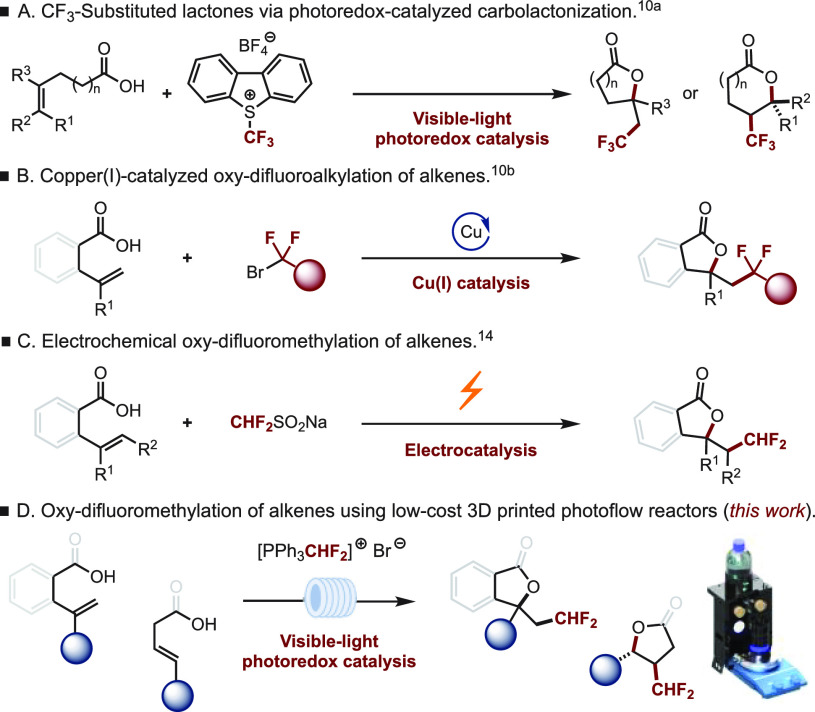
Strategies for the oxidifluoroalkylation of alkenes.

Following our recent work on the synthesis of α-CHF_2_ substituted ketones through the difluoromethylation of enol silanes under photoredox conditions,^15^ we set out to develop a new, practical, and scalable method to access a variety of CHF_2_-substituted heterocycles via a photocatalytic oxy-difluoromethylation of functionalized alkenes under continuous flow conditions (Figure 1D). Indeed, flow chemistry has emerged as a powerful tool,^16^ particularly for conducting photoredox processes.^17^ In contrast to batch reactions, flow chemistry offers substantial advantages, in particular, a larger surface area-to-volume ratio and provides a better light penetration within the reaction media and a swift mixing of the reagents, resulting in a higher efficiency. Additionally, the use of microreactors in flow chemistry provides a higher degree of control over the reaction parameters and a more straightforward scale-up of the reactions. Despite the many benefits of continuous flow chemistry, its widespread adoption by synthetic chemists has been limited by the substantial costs associated with its implementation. The recent development of low-cost 3D printed reactors has provided researchers with new opportunities to leverage the benefits of flow chemistry at a more affordable expense.^18^ Most importantly, the application of 3D printing technology in flow chemistry has enabled the creation of bespoke flow reactors that are tailored to specific reaction requirements. Hence, Hilton and co-workers reported the development of a modular, small-footprint, and low-cost 3D printed continuous-flow system and demonstrated its use in flow photochemistry.^19^ This innovative system allows for easy integration with existing stirrer hot plates, and its flow is driven and controlled by compressed air. The 3D printed circular disk reactor (CDR) has a path length that can be extended and connected to create various flow path volumes, while the residence time can be easily controlled by using resistive capillaries. The system is also associated with a 3D printed adaptor for a Kessil lamp specially designed for flow photochemistry.

We initiated our study by conducting the first set of reactions in batch using **1a** as a model substrate. We evaluated three different difluoromethylating reagents (**dFM**_**1**_–**dFM**_**3**_) based on their inherent solubility and oxido-reduction potentials as well as several photocatalysts (Table 1). As a general trend, the best result was obtained when running the reaction in DCM [0.1 M] at rt overnight under light irradiation using a 440 nm Kessil lamp, and using difluoromethyltriphenylphosphonium bromide (**dFM**_**2**_, 1.2 equiv.)^21^ in conjunction with *fac*-Ir(ppy)_3_ (2 mol %) and 1 equiv. of 2,6-lutidine (81%, Table 1, entry 2). In comparison, the use of Hu’s reagent (**dFM**_**1**_)^20^ under otherwise identical conditions only led to 52% yield (Table 1, entry 1). Unfortunately, neither 4CzlPN nor perylene, two widely used organic photocatalysts, were compatible with the phosphonium salt as no product was formed (Table 1, entries 3 and 4). Interestingly, the use of 10-phenylphenothiazine and perylene in conjunction with the sulfonium salt **C**(22) afforded the desired product, albeit in only 8 and 52% yield, respectively (Table 1, entries 5 and 6).

**Table 1:**
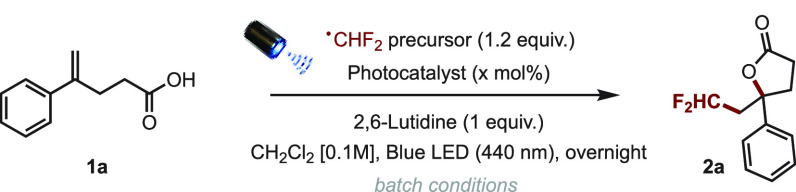

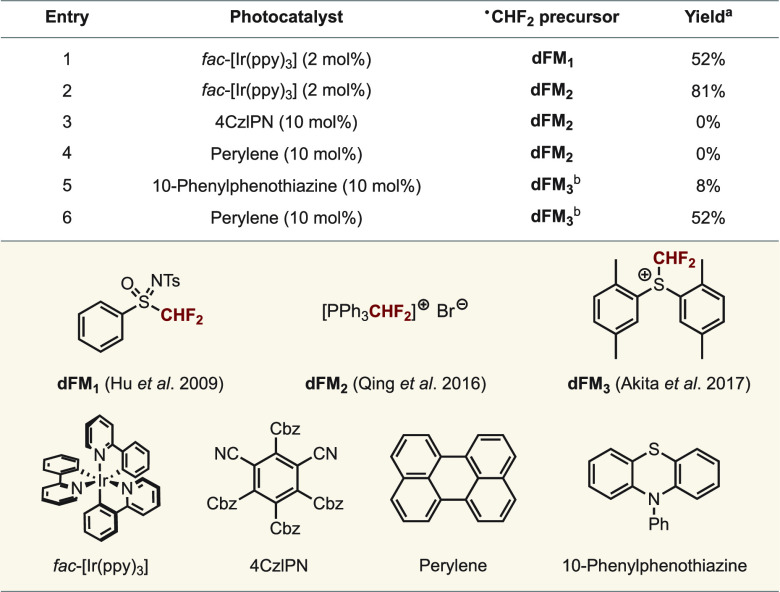
Systematic Study under Batch Conditions

a Determined by ^19^F NMR using trifluorotoluene as an internal standard.

b Using **1** equiv. of dFM_**3**_.

After identifying the most favorable conditions in batch, we sought to implement this protocol into our 3D printed photoflow system. We first conducted a screening of various bases and solvents. Given the limited compatibility of the polypropylene CDR with certain organic solvents, we tested DMF and MeCN. Interestingly, the reactions run with 1 equiv. of **dFM**_**2**_ and 1 equiv. of 2,6-lutidine in both solvents led to the desired lactone in 50 and 71% yield, respectively (see the Supporting Information for more details), while the reaction run with 2,6-di-*tert*-butylpyridine instead of 2,6-lutidine brought the yield back down to 50%. Most importantly, the use of the 3D printed photoflow system significantly reduced the reaction time from several hours to only 20 min. However, although acetonitrile showed promise, the limited solubility of the reagents raised some concerns about potential flow blockages. To circumvent this issue, we first attempted to lower the concentration from 0.1 to 0.05 M, but this had a detrimental effect on the yield. We then decided to run the reaction in a 1:1 MeCN/DCM mixture. This sounded counterintuitive at first as the use of neat DCM is in theory incompatible with the polypropylene reactor, causing material softening or swelling over time; however, the mixed solvent conditions proved perfectly well suited as no noticeable change of the photoreactor was observed even after several cycles of utilization.

After establishing the optimized reaction conditions [**dFM**_**2**_ (2 equiv.), *fac*-Ir(ppy)_3_ (2 mol %), 2,6-lutidine (1 equiv.), CH_3_CN/CH_2_Cl_2_ (1:1), rt, 8 W Blue LED (440 nm), flow rate: 100 μL/min, residence time = 20 min)], we proceeded to examine the substrate scope starting with terminal alkenes **1b**–**i** (Figure 2). The reaction appeared to be tolerant of substrates bearing both electron-donating and electron-withdrawing groups on the aromatic ring. Hence, the *para-*methyl (**2b**, 75%), *para*-fluoro (**2c**, 70%), *para*-chloro (**2d**, 75%), and *para*-bromo (**2e**, 72%) derivatives were all obtained in high yields. The method was also successfully applied to the bicyclic precursor **1f** and ene-yne **1g** to form the corresponding difluoromethyl-containing spirolactone **2f** and the phenyl acetylene-containing butyrolactone **2g** in 69 and 38% yield, respectively. Finally, increasing the length of the alkyl chain to generate the corresponding 6- and 7-membered lactones **2h** (45%) and **2i** (11%) also proved feasible although the yields were more moderate.

**Figure 2:**
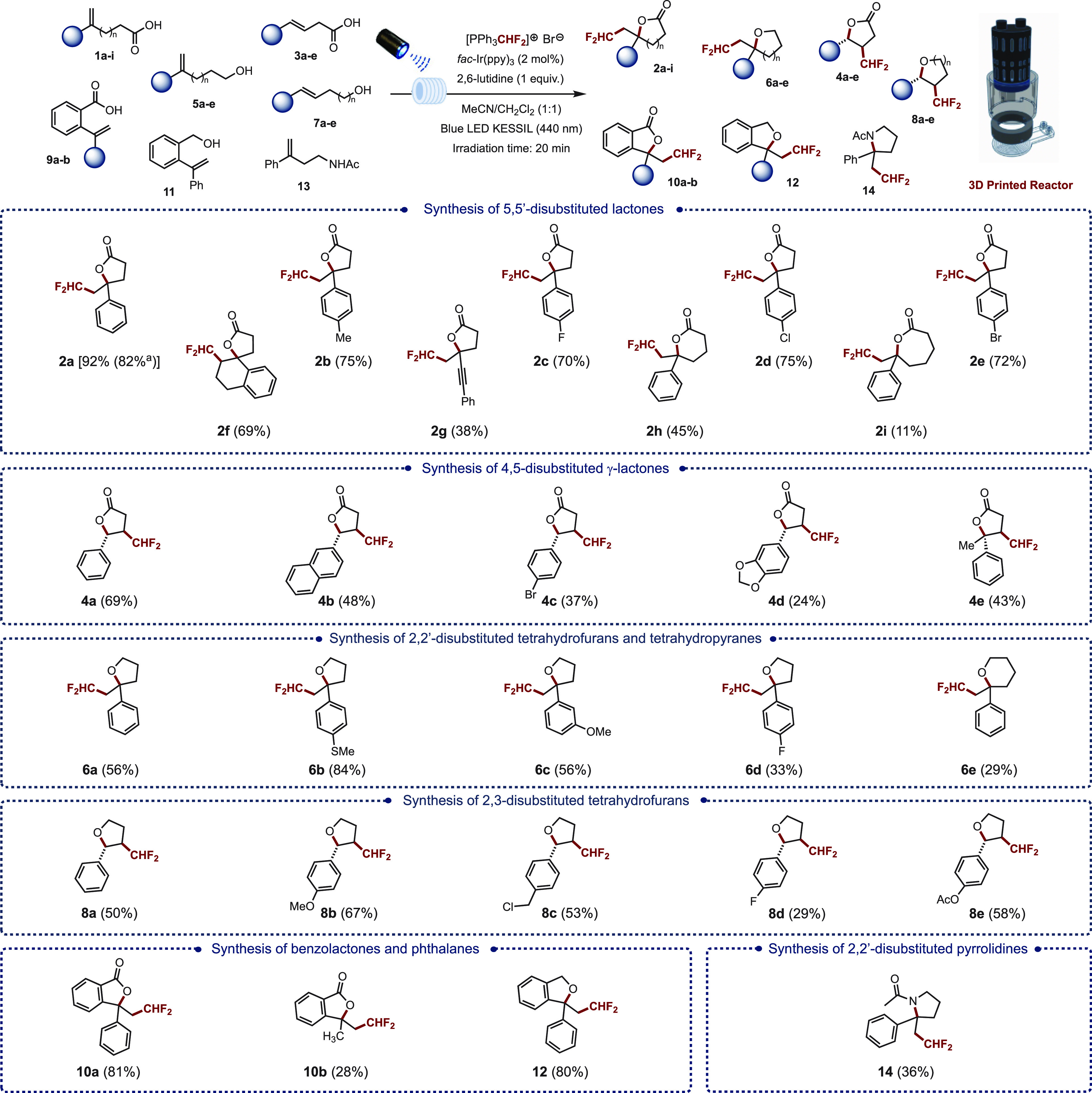
Substrate scope. ^a^Reaction was run on a 1 mmol scale.

The scope was further extended to internal alkenes **3a**–**e** with the objective of forming 4,5-disubstituted γ-lactones. Under the same reaction conditions, 4-phenylbut-3-enoic acid (**3a**) afforded the corresponding difluoromethylated lactone **4a** in 69% yield as a single *trans* stereoisomer. This *trans* diastereoselectivity supported by DFT calculations (*vide infra*) was also observed by Akita and co-workers in their analogous oxy-trifluoromethylation of alkenoic acids.^10a^ Following this result, we successfully extended the method to the naphthyl (**4b**, 48%), *para*-bromo phenyl (**4c**, 37%). and 1,3-benzodioxole (**4d**, 24%) derivatives as well as to a trisubstituted alkene to form the corresponding lactone bearing a quaternary center at the γ position (**4e**, 43%). The method could also be applied to alkenes bearing a pendent alcohol moiety to form the corresponding difluoromethylated tetrahydrofurans. Hence, in the case of terminal alkenes **5a**–**e**, all five γ-quaternary butyrolactones **6a**–**e** were obtained in yields ranging from 33 to 84%. Once again, the method proved compatible with both electron-rich and electron-poor aromatic derivatives; however, it is worth pointing out that the yields were slightly higher with the substrates bearing an electron-rich aromatic ring such as the *para*-methylthio derivative **6b**. Interestingly, the method could also be used to access tetrahydropyran scaffolds, albeit in only moderate yields (**6e**, 29%). In the case of substrates bearing an internal alkene (**7a**–**e**), the corresponding difluoromethylated 2,3-disubstituted tetrahydrofurans **8a**–**e** were obtained as a single *trans* strereoisomer in yields ranging from 29 to 67%. The method was also particularly effective in producing benzolactones (**10a**–**b**, up to 81% yield) and phthalanes (**12**, 80% yield) starting from the corresponding *ortho*-vinyl-substituted benzoic acid and benzyl alcohol precursors, respectively. Finally, the method was successfully applied to a terminal alkene (**13**) bearing a pendent acetamide to form the corresponding pyrrolidine **14**, albeit in only 36% yield.

To confirm the mechanism, we conducted a fluorescence quenching and TEMPO-mediated radical trapping experiment (Figure 3**A**). We found that **dFM**_**2**_ exhibited a greater efficiency in quenching the fluorescence (see SI for more details), while the reaction between **1a** and **dFM**_**2**_ in the presence of TEMPO resulted in the formation of 75% of the difluoromethylated butyrolactone **2a** along with 13% of the TEMPO–CHF_2_ adduct, which strongly supports a one-electron reduction of **dFM**_**2**_ and subsequent decomposition releasing the CHF_2_ radical.

**Figure 3:**
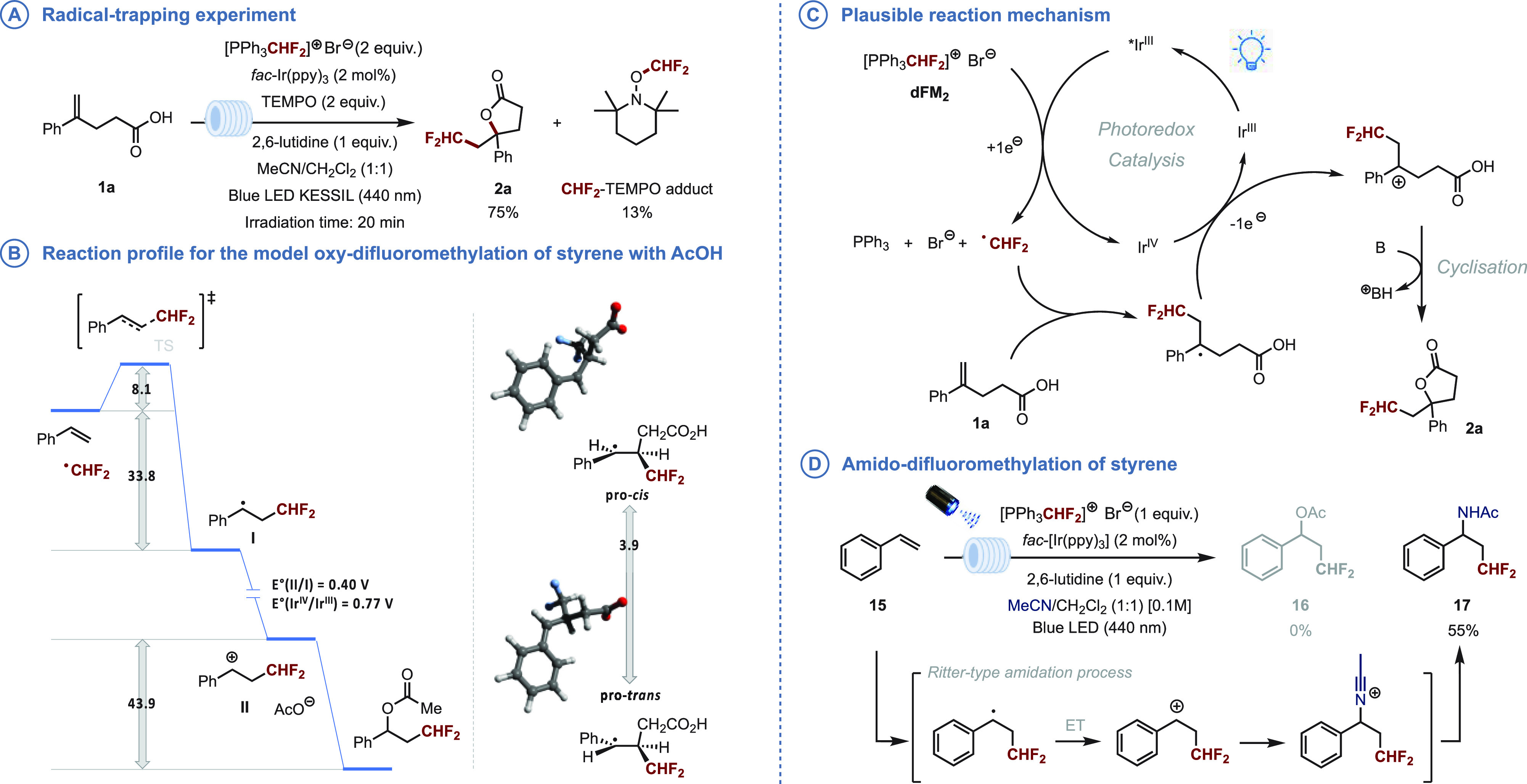
Full survey (Gibbs free energies in kcal/mol, reduction potentials in V referenced to a standard calomel electrode).

DFT studies, performed using the PBE0 functional with Grimme’s D3 dispersion correction, provided further support for the reaction between the photocatalytically generated CHF_2_ and the styrene derivative (Figure 3B). An exhaustive conformational search showed the addition of CHF_2_ radical to be highly exergonic (Δ*G* from −33.1 to −33.8 kcal/mol) with a readily accessible early transition state (Δ*G*^‡^ from 8.1 to 8.4 kcal/mol and F_2_HC–C bond distance 2.57–2.58 Å, see the Supporting Information for more details). A reduction potential *E*° of 0.40 V for the **II**/**I** pair suggest that radical **I** could be easily oxidized to carbocation **II** by the catalyst in its oxidized form (*E*°_Ir(IV)/Ir(III)_ = 0.77 V). Our studies with both inter- and intramolecular attack by carboxylate or carboxylic acid showed a barrierless reaction to form the corresponding ester. This barrierless reaction led us to hypothesize that in substrates leading to diastereomers, the diastereoselectivity of the reaction would be determined by the conformational distribution of radical intermediate **I**. Indeed, a study on the cyclization of compound **3a** showed the most stable conformation among those with a *trans* arrangement of the CHF_2_ and Ph substituents was lower in energy (by 3.9 kcal/mol) than the most stable *cis* conformation. In a more general view, the *trans*-inducing conformations were on average 4.1 kcal/mol lower than the *cis*-inducing ones.

We therefore propose the following mechanism where the excited *Ir(ppy)_3_ undergoes a single-electron-transfer (SET) to the triphenylphosphonium bromide (**dFM**_**2**_), which leads to the release of a CHF_2_ radical (Figure 3**C**). This radical is subsequently added to the alkene of the enoic acid **1a**, leading to the formation of a radi**c**al intermediate. This intermediate is then oxidized by SET from *fac*-Ir^IV^(ppy)_3_ to regenerate the photocatalyst and form the desired carbocation intermediate. The final step of the reaction involves the deprotonation of the carboxylic acid by the base and subsequent cyclization to produce the desired difluoromethylated butyrolactone **2a**.

To demonstrate the scalability of the method, the oxy-difluoromethylation of **1a** was carried out on a millimole scale under continuous flow. The reaction proved easy to set up and the product was isolated in 82% yield, thus highlighting the potential of this low-cost 3D printed standardized photoflow setup for future industrial application.

Finally, we evaluated an intermolecular multicomponent approach that would see styrene (**15**) converted into the corresponding difluoromethylated ester in the presence of acetic acid (Figure 3D). Unfortunately, the formation of the ester was not observed. Instead, we isolated difluoromethylated acetamide **17** in 55% yield. The latter is obtained following a Ritter-type amidation process where the *in situ* generated benzylic carbocation reacts with CH_3_CN to form a nitrilium intermediate, which is eventually hydrolyzed to form the corresponding acetamide.^22^

In summary, we have developed practical, operationally trivial, and highly straightforward access to a variety of CHF_2_-containing heterocycles, including lactones, tetrahydrofurans, tetrahydropyrans, benzolactones, phthalanes, and pyrrolidines, through visible-light-mediated intramolecular oxy-difluoromethylation. The method, which generally offers moderate to excellent yields and excellent regio- and stereoselectivities, can also be used to synthesize difluoromethylated amides through a Ritter-type amidation. Most importantly, the use of low-cost^23^ 3D printed photoflow reactors offers increased safety, cost-saving potential, short reaction times, ease of scale-up, and greater control over reaction parameters, all of which are key points for both academic and industrial applications.

## Data Availability

The data underlying this study are available in the published article and its Supporting Information.

## Supporting Information Available

Details of experimental procedures, characterization data, ^1^H, ^13^C and ^19^F NMR spectra for all products, and detailed computational data (PDF)
