# Supplementary material for: Synthesis of CHF2-Containing Heterocycles through Oxy-difluoromethylation Using Low-Cost 3D Printed PhotoFlow Reactors
Source: Org Lett. 2024 Jan 8;26(14):2877–82. doi: 10.1021/acs.orglett.3c03997 (PMC11020168; doi:10.1021/acs.orglett.3c03997)
Supplement: Supplementary file 1 — ol3c03997_si_001.pdf [file ol3c03997_si_001.pdf]

Supporting Information

Synthesis of CHF_2-Containing Heterocycles through Oxy-difluoromethylation using Low-Cost 3D Printed PhotoFlow Reactors

Jinlei Zhang,[a] Elias Selmi-Higashi,[a] Shen Zhang,[b] Alexandre Jean, [c] Stephen T. Hilton,[d,*] Xacobe C. Cambeiro,[b,*] and Stellios Arseniyadis[a,*]

[a]*Queen Mary University of London, Department of Chemistry, Mile End Road, E1 4NS, London, UK, Email: s.arseniyadis@qmul.ac.uk*

[b]*University of Greenwich, School of Science, Central Ave, ME4 4TB, Gillingham, Chatham, UK, Email: x.cambeiro@greenwich.ac.uk*

[c]*Industrial Research Centre, Oril Industrie, 13 rue Desgenétais, 76210, Bolbec, France*

[d] *UCL School of Pharmacy, University College London, 29-39 Brunswick Square, WC1N 1AX, London, UK, Email: s.hilton@ucl.ac.uk*

Table of Contents

A. Materials and methods **1**

B. Substrate Synthesis **2**

B.1. Synthesis of the difluoromethylating reagents 2

B.2. Synthesis of the photocatalyst 2

B.3. General procedures for the synthesis of the styrene derivatives 3

B.4. Characterization data 6

C. Photocatalyzed oxy-difluoromethylations **21**

C.1. General procedure 21

C.2. mmol scale reaction 22

C.3. Optimization reactions 22

C.4. Characterization data 23

D. Mechanistic investigation **43**

D.1. Radical trapping experiment 43

D.2. Stern-Volmer quenching experiments 44

D.3. Computational details 45

E. ^{1}H NMR, ^{13}C NMR, ^{19}F NMR Spectra **47**

F. References **108**

A. Materials and methods

The reactions were run under argon atmosphere in oven-dried glassware unless otherwise specified. All commercially available compounds (*e.g.* **3a**) were purchased from Sigma Aldrich, Acros Organics, Fluorochem, TCI Chemicals or Alfa Aesar and used as received. Analytical thin layer chromatography (TLC) was performed on silica gel plates (Merck 60F254) visualized either with a UV lamp (254 nm) or by using solutions or $KMnO_4/K_2CO_3$/EtOH in water followed by heating. Flash chromatographies were performed on silica gel (60-230 mesh). Organic extracts were dried over anhydrous Na_2SO_4 or $MgSO_4$. ^{1}H NMR spectra were recorded on a Bruker AVANCE 400 at 400 MHz in $CDCl_3$ and the observed signals are reported as follows: chemical shift in parts per million from tetramethylsilane with the solvent as an internal indicator ($CDCl_3$ δ 7.26 ppm), multiplicity (s = singlet, d = doublet, t = triplet, q = quartet, p = pentet, m = multiplet or overlap of non-equivalent resonances, br = broad), integration. ^{13}C NMR spectra were recorded at 101 MHz in $CDCl_3$ and the observed signals were reported as follows: chemical shift in parts per million from tetramethylsilane with the solvent as an internal indicator ($CDCl_3$ δ 77.16 ppm). Coupling constants, *J*, are reported in Hertz (Hz). Chemical shifts of ^{19}F NMR were expressed in parts per million relatives to the singlet ($\delta = -63.24$) for benzotrifluoride (BTF) as internal standard. Coupling constants, *J*, are reported in Hertz (Hz). ^{19}F NMR spectra were recorded on a Bruker AVANCE 400 at 376.5 MHz in $CDCl_3$ and the observed signals are reported as follows: chemical shift in parts per million (calibrated from the ^{1}H NMR spectra), multiplicity (s = singlet, d = doublet, t = triplet, q = quartet, p = pentet, m = multiplet or overlap of non-equivalent resonances, br = broad), integration. All NMR spectra were obtained at rt. High resolution mass spectra were recorded on an LTQ Orbitrap XL utilising nanospray ionization (NSI), recorded in the positive mode. Infrared spectra were recorded on a Perkin Elmer Spectrum 65 FT-IR spectrometer and are reported in frequency of absorption at the peak maximum (cm^{-1}).

B. Substrate synthesis

B.1. Synthesis of the difluoromethylating reagents

B.1.1. Synthesis of *S*-(difluoromethyl)-*S*-phenyl-*N*-tosylsulfoximine (dFM$_1$)

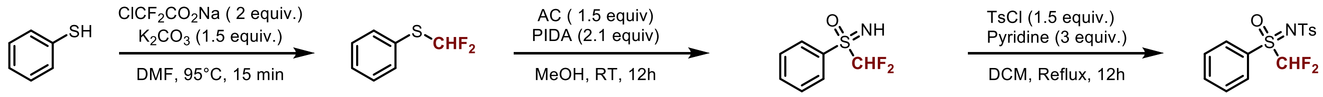

dFM$_1$ was prepared following a reported procedure and isolated as a colourless solid. The analytical data were in accordance with the ones reported in the literature.[1a]

B.1.2. (Difluoromethyl)triphenylphosphonium bromide (dFM$_2$)

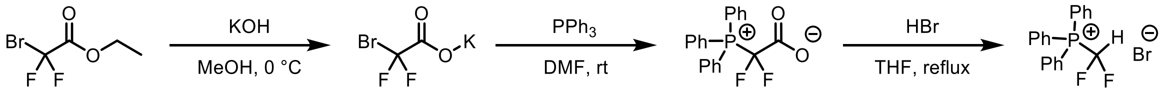

dFM$_3$ was prepared following a reported procedure and isolated as a colourless solid. The analytical data were in accordance with the ones reported in the literature.[1b,c]

B.1.3. (Difluoromethyl)bis(2,5-dimethylphenyl)sulfonium tetrafluoroborate (dFM$_3$)

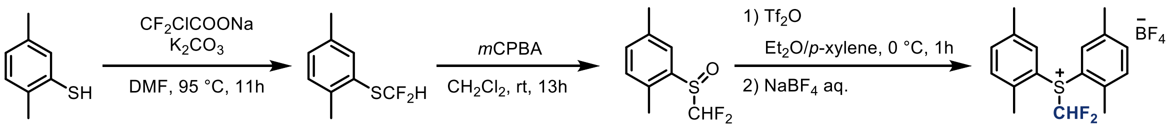

dFM$_3$ was prepared following a reported procedure and isolated as white crystals. The analytical data were in accordance with the ones reported in the literature.[2]

B.2. Synthesis of the photocatalyst

B.2.1. 4CzI PN

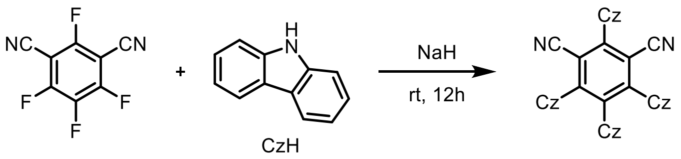

4CzI PN was prepared following a reported procedure and isolated as a white solid. The analytical data were in accordance with the ones reported in the literature.[3]

B.2.2. 10-Phenylphenothiazine

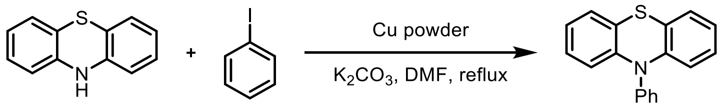

10-Phenylphenothiazine was prepared following a reported procedure and isolated as a white powder. The analytical data were in accordance with the ones reported in the literature.[4]

B.3. General procedures for the synthesis of the styrene derivatives.

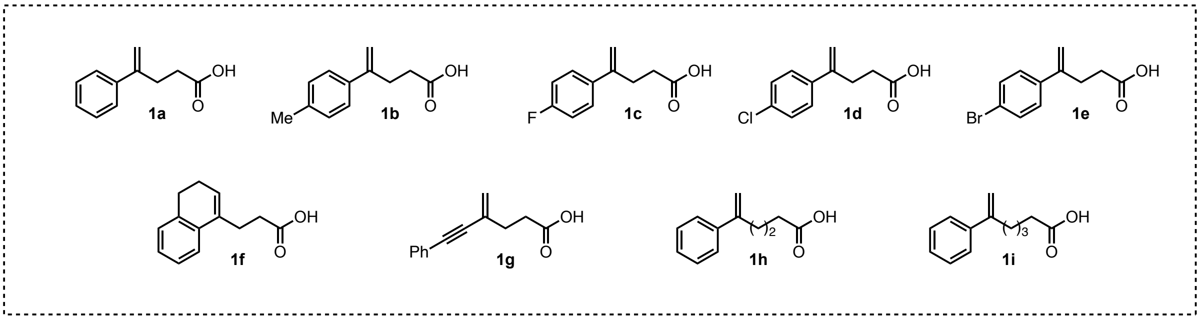

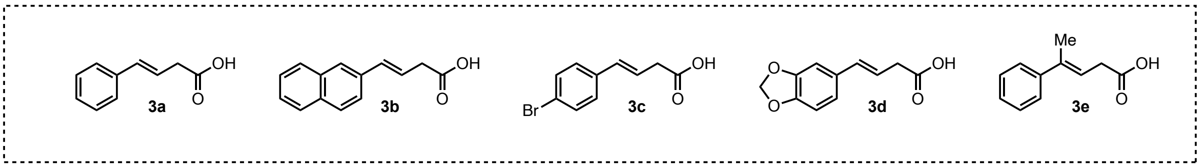

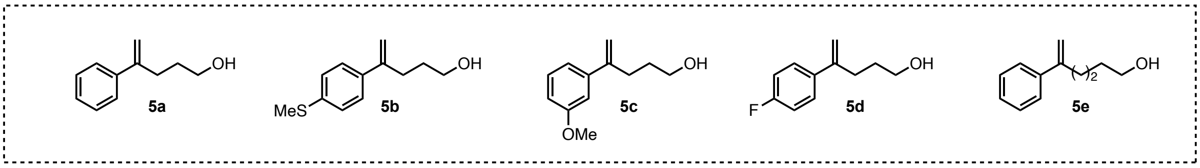

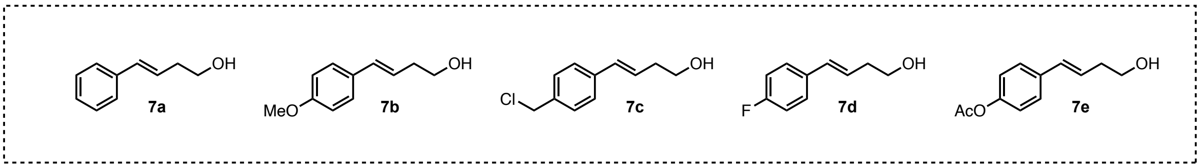

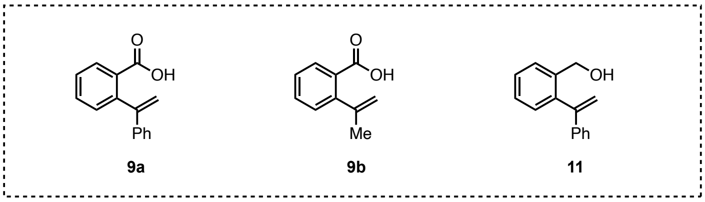

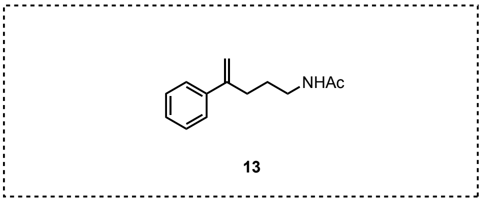

General procedure A for the synthesis of 1a-1e, 1g-1h and **9a-9b.**[5]

To a solution of *t*-BuOK (2.6 equiv.) in dry THF (0.5 M) under nitrogen was added bromo(methyl)triphenylphosphorane (1.3 equiv.) in portions at 0 °C. The mixture was stirred at 0 °C for 30 min and a solution of the corresponding ketone (1.0 equiv.) in dry THF (1 M) was added dropwise. The reaction was stirred at 0 °C for 1 h and at rt overnight. The solvent was then removed in vacuo and the residue diluted with DCM and aqueous NaOH (1 M). The aqueous layer was separated, washed with DCM, and acidified to pH 1 with concentrated HCl. The aqueous layer was extracted twice with DCM and then washed with water, dried over anhydrous $MgSO_4$ and concentrated. The crude product was purified by flash column chromatography over silica gel eluting with DCM/MeOH to give pure enoic acids **1a-1e, 1g-1h** and **9a-9b**.

General procedure B for the synthesis of 3b-3d.[6]

A flame-dried flask charged with (2-carboxyethyl)triphenylphosphonium bromide (1.1 equiv.) and THF (20 mL) was cooled to –20 °C. To the white suspension was added sodium bis(trimethylsilyl)amide (NaHMDS) (2.4 equiv.) dropwise over 5 min. The resulting orange solution was stirred for 30 min and became homogenous gradually. A solution of the corresponding aldehyde (1.0 equiv.) in THF was added by syringe at –20 °C. The resulting mixture was allowed to warm to rt slowly while stirring overnight. The heterogeneous reaction mixture was quenched with water and washed with Et_2O, then the aqueous layer was acidified to pH 1 by dropwise addition of 50% sulfuric acid and then extracted with EtOAc. The combined organic extracts were dried over anhydrous $MgSO_4$, filtered and concentrated under reduced pressure. The acids were subjected to flash column chromatography over silica gel and/or recrystallization.

General procedure C for the synthesis of 5a-5e and 1i.[7]

$Pd(PPh_3)_4$ (347 mg, 0.03 mmol, 3 mol%) was added in a flame-dried thick-walled glass vessel equipped with a stirring bar under an argon atmosphere, followed by the corresponding boronic acid (1.1 mmol, 1.1 equiv.) and alkynyl alcohol (1.0 mmol, 1 equiv.). Dry 1,4-dioxane (2 mL) and AcOH (347 mg, 0.3 mmol, 0.2 equiv.) were then added and the solution was stirred

at rt for 15 min and then at 80 °C for 12 h. Once the reaction was complete (reaction monitored by TLC), the mixture was cooled to rt, and the solvent was removed under reduced pressure. The crude residue was purified by flash column chromatography over silica gel eluting with hexane/EtOAc to afford **5a-5e** and **1i**.

General procedure D for the synthesis of 7a-7e.[8]

Hoveyda-Grubbs 2nd generation catalyst (75 mg, 0.12 mmol, 6 mol%) was added in a flame-dried thick-walled glass vessel equipped with a stirring bar under an argon atmosphere, followed by the corresponding styrene (2.0 mmol, 1 equiv.) and alkenyl alcohol (6.0 mmol, 3 equiv.). Dry DCM (1 mL) was then added, and the reaction mixture was refluxed for 12 h. Once the reaction was complete (reaction monitored by TLC), the solution was cooled to rt, and the solvent was removed under reduced pressure. The crude residue was purified by flash column chromatography over silica gel eluting with hexane/EtOAc to afford **7a-7e**.

B.4. Characterization data

4-Phenylpent-4-enoic acid (1a)

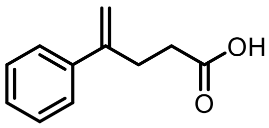

Molecular formula: $C_{11}H_{12}O_2$ **MW =** 176.22 g.mol^{-1}

4-Phenylpent-4-enoic acid was prepared following **General Procedure A**. Purification by flash column chromatography over silica gel and eluting hexane/EtOAc afforded the desired product as a white solid (1.23 g, 6.98 mmol, 90% yield). The analytical data were in accordance with the ones reported in the literature.[5]

^{1}H NMR (400 MHz, CDCl$_3$) δ 11.60 (bs, 1H), 7.46-7.27 (m, 5H,), 5.35 (s, 2H), 2.87 (t, *J* = 7.8 Hz, 2H), 2.56 (dd, *J* = 8.9, 6.7 Hz, 2H).

^{13}C NMR (101 MHz, CDCl$_3$) δ 179.9, 146.6, 140.5, 128.6, 127.8, 126.2, 113.1, 33.1, 30.2.

4-(*p*-Tolyl)pent-4-enoic acid (1b)

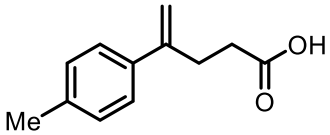

Molecular formula: $C_{12}H_{14}O_2$ **MW =** 190.24 g.mol^{-1}

4-(*p*-Tolyl)pent-4-enoic acid was prepared following **General Procedure A**. Purification by flash column chromatography over silica gel and eluting with hexane/EtOAc afforded the desired product as a white solid (1.1 g, 5.78 mmol, 85% yield). The analytical data were in accordance with the ones reported in the literature.[5]

^{1}H NMR (400 MHz, CDCl$_3$) δ 7.33 (d, *J* = 8.2 Hz, 2H), 7.17 (d, *J* = 8.0 Hz, 2H), 5.33 (d, *J* = 1.0 Hz, 1H), 5.09 (q, *J* = 1.2 Hz, 1H), 2.90-2.83 (m, 2H), 2.61-2.52 (m, 2H), 2.38 (s, 3H).

^{13}C NMR (101 MHz, CDCl$_3$) δ 179.8, 146.4, 137.6, 137.5, 129.2, 126.0, 112.3, 33.2, 30.2, 21.2.

4-(4-Fluorophenyl)pent-4-enoic acid (1c)

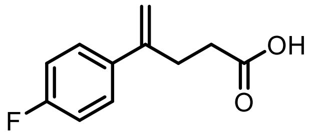

Molecular formula: $C_{11}H_{11}FO_2$ **MW =** 194.21 g.mol^{-1}

4-(4-Fluorophenyl)pent-4-enoic acid was prepared following **General Procedure A**. Purification by flash column chromatography over silica gel and eluting with hexane/EtOAc afforded the desired product as a white solid (427 mg, 2.2 mmol, 86 % yield). The analytical data were in accordance with the ones reported in the literature.[5]

^{1}H NMR (400 MHz, CDCl$_3$) δ 7.41-7.32 (m, 2H), 7.02 (t, *J* = 8.7 Hz, 2H), 5.27 (s, 1H), 5.09 (s, 1H), 2.81 (t, *J* = 7.7 Hz, 2H), 2.51(t, *J* = 7.9 Hz, 2H).

^{13}C NMR (101 MHz, CDCl$_3$) δ 179.7, 162.5 (d, *J* = 246.6 Hz), 145.6, 136.6 (d, J = 3.3 Hz), 127.8 (d, *J* = 8.0 Hz), 115.4 (d, *J* = 21.3 Hz), 113.1, 33.0, 30.3.

^{19}F NMR (376.5 MHz, CDCl$_3$) *δ* –114.8 (m).

4-(4-Chlorophenyl)pent-4-enoic acid (1d)

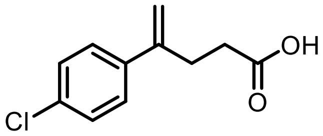

Molecular formula: $C_{11}H_{11}ClO_2$ **MW =** 210.66 g.mol^{-1}

4-(4-Chlorophenyl)pent-4-enoic acid was prepared following **General Procedure A**. Purification by flash column chromatography over silica gel and eluting with hexane/EtOAc afforded the desired product as a white solid (0.98 g, 4.7 mmol, 61 % yield). The analytical data were in accordance with the ones reported in the literature.[5]

^{1}H NMR (400 MHz, CDCl$_3$) δ 7.35-7.28 (m, 4H), 5.31 (s, 1H), 5.12 (d, *J* = 1.3 Hz, 1H,), 2.81 (t, *J* = 7.4 Hz, 2H), 2.52 (m, 2H).

^{13}C NMR (101 MHz, CDCl$_3$) δ 178.8, 145.6, 139.0, 133.7, 128.7, 127.5, 113.7, 32.9, 30.1.

4-(4-Bromophenyl)pent-4-enoic acid (1e)

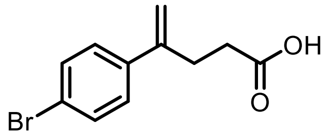

Molecular formula: $C_{11}H_{11}BrO_2$ **MW =** 255.11 g.mol^{-1}

4-(4-Bromophenyl)pent-4-enoic acid was prepared following **General Procedure A**. Purification by flash column chromatography over silica gel and eluting with hexane/EtOAc afforded the desired product as a white solid (1.5 g, 5.88 mmol, 76 % yield). The analytical data were in accordance with the ones reported in the literature.[5]

^{1}H NMR (400 MHz, DMSO) δ 12.18 (s, 1H), 7.59-7.50 (m, 2H), 7.45-7.38 (m, 2H), 5.39 (s, 1H), 5.13 (d, J = 1.4 Hz, 1H), 2.70 (t, J = 7.6 Hz, 2H), 2.36 (dd, J = 8.4, 6.7 Hz, 2H).

^{13}C NMR (101 MHz, DMSO) δ 173.8, 145.4, 139.3, 131.4, 128.0, 120.8, 113.3, 32.5, 29.4.

3-(3,4-Dihydronaphthalen-1-yl)propanoic acid (1f)

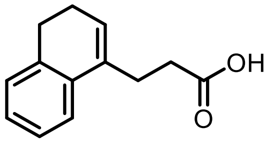

Molecular formula: $C_{13}H_{14}O_2$ **MW =** 202.25 g.mol^{-1}

A mixture of α-tetralone (4 mL, 30 mmol, 1.2 equiv.), diethyl succinate (4.2 mL, 25 mmol, 1 equiv.), *t*-BuOK (5 g, 45 mmol, 1.8 equiv.) and *t*-butanol (20 mL) was heated under reflux for 45 min. The solvent was evaporated and the crude residue poured into an HCl solution (8 N, 8 mL), and extracted with diethyl ether (2 × 20 mL). The combined organic layers were washed with brine, dried over anhydrous $MgSO_4$, and evaporated under reduced pressure to give a brown oil, which was subsequently heated under reflux in a 1:1 mixture of concentrated HCl (20mL) and AcOH (20 mL) for 3 h. The reaction mixture was concentrated, and the crude residue poured into water (50 mL) and extracted with diethyl ether (2 × 40 mL). The combined organic layers were washed with aqueous NaOH (3 N, 12 mL) and water (2 × 20 mL). The combined aqueous phases were acidified with H_2SO_4 (3 N, 22 mL), extracted with diethyl ether (2 × 15 mL), and the combined organic layers were washed with water (2 × 15 mL) and

a saturated aqueous solution of brine (15 mL), dried over anhydrous $MgSO_4$, and concentrated under reduced pressure to give the crude product as a brown solid. Crystallization from EtOAc/hexane yielded 1.0 g (20%) of **1g** as a white solid. The analytical data were in accordance with the ones reported in the literature.[9]

^{1}H NMR (400 MHz, $CDCl_3$) δ 7.29-7.08 (m, 4 H), 5.91 (t, *J* = 4.5 Hz, 1 H), 2.91-2.68 (m, 4 H), 2.67-2.58 (m, 2 H), 2.34-2.19 (m, 2 H).

^{13}C NMR (101 MHz, $CDCl_3$) δ 179.6, 136.7, 134.6, 134.1, 127.7, 126.8, 126.4, 125.4, 122.2, 33.2, 28.2, 27.5, 23.0.

4-Methylene-6-phenylhex-5-ynoic acid (1g)

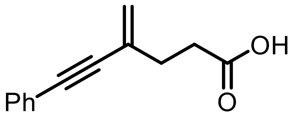

Molecular formula: $C_{13}H_{12}O_2$ **MW =** 200.24 g.mol^{-1}

4-Methylene-6-phenylhex-5-ynoic acid was prepared following **General Procedure A**. Purification by flash column chromatography over silica gel and eluting hexane/EtOAc afforded the desired product as a brown solid (0.26 g, 1.3 mmol, 65 % yield). The analytical data were in accordance with the ones reported in the literature.[5]

^{1}H NMR (400 MHz, $CDCl_3$) δ 7.48-7.41 (m, 2H), 7.35-7.29 (m, 3H), 5.47 (d, *J* = 1.5 Hz, 1H), 5.38 (d, *J* = 1.4 Hz, 1H), 2.68 (ddd, *J* = 8.4, 7.0, 1.7 Hz, 2H), 2.63-2.54 (m, 2H).

^{13}C NMR (101 MHz, $CDCl_3$) δ 179.2, 131.7, 129.6, 128.4, 128.5, 123.0, 122.3, 90.2, 88.7, 33.0, 32.1.

5-Phenylhex-5-enoic acid (1h)

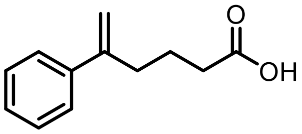

Molecular formula: $C_{12}H_{14}O_2$ **MW =** 190.24 g.mol^{-1}

5-Phenylhex-5-enoic acid was prepared following **General Procedure A**. Purification by flash column chromatography over silica gel and eluting with hexane/EtOAc afforded the desired product as a white solid (0.40 g, 2.1 mmol, 26 % yield). The analytical data were in accordance with the ones reported in the literature.[5]

^{1}H NMR (400 MHz, CDCl$_3$) δ 7.45-7.41 (m, 2H), 7.38-7.33 (m, 2H), 7.32-7.27 (m, 1H), 5.35 (d, *J* = 1.3 Hz, 1H), 5.12 (d, *J* = 1.4 Hz, 1H), 2.61 (td, *J* = 7.5, 1.2 Hz, 2H), 2.41 (t, *J* = 7.4 Hz, 2H), 1.83 (p, *J* = 7.5 Hz, 2H).

^{13}C NMR (101 MHz, CDCl$_3$) δ 180.3, 147.4, 140.8, 128.5, 127.6, 126.2, 113.2, 34.5, 33.4, 23.1.

6-Phenylhept-6-enoic acid (1i)

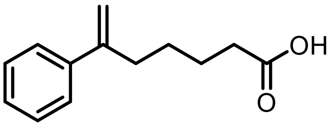

Molecular formula: $C_{13}H_{16}O_2$ **MW =** 204.27 g.mol^{-1}

6-Phenylhept-6-enoic acid was prepared following **General Procedure C**. Purification by flash column chromatography over silica gel and eluting with hexane/EtOAc afforded the desired product as a white solid (116 mg, 0.57 mmol, 57%). The analytical data were in accordance with the ones reported in the literature.[10]

^{1}H NMR (300 MHz, CDCl$_3$) δ 11.5 (broad s, 1H), 7.62-7.22 (m, 5H), 5.40 (s, 1H), 5.18 (s, 1H), 2.63 (t, *J* = 7.3 Hz, 2H), 2.44 (t, *J* = 7.3 Hz, 2H), 1.85-1.71 (m, 2H), 1.68-1.54 (m, 2H).

^{13}C NMR (75 MHz, CDCl$_3$) δ 180.5, 148.1, 141.2, 128.4, 127.5, 126.2, 112.6, 35.0, 34.1, 27.6, 24.4.

(*E*)-4-(Naphthalen-2-yl)but-3-enoic acid (3b)

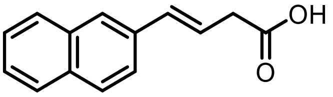

Molecular formula: $C_{14}H_{12}O_2$ **MW =** 212.25 g.mol^{-1}

(*E*)-4-(Naphthalen-2-yl)but-3-enoic acid was prepared following **General Procedure B**. Purification by flash column chromatography over silica gel and eluting with DCM/MeOH (100:2) to hexane/EtOAc (6:1) with less than 10% AcOH afforded the desired product as a white solid (121 mg, 0.57 mmol, 57%). The analytical data were in accordance with the ones reported in the literature.[11]

^{1}H NMR (400 MHz, DMSO) δ 12.39 (s, 1H), 7.87 (d, *J* = 3.3 Hz, 3H), 7.83 (s, 1H), 7.68 (d, *J* = 8.5 Hz, 1H), 7.53-7.44 (m, 2H), 6.67 (d, *J* = 15.9 Hz, 1H), 6.56-6.40 (m, 1H), 3.27 (d, *J* = 6.5 Hz, 2H).

^{13}C NMR (101 MHz, DMSO) δ 173.1, 134.8, 133.7, 132.9, 132.8, 128.6, 128.3, 128.0, 126.8, 126.3, 126.0, 124.3, 123.9, 38.4.

(*E*)-4-(4-Bromophenyl)but-3-enoic acid (3c)

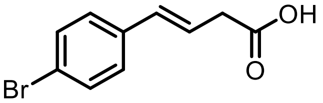

Molecular formula: $C_{10}H_9BrO_2$ **MW =** 241.08 g.mol^{-1}

(*E*)-4-(4-Bromophenyl)but-3-enoic acid was prepared following **General Procedure B**. Purification by flash column chromatography over silica gel and eluting with hexane/EtOAc (5:1) with less than 10% AcOH afforded the desired product as a colourless oil (121 mg, 0.57 mmol, 43%). The analytical data were in accordance with the ones reported in the literature.[11]

^{1}H NMR (400 MHz, $CDCl_3$) δ 7.70-7.75 (m, 2H), 7.35-7.40 (m, 2H), 6.62 (d, *J* = 15.9 Hz, 1H,), 6.37 (dd, *J* = 15.1, 7.9 Hz, 1H), 3.31 (d, *J* = 7.1 Hz, 2H).

^{13}C NMR (101 MHz, $CDCl_3$) δ 177.0, 135.7, 133.0, 131.8, 128.0, 121.8, 121.7,37.9.

(*E*)-4-(Benzo[d][1,3]dioxol-5-yl)but-3-enoic acid (3d)

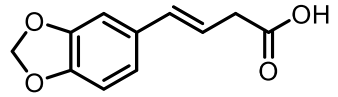

Molecular formula: $C_{11}H_{10}O_4$ **MW =** 206.20 g.mol^{-1}

(*E*)-4-(Benzo[d][1,3]dioxol-5-yl)but-3-enoic acid was prepared following **General Procedure B**. Purification by flash column chromatography over silica gel and eluting with hexane/EtOAc (5:1) with less than 10% AcOH afforded the desired product as a colourless oil (121 mg, 0.57 mmol, 30%). The analytical data were in accordance with the ones reported in the literature.[11]

^{1}H NMR (400 MHz, CDCl$_3$) δ 6.92 (d, *J* = 1.7 Hz, 1H), 6.80 (d, *J* = 8.0, 1.7 Hz, 1H), 6.74 (d, *J* = 8.0 Hz, 1H), 6.42 (d, *J* = 15.9 Hz, 1H), 6.22-6.14 (m, 1H), 5.95 (s, 2H), 3.26 (dd, *J* = 7.1, 1.5 Hz, 2H).

^{13}C NMR (101 MHz, CDCl$_3$) δ 176.8, 148.2, 147.4, 133.7, 131.3, 121.1, 119.1, 108.4, 105.8, 101.2, 37.9.

(*E*)-4-Phenylpent-3-enoic acid (3e)

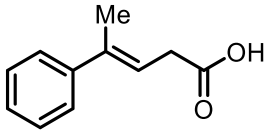

Molecular formula: $C_{11}H_{12}O_2$ **MW =** 176.22 g.mol^{-1}

A solution of aldehyde (0.10 mmol, 1.0 equiv.), malonic acid (0.15 mmol, 1.5 equiv.), and NEt_3 (20 mL) was refluxed for 2 h and then cooled to rt. The solution was diluted with Et_2O (100 mL), washed with 3 N HCl (100 mL) and treated with 10% NaOH (100 mL). The aqueous layer was washed with ether (50 mL) and acidified (pH 1) with 3 N HCl. The solution was extracted with ether (3 × 50 mL). The combined extracts were washed with a saturated aqueous solution of NaCl (50 mL) and dried over anhydrous $MgSO_4$ and evaporated in vacuo. The residue was purified by recrystallization from hexane/EtOAc to give the product as a white solid (121 mg, 0.57 mmol, 57%). The analytical data were in accordance with the ones reported in the literature.[12]

^{1}H NMR (400 MHz, CDCl$_3$) δ 7.40 (d, *J* = 7.7 Hz, 2H), 7.32 (t, *J* = 7.5 Hz, 2H), 7.26-7.24 (m, 1H), 5.93 (t, *J* = 7.0 Hz, 1H), 3.31 (d, *J* = 7.1 Hz, 2H), 2.07 (s, 3H).

^{13}C NMR (101 MHz, CDCl$_3$) δ 178.3, 142.9, 138.7, 128.2, 127.2, 125.8, 118.3, 34.1, 16.2.

4-Phenylpent-4-en-1-ol (5a)

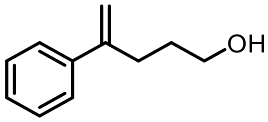

Molecular formula: $C_{11}H_{14}O$ **MW =** 162.23 g.mol^{-1}

4-Phenylpent-4-en-1-ol was prepared following **General Procedure C**. Purification by flash column chromatography over silica gel and eluting with hexane/EtOAc (4:1) afforded the desired product as a colourless oil (130 mg, 0.79 mmol, 80%). The analytical data were in accordance with the ones reported in the literature.[7]

^{1}H NMR (400 MHz, CDCl$_3$) δ 7.35-7.31 (m, 2H), 7.27-7.22 (m, 2H), 7.21-7.16 (m, 1H), 5.21 (d, *J* = 1.3 Hz, 1H), 5.01 (q, *J* = 1.3 Hz, 1H), 3.57 (t, *J* = 6.5 Hz, 2H), 2.52 (td, *J* = 7.7, 1.0 Hz, 2H), 1.70-1.57 (m, 2H).

^{13}C NMR (101 MHz, CDCl$_3$) δ 148.1, 141.1, 128.4, 127.5, 126.2, 112.7, 62.5, 31.7, 31.3.

4-[4-(Methylthio)phenyl]pent-4-en-1-ol (5b)

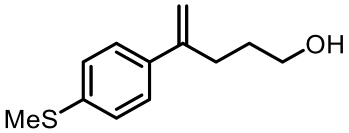

Molecular formula: $C_{12}H_{16}OS$ **MW =** 208.32 g.mol^{-1}

4-[4-(Methylthio)phenyl]pent-4-en-1-ol was prepared following **General Procedure C**. Purification by flash column chromatography over silica gel and eluting with hexane/EtOAc (5:1) afforded the desired product as a colourless oil (137 mg, 0.66 mmol, 66%).

^{1}H NMR (400 MHz, CDCl$_3$) δ 7.37-7.32 (m, 2H), 7.24-7.19 (m, 2H), 5.29 (d, *J* = 1.3 Hz, 1H), 5.07 (q, *J* = 1.3 Hz, 1H), 3.66 (t, *J* = 6.4 Hz, 2H), 2.58 (td, *J* = 7.7, 1.0 Hz, 2H), 2.49 (s, 3H), 1.73 (m, 1H).

^{13}C NMR (101 MHz, $CDCl_3$) δ 147.3, 137.9, 137.7, 126.7 (four carbon signals are overlapping), 112.3, 62.5, 31.6, 31.3, 16.0.

IR (neat, cm^{-1}) 3345, 2920, 1594, 1493, 1038, 894, 826.

HRMS (ESI) *m/z* Calcd. for $C_{12}H_{17}OS^+$ $[M+H]^+$: 209.0995; found: 209.0999.

4-(3-Methoxyphenyl)pent-4-en-1-ol (5c)

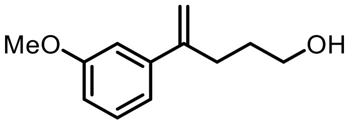

Molecular formula: $C_{12}H_{16}O_2$ **MW =** 192.26 $g.mol^{-1}$

4-(3-Methoxyphenyl)pent-4-en-1-ol was prepared following **General Procedure C**. Purification by flash column chromatography over silica gel and eluting with hexane/EtOAc (5:1) afforded the desired product as a colourless oil (164 mg, 0.85 mmol, 85%). The analytical data were in accordance with the ones reported in the literature.[7]

^{1}H NMR (400 MHz, $CDCl_3$) δ 7.24 (t, *J* = 7.9 Hz, 1H), 7.01 (ddd, *J* = 7.7, 1.6, 0.9 Hz, 1H), 6.97-6.94 (m, 1H), 6.82 (ddd, *J* = 8.2, 2.6, 0.9 Hz, 1H), 5.30 (d, *J* = 1.4 Hz, 1H), 5.09 (dd, *J* = 2.7, 1.3 Hz, 1H), 3.82 (s, 3H), 3.65 (t, *J* = 6.5 Hz, 2H), 2.58 (td, *J* = 7.7, 0.9 Hz, 2H), 1.72 (dq, *J* = 14.0, 6.5 Hz, 2H).

^{13}C NMR (101 MHz, $CDCl_3$) δ 159.7, 148.0, 142.7, 129.4, 118.8, 112.8, 112.7, 112.3, 62.4, 55.3, 31.7, 31.2.

4-(4-Fluorophenyl)pent-4-en-1-ol (5d)

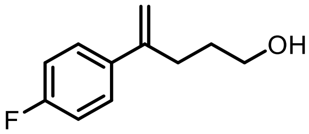

Molecular formula: $C_{11}H_{13}FO$ **MW =** 180.22 $g.mol^{-1}$

4-(4-Fluorophenyl)pent-4-en-1-ol was prepared following **General Procedure C**. Purification by flash column chromatography over silica gel and eluting with hexane/EtOAc (4:1) afforded the desired product as a colourless oil (47 mg, 0.26 mmol, 26%). The analytical data were in accordance with the ones reported in the literature.[7]

^{1}H NMR (400 MHz, CDCl$_3$) δ 7.40-7.34 (m, 2H), 7.06-6.97 (m, 2H), 5.24 (d, *J* = 0.9 Hz, 1H), 5.08 (d, *J* = 1.2 Hz, 1H), 3.66 (t, *J* = 6.4 Hz, 2H), 2.57 (td, *J* = 7.7, 1.0 Hz, 2H), 1.71 (ddt, *J* = 8.6, 7.4, 6.5 Hz, 2H), 1.47 (s, 1H).

^{13}C NMR (101 MHz, CDCl$_3$) δ 162.4 (d, *J* = 246.2 Hz), 147.1, 137.2 (d, *J* = 3.3 Hz), 127.8 (d, *J* = 7.9 Hz), 115.3 (d, *J* = 21.3 Hz), 112.6 (d, *J* = 1.1 Hz), 62.5, 31.8, 31.2.

^{19}F NMR (376.5 MHz, CDCl$_3$) *δ* –115.3 (m).

5-Phenylhex-5-en-1-ol (5e)

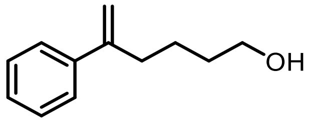

Molecular formula: $C_{12}H_{16}O$ **MW =** 176.26 g.mol^{-1}

5-Phenylhex-5-en-1-ol was prepared following **General Procedure C**. Purification by flash column chromatography over silica gel and eluting with hexane/EtOAc (5:1) afforded the desired product as a colourless oil (159 mg, 0.9 mmol, 90%). The analytical data were in accordance with the ones reported in the literature.[7]

^{1}H NMR (400 MHz, CDCl$_3$) δ 7.34-7.30 (m, 2H), 7.27-7.21 (m, 2H), 7.20-7.15 (m, 1H), 5.19 (d, *J* = 1.5 Hz, 1H), 4.99 (q, *J* = 1.4 Hz, 1H), 3.54 (t, *J* = 6.4 Hz, 2H), 2.49-2.43 (m, 2H), 1.56-1.40 (m, 4H).

^{13}C NMR (101 MHz, CDCl$_3$) δ 148.5, 141.4, 128.4, 127.5, 126.2, 112.5, 62.9, 35.2, 32.5, 24.5.

(*E*)-4-Phenylbut-3-en-1-ol (7a)

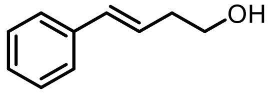

Molecular formula: $C_{10}H_{12}O$ **MW =** 148.21 g.mol^{-1}

(*E*)-4-Phenylbut-3-en-1-ol was prepared following **General Procedure D**. Purification by flash column chromatography over silica gel and eluting with hexane/EtOAc (6:1) afforded the

desired product as a colourless oil (547 mg, 3.7 mmol, 82%). The analytical data were in accordance with the ones reported in the literature.[13]

^{1}H NMR (400 MHz, CDCl$_3$) δ 7.32 (m, 5H), 6.52 (d, *J* = 15.6 Hz, 1H), 6.23 (dt, *J* = 15.6, 7.2 Hz, 1H), 3.78 (t, *J* = 6 Hz, 2H), 2.5 (dt, *J* = 7.2, 6 Hz, 2H).

^{13}C NMR (101 MHz, CDCl$_3$) δ 137.2, 132.8, 128.6, 127.3, 126.4, 126.1, 62.0, 36.3

(*E*)-4-(4-Methoxyphenyl)but-3-en-1-ol (7b)

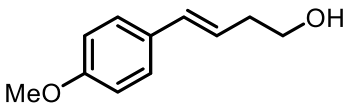

Molecular formula: $C_{11}H_{14}O_2$ **MW =** 178.23 g.mol^{-1}

(*E*)-4-(4-Methoxyphenyl)but-3-en-1-ol was prepared following **General Procedure D**. Purification by flash column chromatography over silica gel and eluting with hexane/EtOAc (4:1) afforded the desired product as a colourless oil (103 mg, 0.58 mmol, 58%). The analytical data were in accordance with the ones reported in the literature.[13]

^{1}H NMR (400 MHz, CDCl$_3$) δ 7.33-7.27 (m, 2H), 6.87-6.81 (m, 2H), 6.44 (d, *J* = 15.9 Hz, 1H), 6.05 (dt, *J* = 15.8, 7.2 Hz, 1H), 3.80 (s, 3H), 3.73 (t, *J* = 6.3 Hz, 2H), 2.46 (qd, *J* = 6.3, 1.4 Hz, 2H).

^{13}C NMR (101 MHz, CDCl$_3$) δ 159.1, 132.4, 130.2, 127.3, 124.2, 114.1, 62.2, 55.4, 36.5.

(*E*)-4-[4-(Chloromethyl)phenyl]but-3-en-1-ol (7c)

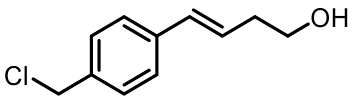

Molecular formula: $C_{11}H_{13}ClO$ **MW =** 196.67 g.mol^{-1}

(*E*)-4-[4-(Chloromethyl)phenyl]but-3-en-1-ol was prepared following **General Procedure D**. Purification by flash column chromatography over silica gel and eluting with hexane/EtOAc (3:1) afforded the desired product as a white solid (102 mg, 0.52 mmol, 52%).

^{1}H NMR (400 MHz, CDCl$_3$) *δ* 7.37-7.30 (m, 4H), 6.49 (d, *J* = 15.9 Hz, 1H), 6.23 (dt, *J* = 15.9, 7.1 Hz, 1H), 4.57 (s, 2H), 3.76 (t, *J* = 6.3 Hz, 2H), 2.53-2.45 (m, 2H).

^{13}C NMR (101 MHz, CDCl$_3$) δ 137.6, 136.5, 132.2, 129.0, 127.4, 126.5, 62.1, 46.2, 36.5.

IR (neat, cm^{-1}) 3366, 2928, 1610, 1512, 1264,1043, 966, 731, 226.

HRMS (ESI) *m/z*: Calcd. for $C_{11}H_{14}ClO^+[M+H]^+$: 197.0733; found: 197.0734.

(*E*)-4-(4-Fluorophenyl)but-3-en-1-ol (7d)

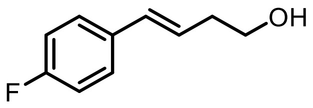

Molecular formula: $C_{10}H_{11}FO$ **MW =** 166.20 g.mol^{-1}

(*E*)-4-(4-Fluorophenyl)but-3-en-1-ol was prepared following **General Procedure D**. Purification by flash column chromatography over silica gel and eluting with hexane/EtOAc (5:1) afforded the desired product as a colourless oil (75 mg, 0.45 mmol, 45%). The analytical data were in accordance with the ones reported in the literature.[13]

^{1}H NMR (400 MHz, CDCl$_3$) δ 7.31 (dd, *J* = 8.7, 5.4 Hz, 2H), 6.99 (t, *J* = 8.7 Hz, 2H), 6.46 (d, *J* = 15.9 Hz, 1H), 6.12 (dt, *J* = 15.8, 7.1 Hz, 1H), 3.75 (t, *J* = 6.3 Hz, 2H), 2.47 (td, *J* = 7.4, 1.2 Hz, 2H).

^{13}C NMR (101 MHz, CDCl$_3$) δ 162.3 (d, *J* = 246.2 Hz), 133.6 (d, *J* = 3.3 Hz), 131.7, 127.7 (d, *J* = 7.9 Hz), 126.3 (d, *J* = 2.2 Hz), 115.5 (d, *J* = 21.5 Hz), 62.1, 36.5.

^{19}F NMR (377 MHz, CDCl$_3$) δ –115.1 (tt, *J* = 8.6, 5.4 Hz).

(*E*)-4-(4-Hydroxybut-1-en-1-yl)phenyl acetate (7e)

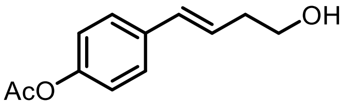

Molecular formula: $C_{12}H_{14}O_3$ **MW =** 206.24 g.mol^{-1}

(*E*)-4-(4-Hydroxybut-1-en-1-yl)phenyl acetate following **General Procedure D**. Purification by flash column chromatography over silica gel and eluting with hexane/EtOAc (3:1) afforded the desired product as a white solid (134 mg, 0.65 mmol, 65%).

^{1}H NMR (400 MHz, CDCl$_3$) δ 7.35 (d, *J* = 8.5 Hz, 2H), 7.02 (d, *J* = 8.6 Hz, 2H), 6.47 (d, *J* = 15.9 Hz, 1H), 6.16 (dt, *J* = 15.8, 7.1 Hz, 1H), 3.75 (t, *J* = 6.3 Hz, 2H), 2.48 (qd, *J* = 6.4, 1.4 Hz, 2H), 2.29 (s, 3H).

^{13}C NMR (101 MHz, CDCl$_3$) δ 169.6, 149.9, 135.2, 131.9, 127.1, 126.8, 121.8, 62.1, 36.5, 21.3.

IR ν (cm^{-1}): 2925, 2852, 1751, 1509, 1370., 1162, 906, 641.

HRMS (ESI) *m/z* Calcd. for $C_{12}H_{15}O_3^+$ [M+H]$^+$: 207.1021; found: 207.1024.

M.P. 216-218 °C

2-(1-Phenylvinyl)benzoic acid (9a)

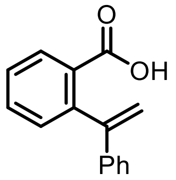

Molecular formula: $C_{15}H_{12}O_2$ **MW =** 224.26 g.mol^{-1}

2-(1-Phenylvinyl)benzoic acid was prepared following **General Procedure A**. Purification by flash column chromatography over silica gel and eluting with to hexane/EtOAc afforded the desired product as a white solid (0.68 g, 3.0 mmol, 40 % yield). The analytical data were in accordance with the ones reported in the literature.[5]

^{1}H NMR (400 MHz, CDCl$_3$) δ 7.93 (dd, *J* = 7.8, 1.4 Hz, 1H), 7.57 (td, *J* = 7.5, 1.5 Hz, 1H), 7.43 (td, *J* = 7.6, 1.4 Hz, 1H), 7.38 (dd, *J* = 7.6, 1.3 Hz, 1H), 7.28-7.18 (m, 5H), 5.67 (d, *J* = 1.0 Hz, 1H), 5.23 (d, *J* = 1.0 Hz, 1H).

^{13}C NMR (101 MHz, CDCl$_3$) δ 172.4, 149.6, 143.7, 141.0, 132.5, 131.7, 130.8, 129.6, 128.2, 127.8, 127.6, 127.0, 114.5

2-(Prop-1-en-2-yl)benzoic acid (9b)

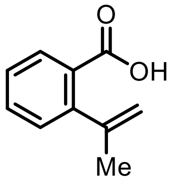

Molecular formula: $C_{10}H_{10}O_2$ **MW =** 162.19 g.mol^{-1}

2-(Prop-1-en-2-yl)benzoic acid was prepared following **General Procedure A**. Purification by flash column chromatography over silica gel and eluting with hexane/EtOAc afforded the

desired product as a white solid (1.9 g, 11.7 mmol, 89 % yield). The analytical data were in accordance with the ones reported in the literature.[5]

^{1}H NMR (400 MHz, CDCl$_3$) δ 7.96 (d, *J* = 7.6 Hz, 1H), 7.51 (t, *J* = 7.5 Hz, 1H), 7.36 (t, *J* = 7.6 Hz, 1H), 7.27 (d, *J* = 7.5 Hz, 1H), 5.13 (s, 1H), 4.90 (s, 1H), 2.13 (s, 3H).

^{13}C NMR (101 MHz, CDCl$_3$) δ 173.7, 146.8, 146.5, 132.8, 130.9, 129.9, 128.2, 127.2, 114.1, 24.5.

2-(1-Phenylvinyl)phenylmethylalcohol (11)

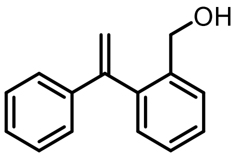

Molecular formula: $C_{15}H_{14}O$ **MW =** 210.28 g.mol^{-1}

To a solution of methyl 2-(1-phenylvinyl)benzoate (330 mg, 1.38 mmol, 1.0 equiv.) in PhMe (1.5 mL) at −50 °C under N_2 was added dropwise DIBAL-H (1.0 mmol in hexane, 3 mmol, 2.2 equiv.) over 10 min. The reaction mixture was stirred at the same temperature for 2 h then warmed to 0 °C and diluted with Et_2O (2 mL). The reaction was quenched with the addition of H_2O (0.2 mL), an aqueous solution of NaOH (15%, 0.2 mL), and H_2O (0.4 mL) in sequence. The mixture was then warmed to rt and stirred for an additional 15 min and dried over anhydrous $MgSO_4$. The resulting mixture was stirred for another 15 min and filtered through a pad of silica with EtOAc (3 × 50 mL). The filtrate was concentrated in vacuo and the crude residue was eventually purified by flash column chromatography over silica gel eluting with hexane/EtOAc (4:1) to afforded [2-(1-phenylvinyl)phenyl]methanol **11** as a colourless oil (7.26 g, 1.25 mmol, 91%). The analytical data were in accordance with the ones reported in the literature.[14]

^{1}H NMR (400 MHz, CDCl$_3$) *δ* 7.52-7.45 (m, 2H), 7.39-7.27 (m, 6H), 7.22 (dd, *J* = 9.2, 7.6 Hz, 2H), 5.82-5.67 (m, 1H), 5.27-5.21 (m, 1H), 4.41 (s, 2H).

^{13}C NMR (101 MHz, CDCl$_3$) δ 148.3, 140.6, 140.4, 138.6, 130.2, 128.5, 128.0, 127.5, 126.5, 115.6, 63.1.

N-(4-Phenylpent-4-en-1-yl)acetamide (13)

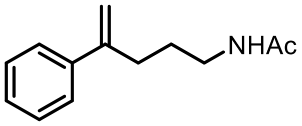

Molecular formula: $C_{13}H_{17}NO$ **MW =** 203.29 g.mol^{-1}

To a round bottom flask added 4-phenylpent-4-en-1-amine (190 mg, 1.18 mmol, 1.0 equiv.) and NEt_3 (238.5 mg, 2.36 mmol, 2.0 equiv.) in DCM (2.5 mL) at 0 °C. Then acetyl chloride (185 mg, 2.36 mmol, 2.0 equiv.) was added over 0.5 h. The mixture was allowed to warm to rt and stirred for 24 h. After that, a 1M aqueous solution of HCl (2.5 mL) was added, and the mixture was extracted with *tert*-butyl methyl ether (2 × 10 mL). The combined organic phases were dried over anhydrous $MgSO_4$ and concentrated. The resultant crude amide was purified by flash column chromatography over silica gel eluting with DCM/MeOH (9:1) to afford the desired amide as a colourless oil (139 mg, 0.68 mmol, 58%).[15]

^{1}H NMR (500 MHz, $CDCl_3$) δ δ 7.44-7.04 (m, 5H), 5.50 (br, 1H), 5.34-5.17 (m, 1H), 5.08 (q, *J* = 6.7 Hz, 1H), 3.26 (tt, *J* = 14.0, 7.0 Hz, 2H), 2.54 (dq, *J* = 14.2, 7.1 Hz, 2H), 1.72-1.48 (m, 2H).

^{13}C NMR (101 MHz, $CDCl_3$) δ δ 147.7, 140.9, 128.5, 127.6, 126.2, 113.0, 39.4, 32.8, 28.1, 23.4.

C. Photocatalyzed oxy-difluoromethylations

C.1 General procedure

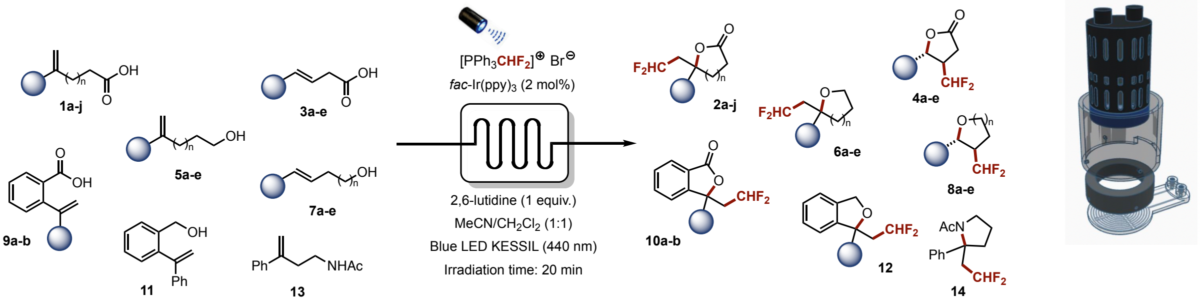

fac-Ir(ppy)$_3$ (2.6 mg, 2 mol%), (difluoromethyl)triphenylphosphonium bromide (**dFM$_2$**) (157 mg, 0.4 mmol, 2.0 equiv.), the substrate (0.2 mmol, 1.0 equiv.), DCM/MeCN (1:1, 2 mL in total) and 2,6-lutidine (23 µL, 0.2 mmol, 1.0 equiv.) were added into a microwave tube under argon. The mixture was degassed by three freeze-pump-thaw cycles and backfilled with argon. The reaction mixture was then pumped into the 3D printed flow reactor at a rate of 0.1 mL/min. The flow reactor was irradiated with a blue LED Kessil lamp (h*v* = 440 nm) under compressed air flow to maintain rt. The reaction mixture was collected in a round bottom flask and concentrated under vacuum. The crude residue was then diluted with DCM and washed with water, a saturated aqueous solution of NaHCO$_3$ and a saturated aqueous solution of brine, dried over anhydrous MgSO$_4$ and filtered. The filtrate was concentrated *in vacuo*. The crude product was finally purified by flash column chromatography over silica gel. [**Note:** Control experiments run without using any photocatalyst or in the absence of light showed no conversion]

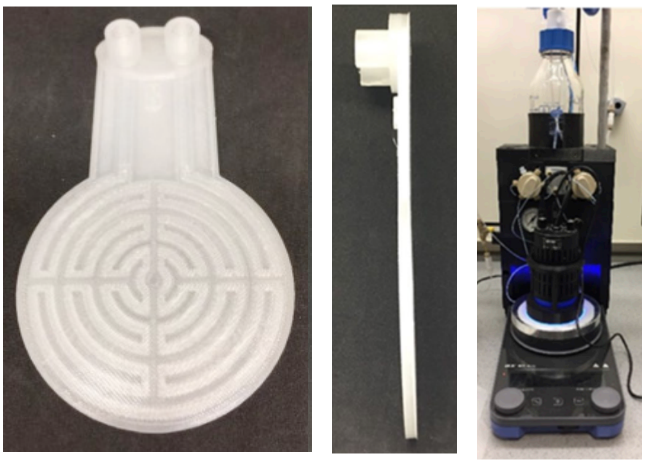

C.2 mmol scale reaction

fac-Ir(ppy)$_3$ (13 mg, 2 mol%), (difluoromethyl)triphenylphosphonium bromide (**dFM$_2$**) (785 mg, 2 mmol, 2.0 equiv.), **1a** (1 mmol, 1.0 equiv.), DCM/MeCN (1:1, 10 mL in total) and 2,6-lutidine (115 µL, 1 mmol, 1.0 equiv.) were added into a microwave tube under argon. The mixture was degassed by three freeze-pump-thaw cycles and backfilled with argon. The reaction mixture was then pumped into the 3D printed flow reactor at a rate of 0.1 mL/min. The flow reactor was irradiated with a blue LED Kessil lamp (hν = 440 nm) under compressed air flow to maintain rt. The reaction mixture was collected in a round bottom flask and concentrated under vacuum. The crude residue was then diluted with DCM and washed with water, a saturated aqueous solution of $NaHCO_3$ and a saturated aqueous solution of brine, dried over anhydrous $MgSO_4$ and filtered. The filtrate was concentrated *in vacuo*. The crude product was finally purified by flash column chromatography over silica gel.

C.3 Optimization reactions

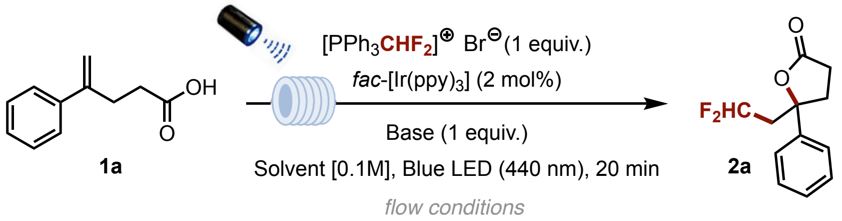

Entry	Base	Solvent	Yield[a]
1	2,6-Lutidine	DMF	50%
2	2,6-Lutidine	MeCN	71%
3	2,6-Di-*tert*-butylpyridine	MeCN	50%
4	2,6-Lutidine	CH_2Cl_2/MeCN	82%[b]

C.4 Characterization Data

5-(2,2-Difluoroethyl)-5-phenyldihydrofuran-2(3*H*)-one (2a)

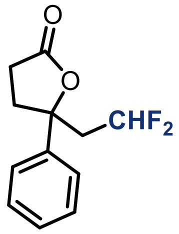

Molecular formula: $C_{12}H_{12}F_2O_2$ **MW =** 226.22 g.mol^{-1}

5-(2,2-Difluoroethyl)-5-phenyldihydrofuran-2(3*H*)-one was prepared following the general procedure. Purification by flash column chromatography over silica gel and eluting with hexane/EtOAc (4:1) afforded the desired product as a colourless oil (42 mg, 92%). A comparative experiment run in batch under otherwise identical conditions afforded the product in only 36% yield.

^{1}H NMR (400 MHz, CDCl$_3$) δ 7.46-7.30 (m, 5H), 5.71 (tt, *J* = 55.6, 4.7 Hz, 1H), 2.69-2.39 (m, 6H).

^{13}C NMR (101 MHz, CDCl$_3$) δ 175.7, 141.4, 129.1, 128.6, 124.6, 114.8 (t, *J* = 239.6 Hz), 85.4 (t, *J* = 6.0 Hz), 46.1 (t, *J* = 21.8 Hz), 35.1, 28.2.

^{19}F NMR (376.5 MHz, CDCl$_3$) δ –113.9 (m).

IR (neat, cm^{-1}) 2927, 1777, 1408, 1193, 1089, 1164, 1029, 937, 905, 843, 770.

HRMS (ESI) *m/z* Calcd. for $C_{12}H_{13}F_2O_2$ [M+H]$^+$: 227.0878; found: 227.0882.

5-(2,2-Difluoroethyl)-5-(*p*-tolyl)dihydrofuran-2(3*H*)-one (2b)

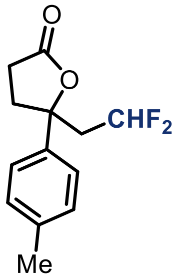

Molecular formula: $C_{13}H_{14}F_2O_2$ **MW =** 240.25 g.mol^{-1}

5-(2,2-Difluoroethyl)-5-(*p*-tolyl)dihydrofuran-2(3*H*)-one was prepared following the general procedure. Purification by flash column chromatography over silica gel and eluting with hexane/EtOAc (4:1) afforded the desired product as a colourless oil (36 mg, 75%).

^{1}H NMR (400 MHz, CDCl$_3$) δ 7.24 (dt, J = 18.2, 5.0 Hz, 4H), 5.69 (tt, J = 55.6, 4.7 Hz, 1H), 2.62-2.41 (m, 6H), 2.36 (s, 3H).

^{13}C NMR (101 MHz, CDCl$_3$) δ 175.8, 138.4, 138.3, 129.8, 124.6, 115.0 (t, J = 239.5 Hz), 85.4 (d, J = 6.0 Hz), 46.2 (t, J = 21.7 Hz), 35.1, 28.2, 21.2.

^{19}F NMR (376.5 MHz, CDCl$_3$) δ –113.9 (m).

IR (neat, cm^{-1}) 2926, 1777, 1515, 1407, 1183, 1071, 1023, 937, 907, 822, 731.

HRMS (ESI) *m/z* Calcd. for $C_{13}H_{14}F_2O_2Na^+$ [M+Na]$^+$: 263.0854; found: 263.0856.

5-(2,2-Difluoroethyl)-5-(4-fluorophenyl)dihydrofuran-2(3*H*)-one (2c)

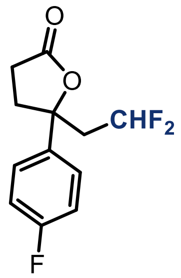

Molecular formula: $C_{12}H_{11}F_3O_2$ **MW =** 244.21 g.mol^{-1}

5-(2,2-Difluoroethyl)-5-(4-fluorophenyl)dihydrofuran-2(3*H*)-one was prepared following the general procedure. Purification by flash column chromatography over silica gel and eluting with hexane/EtOAc (2:1) afforded the desired product as a colourless oil (34 mg, 70%).

^{1}H NMR (400 MHz, CDCl$_3$) δ 7.40-7.31 (m, 2H), 7.16-7.05 (m, 2H), 5.71 (tt, J = 55.5, 4.7 Hz, 1H), 2.66-2.42 (m, 6H).

^{13}C NMR (101 MHz, CDCl$_3$) δ 175.4, 163.9, 161.4, 137.2 (d, J = 3.3 Hz), 126.6 (d, J = 8.2 Hz), 116.2, 116, 114.7 (t, J = 239.7 Hz), 85.0 (t, J = 5.9 Hz), 46.1 (t, J = 21.7 Hz), 35.1, 28.1.

^{19}F NMR (376.5 MHz, CDCl$_3$) δ –113.4 (dq, J = 8.4, 5.1 Hz), –113.9 (m).

IR (neat, cm^{-1}) 2970, 1777, 1604, 1511, 1368, 1227, 1190, 1068, 838, 660.

HRMS (ESI) *m/z* Calcd. for $C_{12}H_{11}F_3O_2Na$ [M+Na]$^+$: 267.0603; found: 267.0609.

5-(4-Chlorophenyl)-5-(2,2-difluoroethyl)dihydrofuran-2(3*H*)-one (2d)

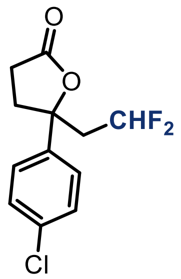

Molecular formula: $C_{12}H_{11}ClF_2O_2$ **MW =** 260.66 g.mol^{-1}

5-(4-Chlorophenyl)-5-(2,2-difluoroethyl)dihydrofuran-2(3*H*)-one was prepared following the general procedure. Purification by flash column chromatography over silica gel and eluting with hexane/EtOAc (2:1) afforded the desired product as a colourless oil (39 mg, 75%).

^{1}H NMR (400 MHz, CDCl$_3$) δ 7.39 (d, *J* = 8.8 Hz, 2H), 7.31 (d, *J* = 8.8 Hz, 2H), 5.73 (tt, *J* = 55.5, 4.7 Hz, 1H), 2.67-2.38 (m, 6H).

^{13}C NMR (101 MHz, CDCl$_3$) δ 175.4, 139.9, 134.6, 129.3, 126.1, 114.6 (t, *J* = 239.8 Hz), 84.9 (t, *J* = 5.9 Hz), 45.9 (t, *J* = 21.8 Hz), 35.1, 28.0.

^{19}F NMR (376.5 MHz, CDCl$_3$) δ –113.9 (dddd, *J* = 31.3, 22.5, 16.3, 14.5 Hz).

IR (neat, cm^{-1}) 2993,1777, 1492, 1404, 1179, 1068, 1012, 936, 905, 832, 732.

HRMS (ESI) *m/z* Calcd. for $C_{12}H_{12}ClF_2O_2$ [M+H]$^+$: 261.0488; found: 261.0490.

5-(4-Bromophenyl)-5-(2,2-difluoroethyl)dihydrofuran-2(3*H*)-one (2e)

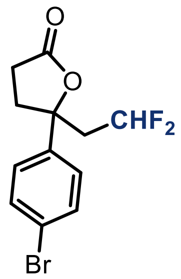

Molecular formula: $C_{12}H_{11}BrF_2O_2$ **MW =** 305.12 g.mol^{-1}

5-(4-Bromophenyl)-5-(2,2-difluoroethyl)dihydrofuran-2(3*H*)-one was prepared following the general procedure. Purification by flash column chromatography over silica gel and eluting with hexane/EtOAc (2:1) afforded the desired product as a colourless oil (44 mg, 72%).

^{1}H NMR (400 MHz, CDCl$_3$) δ 7.54 (d, J = 8.4 Hz, 2H), 7.25 (d, J = 8.5 Hz, 2H), 5.73 (tt, J = 55.5, 4.6 Hz, 1H), 2.66-2.42 (m, 6H).

^{13}C NMR (101 MHz, CDCl$_3$) δ 175.1, 140.4, 132.2, 126.3, 122.6, 114.5 (t, J = 239.8 Hz), 84.8 (t, J = 5.9 Hz), 45.8 (t, J = 21.8 Hz), 35.0, 27.9.

^{19}F NMR (376.5 MHz, CDCl$_3$) δ –113.8 (m).

IR (neat, cm^{-1}) 2983, 1778, 1488, 1398, 1193, 1072, 1009, 938, 907, 829.

HRMS (ESI) m/z Calcd. for $C_{12}H_{11}BrF_2O_2Na^+$ [M+Na]$^+$: 326.9803; found: 326.9806.

2'-(Difluoromethyl)-3,3',4,4'-tetrahydro-2'*H*,5*H*-spiro[furan-2,1'-naphthalen]-5-one (2f)

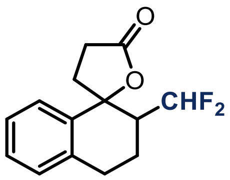

Molecular formula: $C_{14}H_{14}F_2O_2$ **MW =** 252.26 g.mol^{-1}

2'-(Difluoromethyl)-3,3',4,4'-tetrahydro-2'*H*,5*H*-spiro[furan-2,1'-naphthalen]-5-one was prepared following the general procedure. Purification by flash column chromatography over silica gel and eluting with hexane/EtOAc (4:1) afforded the desired product as a white solid (35 mg, 69%).

^{1}H NMR (400 MHz, CDCl$_3$) δ 7.29-7.24 (m, 3H), 7.14 (m, 1H), 6.03 (ddd, J = 56.6, 54.1, 2.3 Hz, 1H), 2.97 (dd, J = 8.7, 4.7 Hz, 2H), 2.85-2.53 (m, 4H), 2.26 (m, 2H), 1.91 (m, 1H).

^{13}C NMR (101 MHz, CDCl$_3$) δ 176.7, 140.3, 135, 129.2, 128.4, 127, 124.5, 115.7 (t, J = 241.4 Hz), 85.5 (d, J = 7.4 Hz), 46.7 (dd, J = 21.1, 19.5 Hz), 31.8 (d, J = 4.0 Hz), 28.6, 27.7, 17.9 (dd, J = 5.0, 3.1 Hz).

^{19}F NMR (376.5 MHz, CDCl$_3$) δ –122.5 (dddd, J = 292.3, 79.1, 55.5, 16.8 Hz).

IR (neat, cm^{-1}) 2960, 1771, 1492, 1401, 1245, 1209, 1162, 991, 911, 763.

HRMS (ESI) m/z Calcd. for $C_{14}H_{15}F_2O_2$ [M+H]$^+$: 253.1035; found: 253.1039.

5-(2,2-Difluoroethyl)-5-(phenylethynyl)dihydrofuran-2(3*H*)-one (2g)

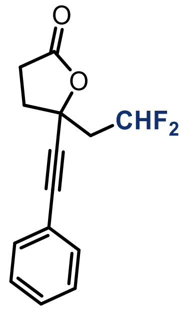

Molecular formula: $C_{14}H_{12}F_2O_2$ **MW =** 250.24 g.mol^{-1}

5-(2,2-Difluoroethyl)-5-(phenylethynyl)dihydrofuran-2(3*H*)-one was prepared following the general procedure. Purification by flash column chromatography over silica gel and eluting with hexane/EtOAc (4:1) afforded the desired product as a colourless oil (19 mg, 38%).

^{1}H NMR (400 MHz, $CDCl_3$) δ 7.52-7.32 (m, 5H), 6.19 (tdd, *J* = 55.7, 5.8, 3.6 Hz, 1H), 2.89 (ddd, *J* = 18.1, 10.8, 8.7 Hz, 1H), 2.71-2.36 (m, 5H).

^{13}C NMR (101 MHz, $CDCl_3$) δ 175.1, 132.0, 129.6, 128.6, 121.1, 115.0 (t, *J* = 239.8 Hz), 88.8, 85.4, 45.0 (t, *J* = 22.7 Hz), 36.2, 29.9, 28.7.

^{19}F NMR (376.5 MHz, $CDCl_3$) δ –114.0 (m).

IR (neat, cm^{-1}) 2926, 2234, 1783, 1491, 1173, 1069, 1027, 910, 757, 691.

HRMS (ESI) *m/z* Calcd. for $C_{14}H_{12}F_2O_2Na$ [M+Na]$^+$: 273.0698; found: 273.0706.

6-(2,2-Difluoroethyl)-6-phenyltetrahydro-2*H*-pyran-2-one (2h)

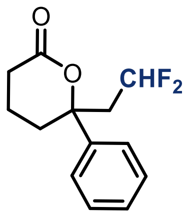

Molecular formula: $C_{13}H_{14}F_2O_2$ **MW =** 240.25 g.mol^{-1}

6-(2,2-Difluoroethyl)-6-phenyltetrahydro-2*H*-pyran-2-one was prepared following the general procedure. Purification by flash column chromatography over silica gel and eluting with hexane/EtOAc (3:1) afforded the desired product as a colourless oil (22 mg, 45%).

^{1}H NMR (400 MHz, CDCl$_3$) δ 7.40 (dd, J = 8.1, 1.0 Hz, 2H), 7.36-7.30 (m, 3H), 5.86 (tt, J = 55.7, 4.5 Hz, 1H), 2.56-2.36 (m, 5H), 2.16-2.07 (m, 1H), 1.83-1.73 (m, 1H).

^{13}C NMR (101 MHz, CDCl$_3$) δ 170.8, 141.7, 129.2, 128.2, 124.9, 114.84 (t, J = 238.9 Hz), 84.5 (t, J = 6.0 Hz), 47.8 (t, J = 21.7 Hz), 32.6, 29.1, 16.1.

^{19}F NMR (376.5 MHz, CDCl$_3$) δ –112.9 (m).

IR (neat, cm^{-1}) 2956, 1737, 1448, 1406, 1266, 1238, 1116, 1057, 936, 765, 733.

HRMS (ESI) *m/z* Calcd. for $C_{13}H_{15}F_2O_2$ [M+H]$^+$: 241.1035; found: 241.1035.

7-(2,2-Difluoroethyl)-7-phenyloxepan-2-one (2i)

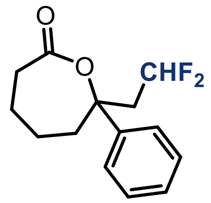

Molecular formula: $C_{14}H_{16}F_2O_2$ **MW =** 254.28 g.mol^{-1}

7-(2,2-Difluoroethyl)-7-phenyloxepan-2-one was prepared following the general procedure. Purification by flash column chromatography over silica gel and eluting with hexane/EtOAc (3:1) afforded the desired product as a colourless oil (6 mg, 11%).

^{1}H NMR (400 MHz, CDCl$_3$) δ 7.46-7.41 (m, 2H), 7.35 (dd, J = 7.8, 1.6 Hz, 1H), 7.32-7.28 (m, 2H), 5.94 (tt, J = 55.8, 4.5 Hz, 1H), 2.72 (d, J = 15.8 Hz, 1H), 2.62-2.53 (m, 1H), 2.35 (ddd, J = 19.0, 9.8, 4.0 Hz, 2H), 2.25-2.13 (m, 1H), 2.06-1.97 (m, 1H), 1.92-1.73 (m, 2H), 1.73-1.67 (m, 1H).

^{13}C NMR (101 MHz, CDCl$_3$) δ 174.8, 140.7, 129.5, 128.2, 125.5, 115.0 (t, J = 238.6 Hz), 83.9, 51.4, 37.2(d, J = 1.0 Hz), 37.1, 24.1, 23.0.

^{19}F NMR (376.5 MHz, CDCl$_3$) δ –112.1 (m).

HRMS (ESI) *m/z* Calcd. for $C_{14}H_{16}F_2O_2Na$ [M+Na]$^+$: 277.1011; found: 277.1023.

4-(Difluoromethyl)-5-phenyldihydrofuran-2(3*H*)-one (4a)

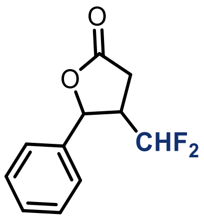

Molecular formula: $C_{11}H_{10}F_2O_2$ **MW =** 212.20 g.mol^{-1}

4-(Difluoromethyl)-5-phenyldihydrofuran-2(3*H*)-one was prepared following the general procedure. Purification by flash column chromatography over silica gel and eluting with hexane/EtOAc (2:1) afforded the desired product as a white solid (29 mg, 69%).

^{1}H NMR (400 MHz, CDCl$_3$) δ 7.48-7.36 (m, 3H), 7.35-7.30 (m, 2H), 5.95 (td, *J* = 55.5, 3.4 Hz, 1H), 5.52 (d, *J* = 5.7 Hz, 1H), 3.03-2.89 (m, 1H), 2.79 (qd, *J* = 18.0, 8.2 Hz, 2H).

^{13}C NMR (101 MHz, CDCl$_3$) δ 174.2, 138.0, 129.3, 129.2, 125.5, 114.9 (t, *J* = 243.1 Hz), 79.5 (dd, *J* = 4.9, 5.7 Hz), 47.5 (t, *J* = 21.4 Hz), 28.1 (t, *J* = 4.4 Hz).

^{19}F NMR (376.5 MHz, CDCl$_3$) δ –123.5 (m).

IR (neat, cm^{-1}) 2925, 1784, 1458,1113, 1170, 1050, 959, 763.

HRMS (ESI) *m/z* Calcd. for $C_{11}H_{10}F_2O_2Na$ [M+Na]$^+$: 235.0541; found: 235.0550.

(4*S*,5*S*)-4-(Difluoromethyl)-5-(naphthalen-2-yl)dihydrofuran-2(3*H*)-one (4b)

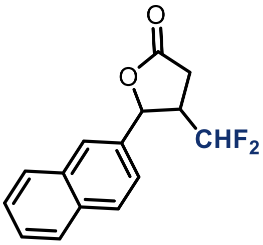

Molecular formula: $C_{15}H_{12}F_2O_2$ **MW =** 262.26 g.mol^{-1}

(4*S*,5*S*)-4-(Difluoromethyl)-5-(naphthalen-2-yl)dihydrofuran-2(3*H*)-one was prepared following the general procedure. Purification by flash column chromatography over silica gel and eluting with hexane/EtOAc (3:1) afforded the desired product as a white solid (25 mg, 48%).

^{1}H NMR (400 MHz, CDCl$_3$) δ 8.06-7.73 (m, 4H), 7.62-7.46 (m, 2H), 7.39 (dd, J = 8.6, 1.8 Hz, 1H), 6.00 (td, J = 55.5, 3.4 Hz, 1H), 5.70 (d, J = 5.5 Hz, 1H), 3.12-2.96 (m, 1H), 2.83 (ddd, J = 25.0, 18.1, 8.1 Hz, 2H).

^{13}C NMR (101 MHz, CDCl$_3$) δ 174.2, 135.3, 133.5, 133.2, 129.5, 128.3, 128.0, 127.1, 127.0, 124.9, 122.6, 115.0, 79.7, 47.5, 28.1.

^{19}F NMR (376.5 MHz, CDCl$_3$) δ –123.4 (m).

IR (neat, cm^{-1}) 2929, 1781, 1509, 1126, 1049, 952, 817, 748.

HRMS (ESI) *m/z* Calcd. for $C_{15}H_{13}F_2O_2$ [M+H]$^+$: 263.0878; found: 263.0882.

M.P. 62-63 °C

5-(4-Bromophenyl)-4-(difluoromethyl)dihydrofuran-2(3H)-one (4c)

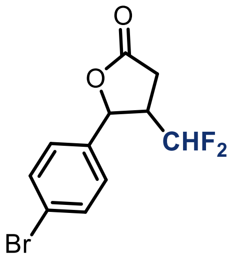

Molecular formula: $C_{11}H_9BrF_2O_2$ **MW =** 291.09 g.mol^{-1}

5-(4-Bromophenyl)-4-(difluoromethyl)dihydrofuran-2(3H)-one was prepared following the general procedure. Purification by flash column chromatography over silica gel and eluting with hexane/EtOAc (4:1) afforded the desired product as a brown powder (22 mg, 37%).

^{1}H NMR (400 MHz, CDCl$_3$) δ 7.48 (dd, J = 8.4, 2.7 Hz, 2H), 7.14 (dd, J = 8.3, 2.5 Hz, 2H), 6.06-5.70 (m, 1H), 5.49-5.35 (m, 1H), 2.93-2.59 (m, 3H).

^{13}C NMR (101 MHz, CDCl$_3$) δ 173.7, 137.1, 132.4 (two carbon signals are overlapping), 127.1 (two carbon signals are overlapping), 123.3, 114.8 (t, J = 243.2 Hz), 78.7 (t, J = 5.1 Hz), 47.4 (t, J = 21.3 Hz), 28.1 (t, J = 4.4 Hz).

^{19}F NMR (376.5 MHz, CDCl$_3$) δ –123.2 (m).

IR (neat, cm^{-1}) 2930, 1787, 1490, 1169, 1053, 1010, 952, 819.

HRMS (ESI) *m/z* Calcd. for $C_{11}H_{10}BrF_2O_2$ [M+H]$^+$: 290.9827; found: 290.9833.

5-(Benzo[d][1,3]dioxol-5-yl)-4-(difluoromethyl)dihydrofuran-2(3*H*)-one (4d)

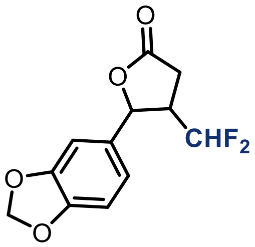

Molecular formula: $C_{12}H_{10}F_2O_4$ **MW =** 256.20 g.mol^{-1}

5-(Benzo[d][1,3]dioxol-5-yl)-4-(difluoromethyl)dihydrofuran-2(3*H*)-one was prepared following the general procedure. Purification by flash column chromatography over silica gel and eluting with hexane/EtOAc (2:1) afforded the desired product as a colourless oil (12 mg, 24%).

^{1}H NMR (400 MHz, CDCl$_3$) δ 6.84-6.77 (m, 3H), 6.00 (s, 2H), 5.92 (td, *J* = 55.5, 3.3 Hz, 1H), 5.40 (d, *J* = 6.1 Hz, 1H), 3.00-2.88 (m, 1H), 2.87-2.67 (m, 2H).

^{13}C NMR (101 MHz, CDCl$_3$) δ 174.0, 148.6, 148.5, 131.6, 119.6, 114.8 (t, *J* = 243.0 Hz), 108.7, 106.0, 101.7, 79.7 (dd, *J* = 6.1, 4.5 Hz), 47.6 (t, *J* = 21.3 Hz), 28.3 (t, *J* = 4.4 Hz).

^{19}F NMR (376.5 MHz, CDCl$_3$) δ –123.5 (m).

IR (neat, cm^{-1}) 2917, 1778, 1611, 1505, 1448, 1252, 1105, 1033, 926, 861, 795.

HRMS (ESI) *m/z* Calcd. for $C_{12}H_{10}F_2O_4Na$ [M+Na]$^+$: 279.0439; found: 279.0442.

4-(Difluoromethyl)-5-methyl-5-phenyldihydrofuran-2(3*H*)-one (4e)

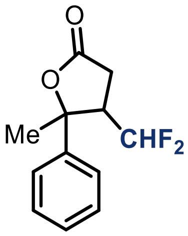

Molecular formula: $C_{12}H_{12}F_2O_2$ **MW =** 226.22 g.mol^{-1}

4-(Difluoromethyl)-5-methyl-5-phenyldihydrofuran-2(3*H*)-one was prepared following the general procedure. Purification by flash column chromatography over silica gel and eluting with hexane/EtOAc (4:1) afforded the desired product as a white solid (19 mg, 43%).

^{1}H NMR (400 MHz, $CDCl_3$) δ 7.44-7.31 (m, 5H), 6.10 (td, 1H), 3.12-2.99 (m, 1H), 2.70 (qd, *J* = 18.1, 7.2 Hz, 2H), 1.78 (t, 3H).

^{13}C NMR (101 MHz, $CDCl_3$) δ 173.8, 143.8, 129.1, 128.4, 124.2, 115.2, 86.1, 49.4, 28.9 (t, *J* = 4.6 Hz), 24.2.

^{19}F NMR (376.5 MHz, $CDCl_3$) δ –120.8 (dddd, *J* = 291.7, 73.7, 54.9, 14.6 Hz).

IR (neat, cm^{-1}) 2990, 1775, 1448, 1385, 1224, 1028, 952, 871, 806, 764.

HRMS (ESI) *m/z* Calcd. for $C_{12}H_{13}F_2O_2$ $[M+H]^+$: 227.0878; found: 227.0885.

2-(2,2-Difluoroethyl)-2-phenyltetrahydrofuran (6a)

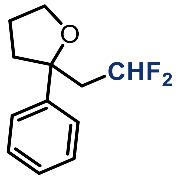

Molecular formula: $C_{12}H_{14}F_2O$ **MW =** 212.24 g.mol^{-1}

2-(2,2-Difluoroethyl)-2-phenyltetrahydrofuran was prepared following the general procedure. Purification by flash column chromatography over silica gel and eluting with hexane/EtOAc (12:1) afforded the desired product as a colourless oil (24 mg, 56%).

^{1}H NMR (400 MHz, $CDCl_3$) δ 7.39-7.21 (m, 4H), 7.21-7.09 (m, 1H), 5.65 (tt, *J* = 56.3, 4.6 Hz, 1H), 4.01-3.90 (m, 1H), 3.89-3.78 (m, 1H), 2.40-2.23 (m, 2H), 2.24-2.13 (m, 1H), 2.09 (dt, *J* = 12.4, 8.1 Hz, 1H), 1.95-1.80 (m, 1H), 1.71 (dqd, *J* = 12.3, 8.0, 6.8 Hz, 1H).

^{13}C NMR (101 MHz, $CDCl_3$) δ 145.4, 128.5, 127.1, 125.1, 116.2 (t, *J* = 238.3 Hz), 83.6 (t, *J* = 6.0 Hz), 67.9, 46.3 (t, *J* = 20.4 Hz), 38.6, 25.4.

^{19}F NMR (376.5 MHz, $CDCl_3$) δ –113.1 (m).

IR (neat, cm^{-1}) 2923, 2853, 1729, 1466, 1379, 1263.

HRMS (ESI) *m/z* Calcd. for $C_{12}H_{15}F_2O$ $[M+H]^+$: 213.1085; found: 213.1089.

2-(2,2-Difluoroethyl)-2-[4-(methylthio)phenyl]tetrahydrofuran (6b)

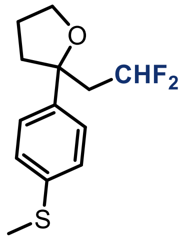

Molecular formula: $C_{13}H_{16}F_2OS$ **MW =** 258.33 g.mol^{-1}

2-(2,2-Difluoroethyl)-2-[4-(methylthio)phenyl]tetrahydrofuran was prepared following the general procedure. Purification by flash column chromatography over silica gel and eluting with hexane/EtOAc (10:1) afforded the desired product as a colourless oil (43 mg, 84%).

^{1}H NMR (400 MHz, CDCl$_3$) δ 7.24-7.19 (m, 2H), 7.17-7.12 (m, 2H), 5.81-5.46 (m, 1H), 3.92 (dd, *J* = 14.9, 7.6 Hz, 1H), 3.81 (td, *J* = 8.1, 5.6 Hz, 1H), 2.40 (s, 3H), 2.33-2.21 (m, 2H), 2.18-2.01 (m, 2H), 1.93-1.81 (m, 1H), 1.78-1.62 (m, 1H).

^{13}C NMR (101 MHz, CDCl$_3$) δ 142.2, 137.2, 126.7, 125.7 (d, *J* = 21.5 Hz), 116.1 (t, *J* = 238.4 Hz), 83.3 (t, *J* = 5.9 Hz), 67.9, 46.1 (t, *J* = 20.4 Hz), 38.5, 25.3, 15.9.

^{19}F NMR (376.5 MHz, CDCl$_3$) δ –113.3 (m).

IR (neat, cm^{-1}) 2923, 1397, 1188, 1024, 818, 731.

HRMS (ESI) *m/z* Calcd. for $C_{13}H_{17}F_2OS$ [M+H]$^+$: 259.0963; found: 259.0970.

2-(2,2-Difluoroethyl)-2-(3-methoxyphenyl)tetrahydrofuran (6c)

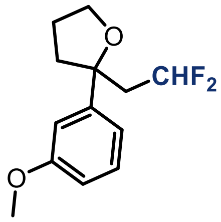

Molecular formula: $C_{13}H_{16}F_2O_2$ **MW =** 242.27 g.mol^{-1}

2-(2,2-Difluoroethyl)-2-(3-methoxyphenyl)tetrahydrofuran was prepared following the general procedure. Purification by flash column chromatography over silica gel and eluting with hexane/EtOAc (10:1) afforded the desired product as a colourless oil (27 mg, 56%).

^{1}H NMR (400 MHz, CDCl$_3$) δ 7.26 (dd, *J* = 8.8, 7.1 Hz, 1H), 6.99-6.94 (m, 1H), 6.94-6.88 (m, 1H), 6.82-6.75 (m, 1H), 5.72 (tt, *J* = 56.4, 4.6 Hz, 1H), 4.01 (dd, *J* = 14.9, 7.6 Hz, 1H), 3.91 (td, *J* = 8.1,

5.7 Hz, 1H), 3.81 (s, 3H), 2.43-2.29 (m, 2H), 2.29-2.20 (m, 1H), 2.14 (dt, *J* = 12.4, 8.1 Hz, 1H), 2.01-1.88 (m, 1H), 1.85-1.72 (m, 1H).

^{13}C NMR (101 MHz, CDCl$_3$) δ 159.9, 147.2, 129.6, 117.5, 116.2 (t, *J* = 238.3 Hz), 112.2, 111.2, 83.6 (t, *J* = 6.0 Hz), 68.0, 55.4, 46.2 (t, *J* = 20.5 Hz), 38.6, 25.4.

^{19}F NMR (376.5 MHz, CDCl$_3$) δ –113.2 (m).

IR (neat, cm^{-1}) 2926, 1584, 1286, 1101, 1045, 791, 707.

HRMS (ESI) *m/z* Calcd. for $C_{13}H_{17}F_2O_2$ [M+H]$^+$: 243.1191; found: 243.1205.

2-(2,2-Difluoroethyl)-2-(4-fluorophenyl)tetrahydrofuran (6d)

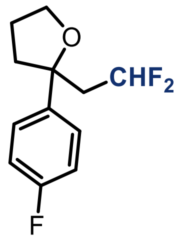

Molecular formula: $C_{12}H_{13}F_3O$ **MW =** 230.23 g.mol^{-1}

2-(2,2-Difluoroethyl)-2-(4-fluorophenyl)tetrahydrofuran was prepared following the general procedure. Purification by flash column chromatography over silica gel and eluting with hexane/EtOAc (12:1) afforded the desired product as a colourless oil (15 mg, 33%).

^{1}H NMR (400 MHz, CDCl$_3$) δ 7.38-7.30 (m, 2H), 7.07-6.98 (m, 2H), 5.90-5.55 (m, 1H), 4.01 (dt, *J* = 14.3, 7.1 Hz, 1H), 3.95-3.85 (m, 1H), 2.35 (dddd, *J* = 16.6, 15.4, 4.6, 3.4 Hz, 2H), 2.26-2.11 (m, 2H), 2.03-1.89 (m, 1H), 1.86-1.71 (m, 1H).

^{13}C NMR (101 MHz, CDCl$_3$) δ 163.2, 160.7, 141.1 (d, *J* = 3.1 Hz), 126.8 (d, *J* = 8.0 Hz), 116.1 (t, *J* = 238.4 Hz), 115.4, 115.2, 83.3 (t, *J* = 5.9 Hz), 68.0, 46.3 (t, *J* = 20.4 Hz), 38.8, 25.4.

^{19}F NMR (376.5 MHz, CDCl$_3$) δ –113.2 (m), –116.2 (tt, *J* = 8.6, 5.3 Hz).

IR (neat, cm^{-1}) 2963, 1508, 1222, 1159, 1043, 834, 732.

HRMS (ESI) *m/z* Calcd. for $C_{12}H_{14}F_3O$ [M+H]$^+$: 231.0991; found: 231.0995.

2-(2,2-Difluoroethyl)-2-phenyltetrahydro-2*H*-pyran (6e)

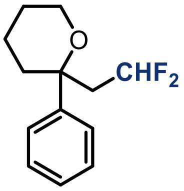

Molecular formula: $C_{13}H_{16}F_2O$ **MW =** 226.27 g.mol^{-1}

2-(2,2-Difluoroethyl)-2-phenyltetrahydro-2*H*-pyran was prepared following the general procedure. Purification by flash column chromatography over silica gel and eluting with hexane/EtOAc (12:1) afforded the desired product as a colourless oil (13 mg, 29%).

^{1}H NMR (400 MHz, CDCl$_3$) δ 7.45-7.36 (m, 4H), 7.33-7.26 (m, 1H), 5.93-5.41 (m, 1H), 3.79-3.70 (m, 1H), 3.61-3.47 (m, 1H), 2.39-2.30 (m, 1H), 2.30-2.14 (m, 2H), 1.91-1.81 (m, 1H), 1.75-1.58 (m, 2H), 1.55-1.37 (m, 2H).

^{13}C NMR (101 MHz, CDCl$_3$) δ 142.1, 128.9, 127.3, 126.6, 115.9 (t, *J* = 237.8 Hz), 75.8 (t, *J* = 6.2 Hz), 62.7, 48.8 (t, *J* = 20.5 Hz), 33.1, 25.9, 19.7.

^{19}F NMR (376.5 MHz, CDCl$_3$) δ –112.2 (m).

IR (neat, cm^{-1}) 2962, 1438, 1379, 1260, 1192, 1116, 1012, 800, 721.

HRMS (ESI) *m/z* Calcd. for $C_{13}H_{17}F_2O$ [M+H]$^+$: 227.1242; found: 227.1251.

3-(Difluoromethyl)-2-phenyltetrahydrofuran (8a)

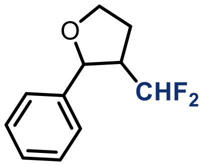

Molecular formula: $C_{11}H_{12}F_2O$ **MW =** 198.21 g.mol^{-1}

3-(Difluoromethyl)-2-phenyltetrahydrofuran was prepared following the general procedure. Purification by flash column chromatography over silica gel and eluting with hexane/EtOAc (10:1) afforded the desired product as a colourless oil (20 mg, 50%).

^{1}H NMR (400 MHz, CDCl$_3$) δ 7.38-7.33 (m, 4H), 7.33-7.27 (m, 1H), 5.87 (td, *J* = 56.4, 4.5 Hz, 1H), 4.92 (d, *J* = 6.1 Hz, 1H), 4.16 (td, *J* = 8.1, 5.5 Hz, 1H), 3.98 (dd, *J* = 15.8, 7.6 Hz, 1H), 2.72-2.56 (m, 1H), 2.27-2.17 (m, 1H), 2.14-2.05 (m, 1H).

^{13}C NMR (101 MHz, CDCl$_3$) δ 141.5, 128.7, 127.9, 125.9, 116.9 (t, J = 241.7 Hz), 80.3 (t, J = 4.9 Hz), 68.2, 51.4 (t, J = 19.8 Hz), 26.6 (t, J = 3.8 Hz).

^{19}F NMR (376.5 MHz, CDCl$_3$) δ –120.3 (m).

IR (neat, cm^{-1}) 2959, 1495, 1456, 1387, 1144, 1089, 1024, 757, 726.

HRMS (ESI) *m/z* Calcd. for $C_{11}H_{13}F_2O$ [M+H]$^+$: 199.0929; found: 199.0932.

3-(Difluoromethyl)-2-(4-methoxyphenyl)tetrahydrofuran (8b)

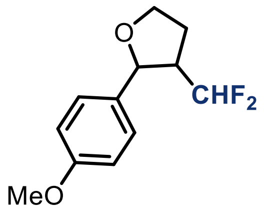

Molecular formula: $C_{12}H_{14}F_2O_2$ **MW =** 228.24 g.mol^{-1}

3-(Difluoromethyl)-2-(4-methoxyphenyl)tetrahydrofuran was prepared following the general procedure. Purification by flash column chromatography over silica gel and eluting with hexane/EtOAc (8:1) afforded the desired product as a colourless oil (231 mg, 67%).

^{1}H NMR (400 MHz, CDCl$_3$) δ 7.19 (dd, J = 5.8, 4.0 Hz, 2H), 6.94-6.70 (m, 2H), 5.76 (td, J = 56.4, 4.4 Hz, 1H), 4.76 (d, J = 6.5 Hz, 1H), 4.06 (td, J = 8.1, 5.5 Hz, 1H), 3.87 (dd, J = 15.8, 7.6 Hz, 1H), 3.73 (s, 3H), 2.74-2.36 (m, 1H), 2.13 (ddt, J = 12.9, 9.1, 7.5 Hz, 1H), 2.02 (ddt, J = 13.0, 7.6, 5.8 Hz, 1H).

^{13}C NMR (101 MHz, CDCl$_3$) δ 159.4, 133.3, 127.4, 117.0 (t, J = 241.6 Hz), 114.1, 80.2 (t, J = 5.0 Hz), 68.0, 55.4, 51.2 (t, J = 19.6 Hz), 26.6 (t, J = 3.8 Hz).

^{19}F NMR (376.5 MHz, CDCl$_3$) δ –120.4 (dddd, J = 283.0, 72.3, 56.4, 14.8 Hz).

IR (neat, cm^{-1}) 2963, 1614, 1515, 1259, 1175, 1026, 800.

HRMS (ESI) *m/z* Calcd. for $C_{12}H_{15}F_2O_2$ [M+H]$^+$: 229.1035; found: 229.1035.

2-[4-(Chloromethyl)phenyl]-3-(difluoromethyl)tetrahydrofuran (8c)

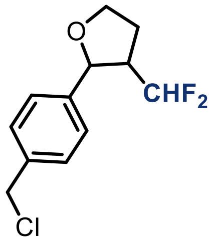

Molecular formula: $C_{12}H_{13}ClF_2O$ **MW =** 246.68 g.mol^{-1}

2-[4-(Chloromethyl)phenyl]-3-(difluoromethyl)tetrahydrofuran was prepared following the general procedure. Purification by flash column chromatography over silica gel and eluting with hexane/EtOAc (8:1) afforded the desired product as a colourless oil (26 mg, 53%).

^{1}H NMR (400 MHz, CDCl$_3$) δ 7.37 (dd, *J* = 15.4, 8.1 Hz, 4H), 6.05-5.70 (m, 1H), 4.93 (d, *J* = 6.0 Hz, 1H), 4.60 (d, *J* = 14.4 Hz, 2H), 4.14 (dd, *J* = 13.7, 8.0 Hz, 1H), 3.98 (dd, *J* = 15.5, 7.6 Hz, 1H), 2.69-2.55 (m, 1H), 2.19 (dt, *J* = 16.4, 7.8 Hz, 1H), 2.09 (td, *J* = 12.9, 5.7 Hz, 1H).

^{13}C NMR (101 MHz, CDCl$_3$) δ 142.0, 137.2, 129.5, 129.0, 126.4, 126.3, 116.9 (t, *J* = 243.2 Hz), 79.9 (t, *J* = 4.8 Hz), 68.2, 51.4 (t, *J* = 19.9 Hz), 46.0, 26.7 (t, *J* = 3.8 Hz).

^{19}F NMR (376.5 MHz, CDCl$_3$) δ –120.2 (m).

IR (neat, cm^{-1}) 2962, 1719, 1387, 1264, 1243, 1066, 796.

HRMS (ESI) *m/z* Calcd. for $C_{12}H_{14}ClF_2O$ [M+H]$^+$: 247.0696; found: 247.0698.

3-(Difluoromethyl)-2-(4-fluorophenyl)tetrahydrofuran (8d)

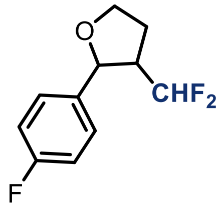

Molecular formula: $C_{11}H_{11}F_3O$ **MW =** 216.20 g.mol^{-1}

3-(Difluoromethyl)-2-(4-fluorophenyl)tetrahydrofuran was prepared following the general procedure. Purification by flash column chromatography over silica gel and eluting with hexane/EtOAc (10:1) afforded the desired product as a colourless oil (13 mg, 29%).

^{1}H NMR (400 MHz, CDCl$_3$) δ 7.36-7.30 (m, 2H), 7.08-7.01 (m, 2H), 5.86 (td, J = 56.3, 4.5 Hz, 1H), 4.89 (d, J = 6.3 Hz, 1H), 4.14 (td, J = 8.2, 5.4 Hz, 1H), 4.01-3.90 (m, 1H), 2.70-2.51 (m, 1H), 2.26-2.15 (m, 1H), 2.09 (ddt, J = 13.0, 7.5, 5.6 Hz, 1H).

^{13}C NMR (101 MHz, CDCl$_3$) δ 163.7, 161.3, 137.2 (d, J = 3.1 Hz), 127.7 (d, J = 8.1 Hz), 116.9 (t, J = 241.7 Hz), 115.7, 115.5, 79.75 (t, J = 4.8 Hz), 68.1, 51.44 (t, J = 19.7 Hz), 26.72 (t, J = 3.9 Hz).

^{19}F NMR (377 MHz, CDCl$_3$) δ –114.7 (tt, J = 8.6, 5.3 Hz), –120.1 (m).

IR (neat, cm^{-1}) 2963, 1720, 1605, 1509, 1144, 1068, 799.

HRMS (ESI) *m/z* Calcd. for $C_{11}H_{12}F_3O$ $[M+H]^+$: 217.0835; found: 217.0839.

4-(3-(Difluoromethyl)tetrahydrofuran-2-yl)phenyl acetate (8e)

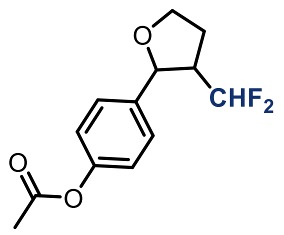

Molecular formula: $C_{13}H_{14}F_2O_3$ **MW =** 256.25 g.mol^{-1}

4-[3-(Difluoromethyl)tetrahydrofuran-2-yl]phenyl acetate was prepared following the general procedure. Purification by flash column chromatography over silica gel and eluting with hexane/EtOAc (6:1) afforded the desired product as a colourless oil (30 mg, 58%).

^{1}H NMR (400 MHz, CDCl$_3$) δ 7.36 (d, J = 8.5 Hz, 2H), 7.08 (d, J = 8.6 Hz, 2H), 5.87 (td, J = 56.3, 4.5 Hz, 1H), 4.93 (d, J = 6.0 Hz, 1H), 4.13 (dd, J = 13.6, 8.1 Hz, 1H), 3.96 (dd, J = 15.7, 7.7 Hz, 1H), 2.70-2.55 (m, 1H), 2.30 (s, 3H), 2.24-2.14 (m, 1H), 2.08 (dq, J = 8.1, 5.9 Hz, 1H).

^{13}C NMR (101 MHz, CDCl$_3$) δ 169.6, 150.3, 139.2, 127.0, 121.8, 116.9 (t, J = 241.8 Hz), 79.7, 68.2, 51.4, 26.6, 21.3.

^{19}F NMR (377 MHz, CDCl$_3$) δ –120.3 (m).

IR (neat, cm^{-1}) 2963, 1753, 1194, 1015,798.

HRMS (ESI) *m/z* Calcd. for $C_{13}H_{15}F_2O_3$ $[M+H]^+$: 257.0984; found: 257.0988.

3-(2,2-Difluoroethyl)-3-phenylisobenzofuran-1(3*H*)-one (10a)

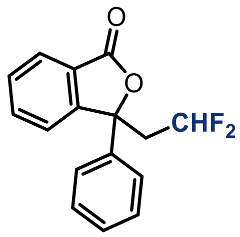

Molecular formula: $C_{16}H_{12}F_2O_2$ **MW =** 274.27 g.mol^{-1}

3-(2,2-Difluoroethyl)-3-phenylisobenzofuran-1(3*H*)-one was prepared following the general procedure. Purification by flash column chromatography over silica gel and eluting with hexane/EtOAc (4:1) afforded the desired product as an off-white solid (44 mg, 81%).

^{1}H NMR (400 MHz, CDCl$_3$) δ 7.92 (dt, *J* = 7.7, 0.9 Hz, 1H), 7.74-7.68 (m, 1H), 7.63-7.55 (m, 2H), 7.54-7.49 (m, 2H), 7.39 (ddd, *J* = 7.6, 4.7, 1.4 Hz, 2H), 7.36-7.30 (m, 1H), 5.95-5.61 (m, 1H), 3.09 (dddd, *J* = 19.3, 15.2, 11.9, 4.1 Hz, 1H), 2.81 (dddd, *J* = 15.9, 15.2, 12.5, 5.0 Hz, 1H).

^{13}C NMR (101 MHz, CDCl$_3$) δ 169.2, 151.7, 139.4, 134.8, 129.9, 129.2, 128.9, 126.4, 124.9, 124.7, 122.6, 114.5 (t, *J* = 240.4 Hz), 86.0 (dd, *J* = 6.9, 5.5 Hz), 44.2 (t, *J* = 22.5 Hz).

^{19}F NMR (376.5 MHz, CDCl$_3$) δ –113.2 (m).

IR (neat, cm^{-1}) 1753, 1466, 1288, 1232, 1118, 1058, 1037, 754, 698.

HRMS (ESI) *m/z* Calcd. for $C_{16}H_{12}F_2O_2Na$ $[M+Na]^+$: 297.0698; found: 297.0707.

M.P. 106-108 °C

3-(2,2-Difluoroethyl)-3-methylisobenzofuran-1(3*H*)-one (10b)

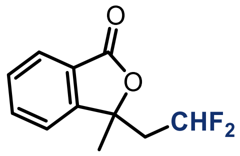

Molecular formula: $C_{11}H_{10}F_2O_2$ **MW =** 212.20 g.mol^{-1}

3-(2,2-Difluoroethyl)-3-methylisobenzofuran-1(3*H*)-one was prepared following the general procedure. Purification by flash column chromatography over silica gel and eluting with hexane/EtOAc (4:1) afforded the desired product as a white solid (12 mg, 28%).

^{1}H NMR (400 MHz, CDCl$_3$) *δ* 7.90 (dt, *J* = 7.7, 0.9 Hz, 1H), 7.71 (td, *J* = 7.6, 1.1 Hz, 1H), 7.56 (td, *J* = 7.6, 0.9 Hz, 1H), 7.43 (dt, *J* = 7.7, 0.8 Hz, 1H), 5.87 (tdd, *J* = 55.6, 5.1, 4.2 Hz, 1H), 2.61 (dddd, *J* = 16.1, 15.1, 13.7, 5.1 Hz, 1H), 2.39 (ddd, *J* = 19.1, 14.4, 4.2 Hz, 1H), 1.71 (s, 3H).

^{13}C NMR (101 MHz, CDCl$_3$) *δ* 169.1, 152.9, 134.7, 129.8, 126.3, 125.3, 121.2, 114.7 (t, *J* = 239.8 Hz), 83.7 (dd, *J* = 6.1, 5.6 Hz), 44.0 (t, *J* = 22.0 Hz), 26.6.

^{19}F NMR (376.5 MHz, CDCl$_3$) *δ* –113.3 (m).

IR (neat, cm^{-1}) 2921, 1745, 1290, 1226, 1129, 1079, 1019, 916, 763, 698.

HRMS (ESI) *m/z* Calcd. for $C_{11}H_{10}F_2O_2Na$ [M+Na]$^+$: 235.0541; found: 235.0549.

M.P. 64-66 °C

1-(2,2-Difluoroethyl)-1-phenyl-1,3-dihydroisobenzofuran (12)

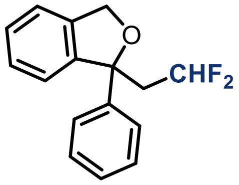

Molecular formula: $C_{16}H_{14}F_2O$ **MW =** 260.28 g.mol^{-1}

1-(2,2-Difluoroethyl)-1-phenyl-1,3-dihydroisobenzofuran was prepared following the general procedure. Purification by flash column chromatography over silica gel and eluting with hexane/EtOAc (6:1) afforded the desired product as a colourless oil (42 mg, 80%).

^{1}H NMR (400 MHz, CDCl$_3$) *δ* 7.46-7.38 (m, 2H), 7.32-7.12 (m, 7H), 5.74 (tdd, *J* = 56.2, 4.9, 4.0 Hz, 1H), 5.12 (q, *J* = 12.3 Hz, 2H), 2.81-2.58 (m, 2H).

^{13}C NMR (101 MHz, CDCl$_3$) *δ* 144.0, 142.9, 138.7, 128.7, 128.2, 127.8, 127.6, 125.3, 124.9, 122.3, 121.4, 115.9 (t, *J* = 239.1 Hz), 88.2 (dd, *J* = 7.0, 5.6 Hz), 72.2, 45.8 (t, *J* = 21.0 Hz).

^{19}F NMR (377 MHz, CDCl$_3$) *δ* –112.5 (m).

IR (neat, cm^{-1}) 2926, 1755, 1339, 1117, 1053, 752, 728.

HRMS (ESI) *m/z* Calcd. for $C_{16}H_{15}F_2O$ [M+H]$^+$: 261.1085; found: 261.1089.

1-[2-(2,2-Difluoroethyl)-2-phenylpyrrolidin-1-yl]ethan-1-one (14)

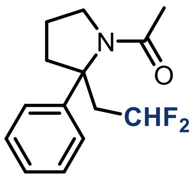

Molecular formula: $C_{14}H_{17}F_2NO$ **MW =** 253.29 g.mol^{-1}

1-[2-(2,2-Difluoroethyl)-2-phenylpyrrolidin-1-yl]ethan-1-one was prepared following the general procedure. Purification by flash column chromatography over silica gel and eluting with hexane/EtOAc (1:1) afforded the desired product as a colourless oil (18 mg, 36%).

^{1}H NMR (400 MHz, CDCl$_3$) δ 7.31 (dt, *J* = 9.9, 1.9 Hz, 1H), 7.24-7.19 (m, 1H), 7.11 (dt, *J* = 8.5, 1.8 Hz, 1H), 5.85 (dddd, *J* = 57.9, 55.3, 6.8, 2.7 Hz, 1H), 3.78 (ddd, *J* = 10.5, 8.1, 2.5 Hz, 1H), 3.66 (td, *J* = 10.0, 6.9 Hz, 1H), 3.25 (dddd, *J* = 29.5, 14.3, 11.4, 2.7 Hz, 1H), 2.73 (tdd, *J* = 14.2, 12.7, 6.8 Hz, 1H), 2.48 (td, *J* = 12.3, 6.8 Hz, 1H), 2.20 (s, 3H), 2.06 (ddt, *J* = 12.8, 4.6, 2.5 Hz, 1H), 1.89-1.79 (m, 1H), 1.79-1.64 (m, 1H).

^{13}C NMR (101 MHz, CDCl$_3$) δ 169.8, 145.0, 128.6, 127.0, 124.8, 117.1 (t, *J* = 238.7 Hz), 50.4, 41.2 (t, *J* = 20.3 Hz), 33.6, 29.8, 24.3, 21.7.

^{19}F NMR (376 MHz, CDCl$_3$) δ –112.8 (m)

IR (neat, cm^{-1}) 1647, 1400, 1113.

HRMS (ESI) *m/z* Calcd. for $C_{14}H_{18}F_2NO$ [M+H]$^+$: 254.1351; found: 254.1356.

***N*-(3,3-Difluoro-1-phenylpropyl)acetamide (17)**

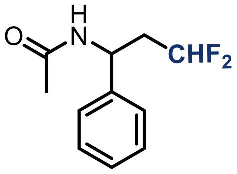

Molecular formula: $C_{11}H_{13}F_2NO$ **MW =** 213.23 g.mol^{-1}

N-(3,3-Difluoro-1-phenylpropyl)acetamide was prepared following the general procedure. Purification by flash column chromatography over silica gel and eluting with hexane/EtOAc (1:1) afforded the desired product as a colourless oil (24 mg, 55%).

^{1}H NMR (400 MHz, $CDCl_3$) δ 7.41-7.27 (m, 5H), 5.94 (s, 1H), 5.93-5.59 (tt, 1H), 5.25 (dd, *J* = 14.2, 8.1 Hz,1H), 2.52-2.25 (m, 2H), 1.99 (s, 3H).

^{13}C NMR (101 MHz, $CDCl_3$) δ 169.5, 140.3, 129.2, 128.2, 126.5, 116.0 (t, J = 239.4 Hz), 48.9 (t, J = 6.4 Hz), 40.5 (t, J = 21.1 Hz), 23.5.

^{19}F NMR (376 MHz, $CDCl_3$) δ –115.0 (m).

IR (neat, cm^{-1}) 3277, 3066, 1648, 1544, 1374, 1260, 1198, 1117, 1052, 1014, 968, 801, 761.

HRMS (ESI) *m/z* Calcd. for $C_{11}H_{14}F_2NO$ $[M+H]^+$: 214.1038; found: 214.1042.

D. Mechanistic investigation

D.1. Radical Trapping Experiment

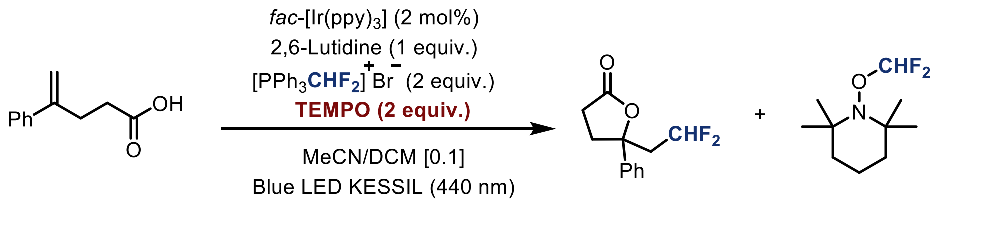

Entry	Condition	Yield of 2aa[a]	Yield of adduct[a]
1	In batch	50%	41%
2	In flow	75%	13%

[a]Yield determined by ^{19}F NMR using trifluorotoluene as internal standard.

In a flame-dried microwave tube under an argon atmosphere were dissolved *fac*-Ir(ppy)$_3$ (2.6 mg, 2 mol%), (difluoromethyl)triphenylphosphonium bromide **dFM₂** (157 mg, 0.4 mmol, 2.0 equiv.), 4-phenylpent-4-enoic acid **1a** (35 mg, 0.2 mmol, 1.0 equiv.), TEMPO (63 mg, 0.4 mmol, 2.0 equiv.) and 2,6-lutidine (23 µL, 0.2 mmol, 1.0 equiv.) in a 1:1 DCM/MeCN (2 mL) solution. The mixture was degassed by three consecutive freeze-pump-thaw cycles and backfilled with argon. The reaction run in batch was stirred for 2 h (**entry 1**), while the reaction run under continuous flow was injected into the 3D printed flow reactor at a 0.2 mL/min flow rate (irradiation time: 20 min, **entry 2**). Both reactions were irradiated with a blue LED Kessil lamp (h*v* = 440 nm) and the temperature was maintained constant by blowing compressed air onto the system. The yields were determined by ^{19}F NMR using benzotrifluoride as an internal standard. The reaction run in batch afforded the CHF_2-TEMPO adduct in 41% yield along with 50% of **2a**, while the reaction run under continuous flow led to the formation of the CHF_2-TEMPO adduct in 13% yield along with 75% of **2a**. The data for the CHF_2-TEMPO adduct were in accordance with the ones previously reported in the literature.[16] These results suggest that a difluoromethyl radical is indeed involved in the photocatalytic process.

D.2. Stern-Volmer Quenching Experiments

For this fluorescence quenching experiment, we used a freshly prepared and degassed $5{\times}10^{-5}$M solution of *fac*-Ir(ppy)$_3$ in DCM/MeCN (1:1) with the appropriate amount of quencher. The samples were transferred in screw-top quartz cuvettes at room temperature under argon and irradiated at 375 nm. Fluorescence was measured from 400 nm to 700 nm.

(a) (b)

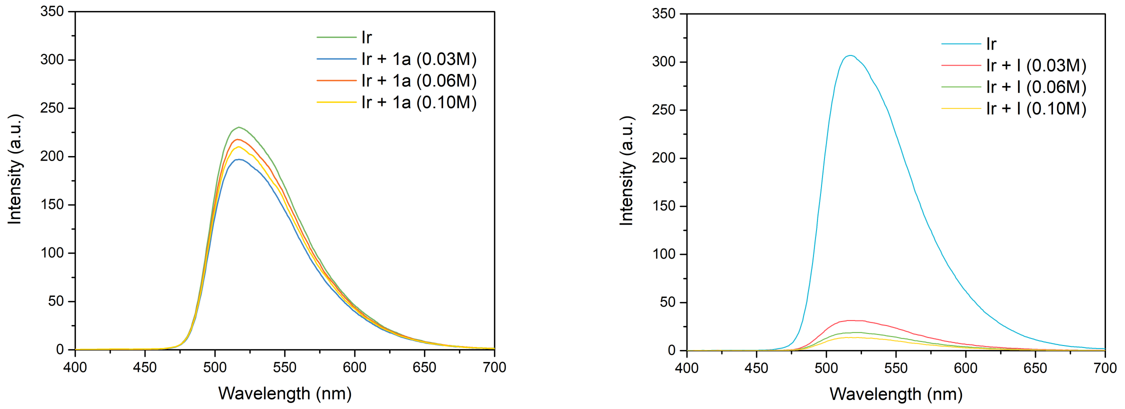

Figure S1. Luminescence quenching study: (a) the emission spectra of a $5{\times}10^{-5}$ M solution of *fac*- Ir(ppy)$_3$ with reagent **1a** in degassed acetone excited at 375 nm; (b) the emission spectra of a $5{\times}10^{-5}$ M solution of *fac*- Ir(ppy)$_3$ with various concentrations of **iii** in degassed acetone excited at 375 nm. **dFM1** is noted **I** in the diagram.

In these luminescence quenching experiments, it appeared that the difluoromethyl triphenylphosphonium bromide **dFM$_2$** (noted **I** in the diagram) is an efficient quencher for the [Ir]* while the substrate does not quench the [Ir]*. These results suggest that our *fac*-Ir(ppy)$_3$-mediated photocatalytic process proceeds through an oxidative quenching, which is consistent with the mechanism that we propose.

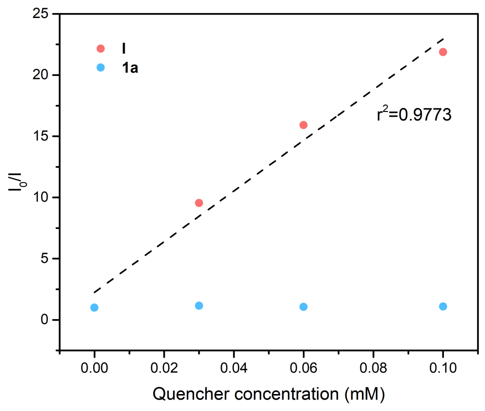

D.3. Computational details

General. DFT calculations were performed using the PBE0 functional[17] as implemented in the Gaussian 16 package,[18] including Grimme 3D dispersion corrections[19] and SMD solvation model.[20] Pople's 6-31G(d,p) basis set was used for all atoms.[21] Stationary points where confirmed by analysis of vibrational frequencies, calculated at the same level of theory. Energies reported in the manuscript are Gibbs free energies obtained from the frequencies calculations. Optimised geometries and absolute energies for the lowest energy conformation of each key structure are shown below. A dataset of original output files is available online [22] at the IOChem-BD repository, [23] including additional structures corresponding to the entire conformational search. The table below shows a summary of structures and file name definitions for the dataset.

Radical addition to styrene		
**Description**	**Structure**	**File naming scheme**
Styrene	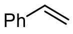	asty
CHF_2 radical	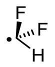	chf2
Radical addition transition state	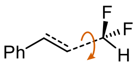	absty-ts1, -2, -3<br>Numbers denote rotamers around highlighted bond
Benzylic radical	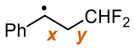	bsty-xy (x, y = a, b or c)<br>Letters denote rotation around corresponding bonds
Benzylic cation	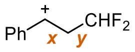	csty-xy (x, y = a, b or c)<br>Letters denote rotation around corresponding bonds
Acetoxylation product	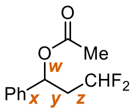	dsty-wxyz (w, x, y, z = a, b or c)<br>Letters denote rotation around corresponding bonds
Acetic acid	AcOH	acoh
Lutidine	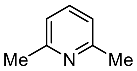	elut2
Lutidinium	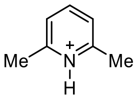	eluth2

**Model of intramolecular reaction**		
**Description**	**Structure**	**File naming scheme**
Benzylic radical<br>Conformations with *cis*<br>arrangement of Hs	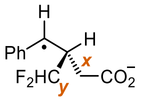	b5ldeprot-cis-xay<br>(x, y = a, b or c)
Benzylic radical<br>Conformations with *trans*<br>arrangement of Hs	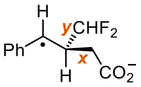	b5ldeprot-trans-xay<br>(x, y = a, b or c)

E. ^{1}H, ^{13}C and ^{19}F NMR Spectra

^{1}H-NMR of compound 2a (400 MHz, $CDCl_3$)

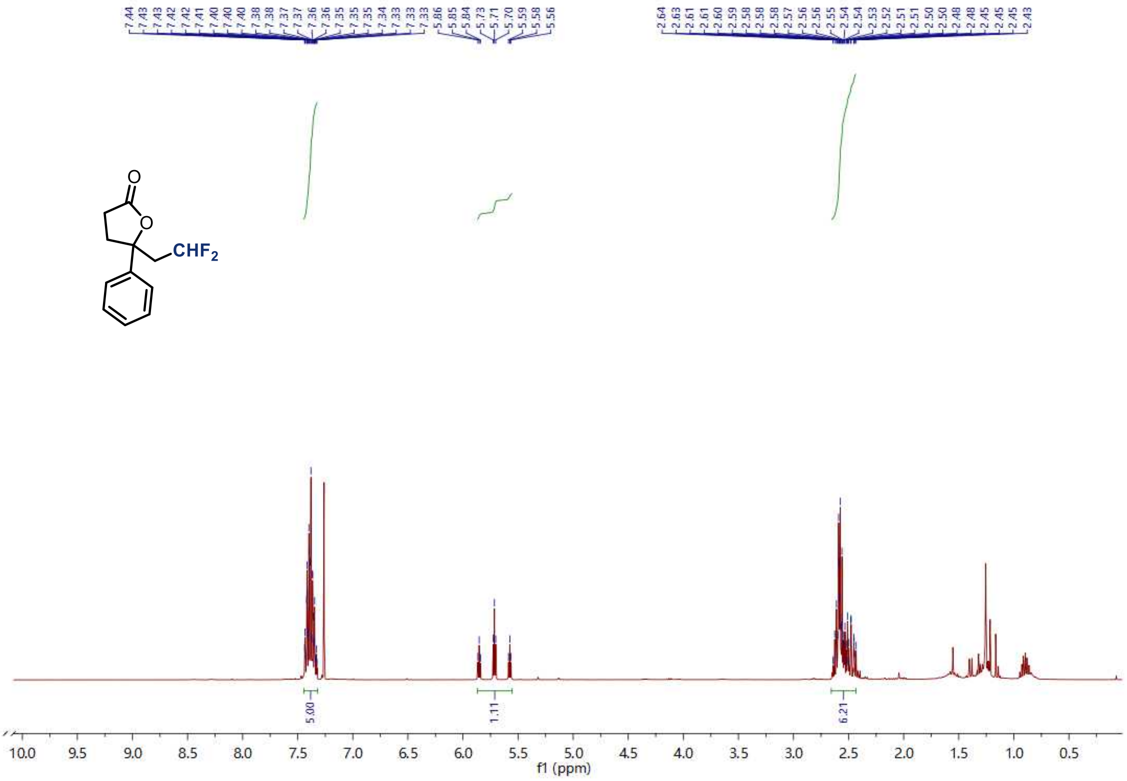

^{13}C-NMR of compound 2a (101 MHz, $CDCl_3$)

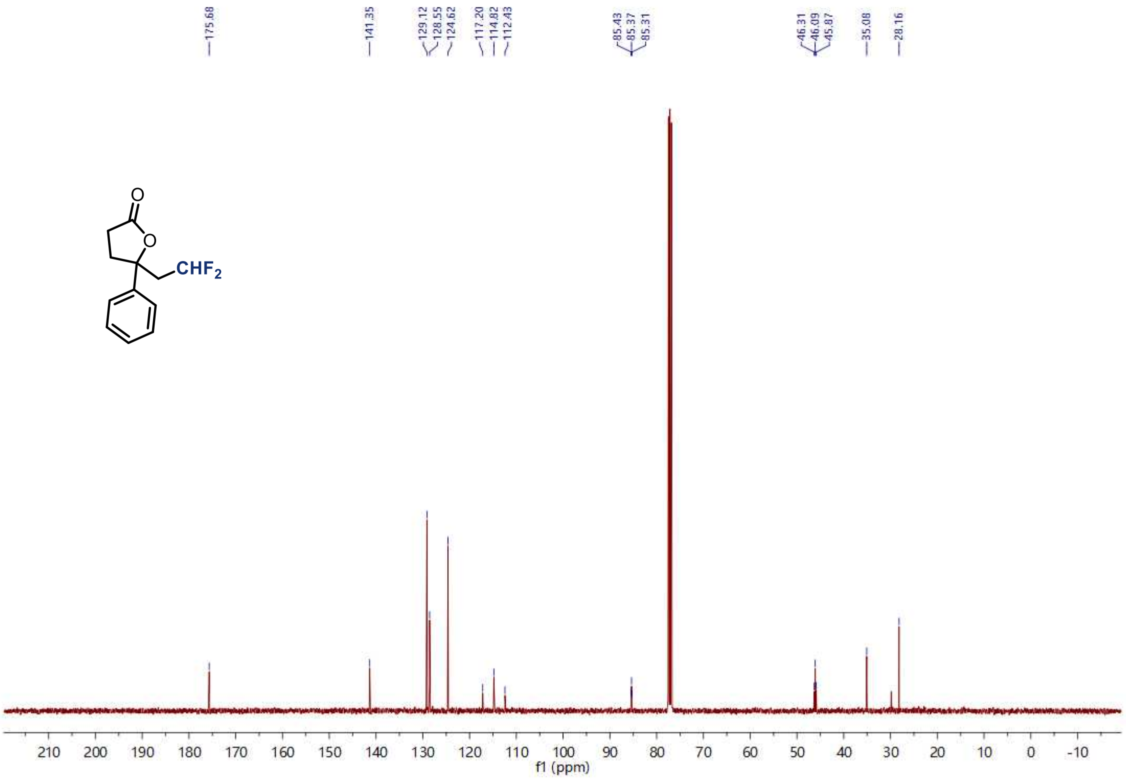

^{19}F-NMR of compound 2a (377 MHz, $CDCl_3$)

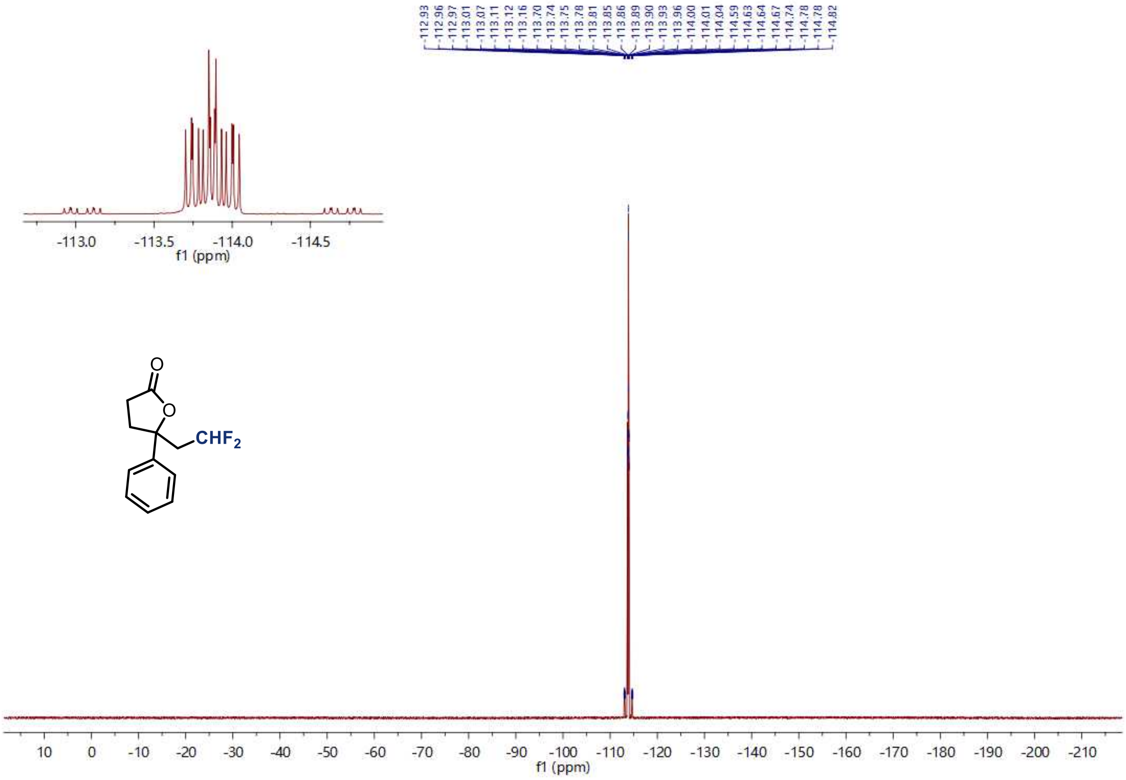

^{1}H-NMR of compound 2b (400 MHz, $CDCl_3$)

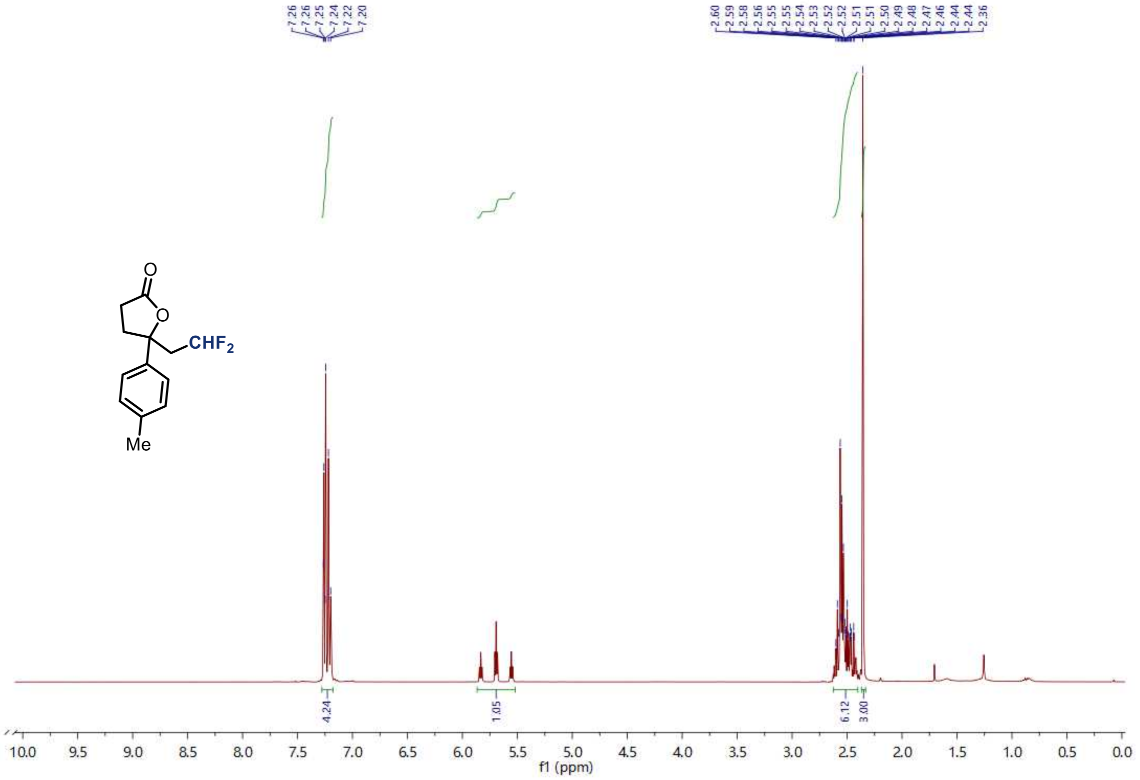

^{13}C-NMR of compound 2b (101 MHz, $CDCl_3$)

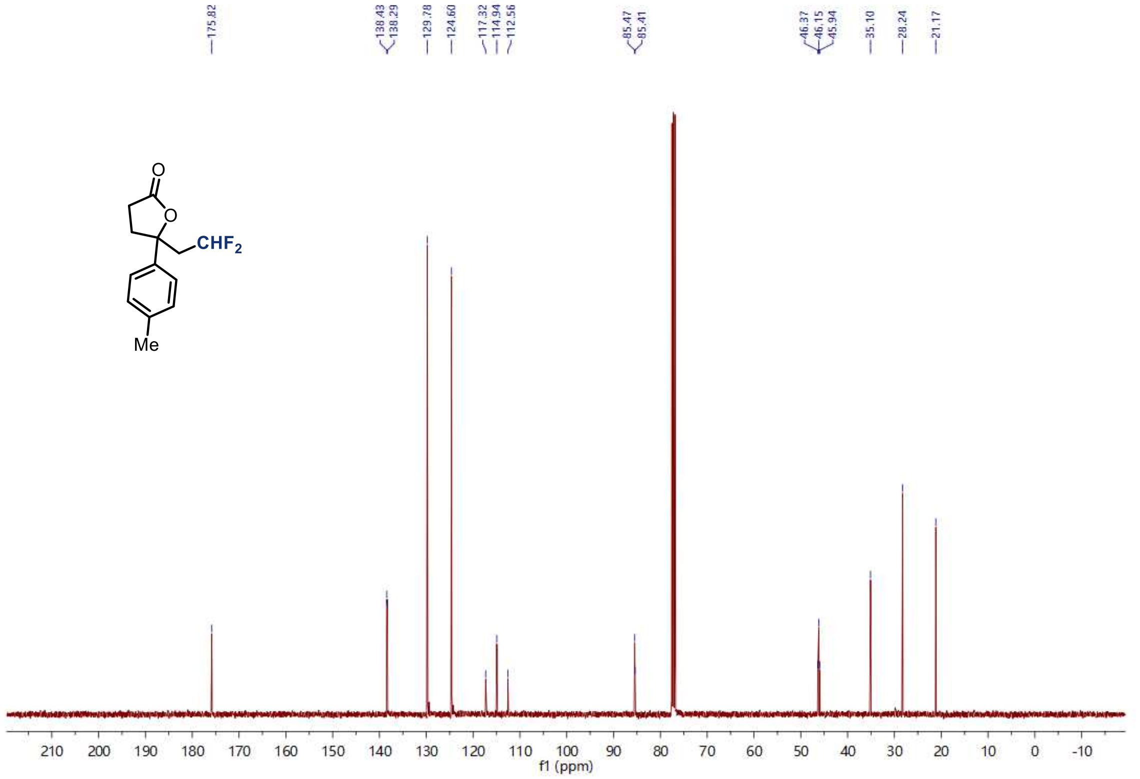

^{19}F-NMR of compound 2b (377 MHz, $CDCl_3$)

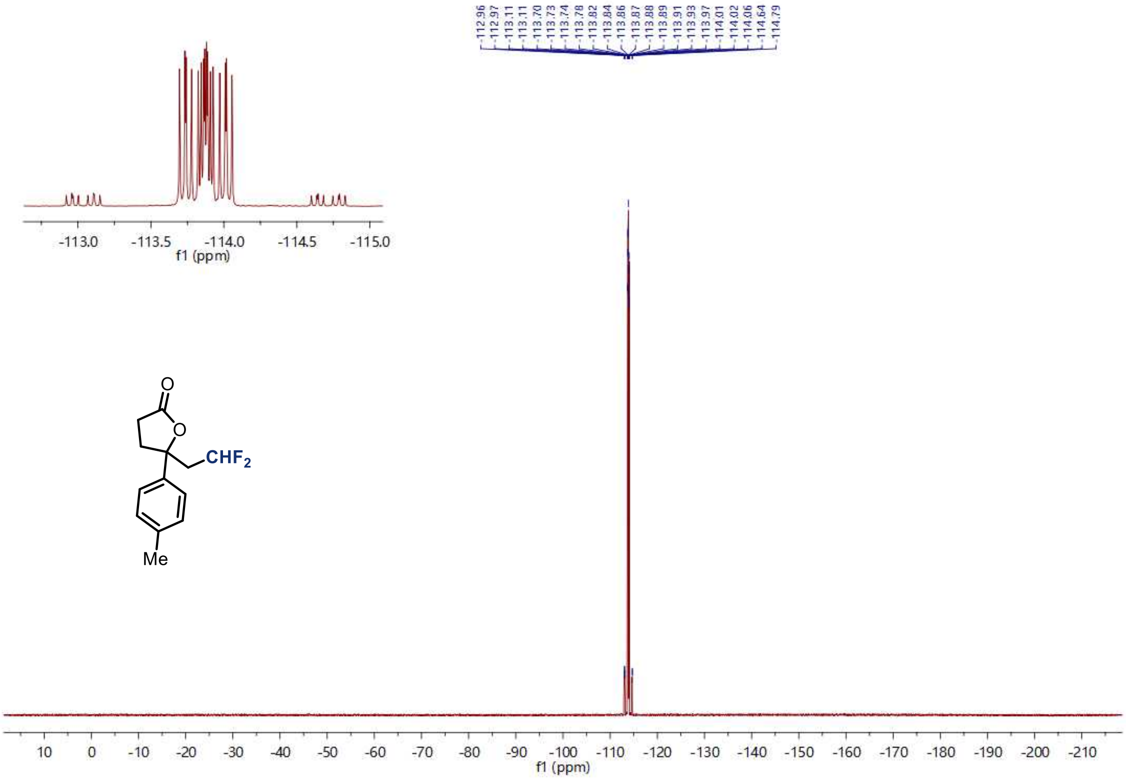

^{1}H-NMR of compound 2c (400 MHz, CDCl$_3$)

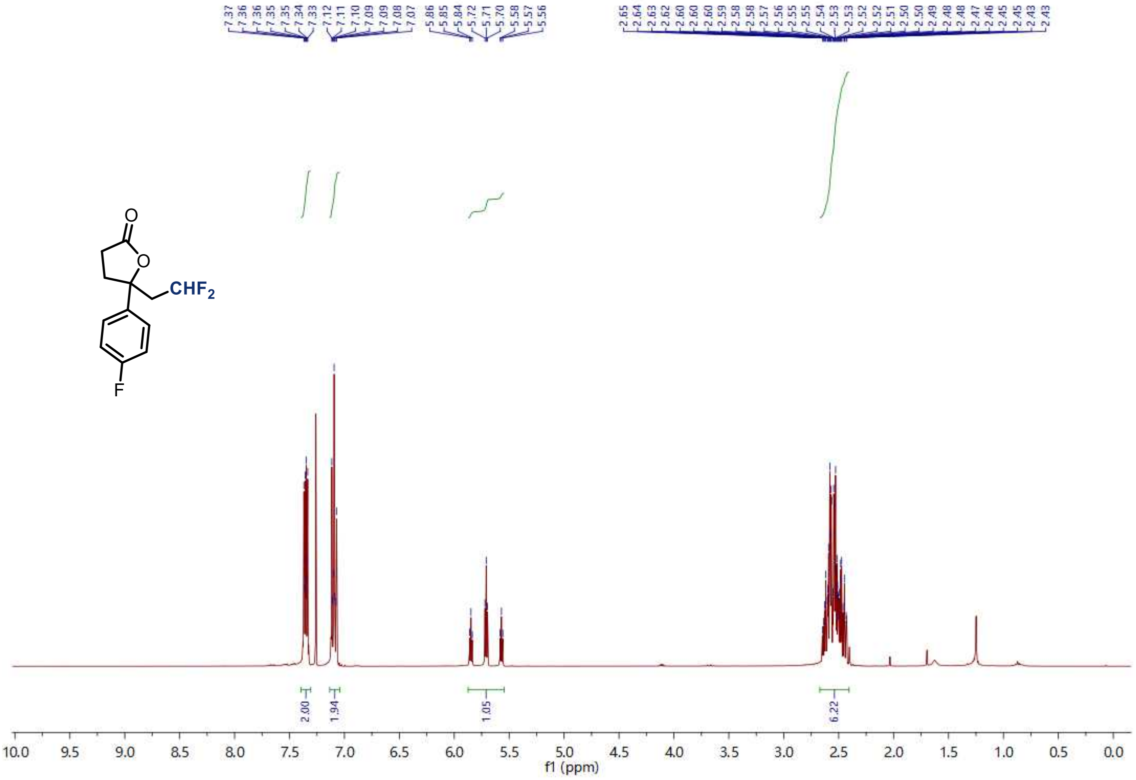

^{13}C-NMR of compound 2c (101 MHz, CDCl$_3$)

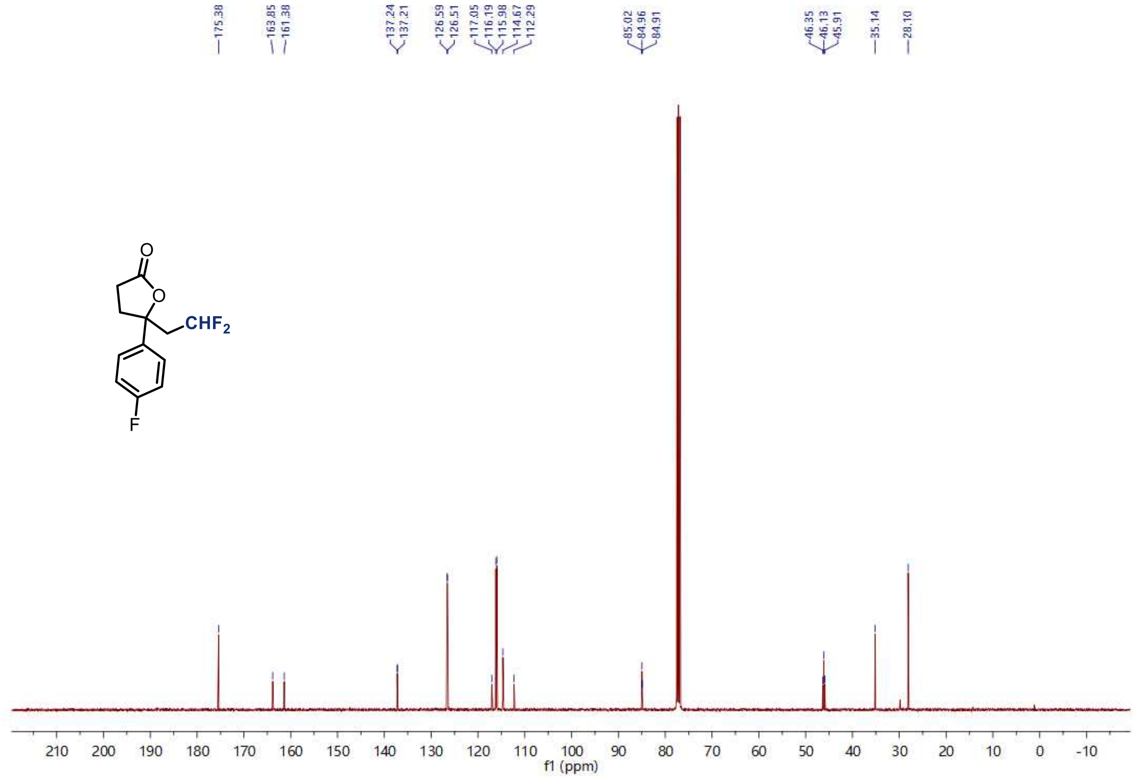

^{19}F-NMR of compound 2c (377 MHz, $CDCl_3$)

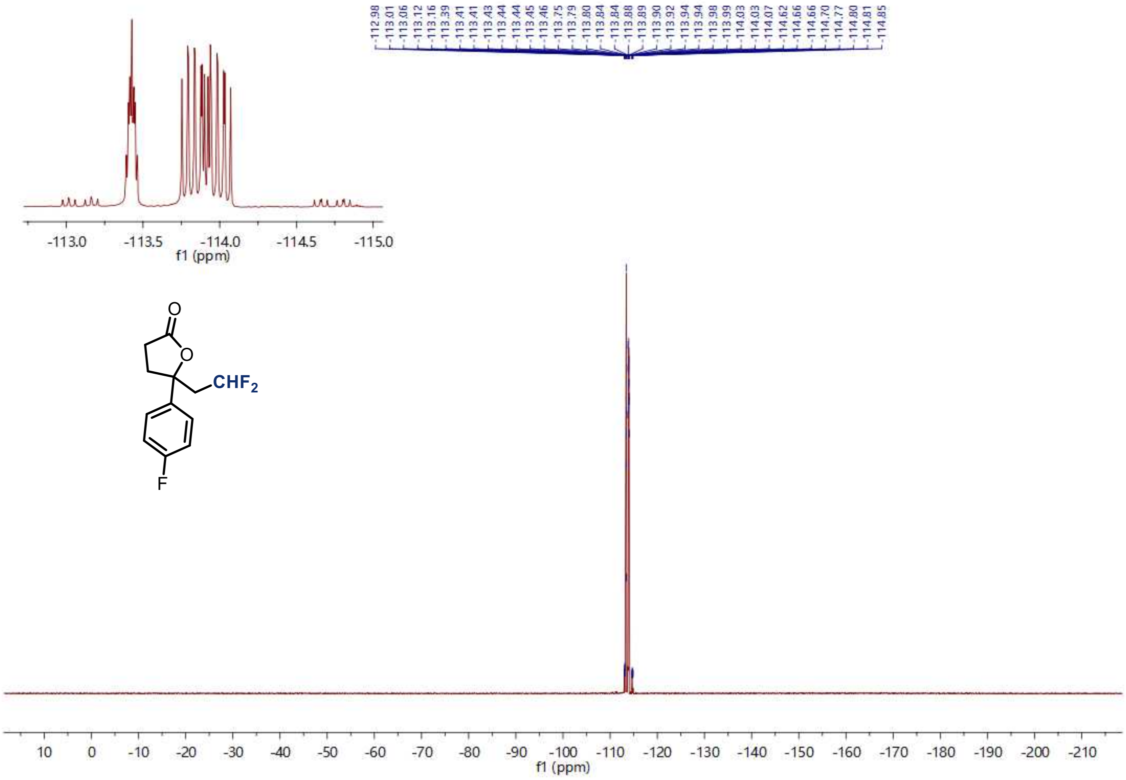

^{1}H-NMR of compound 2d (400 MHz, $CDCl_3$)

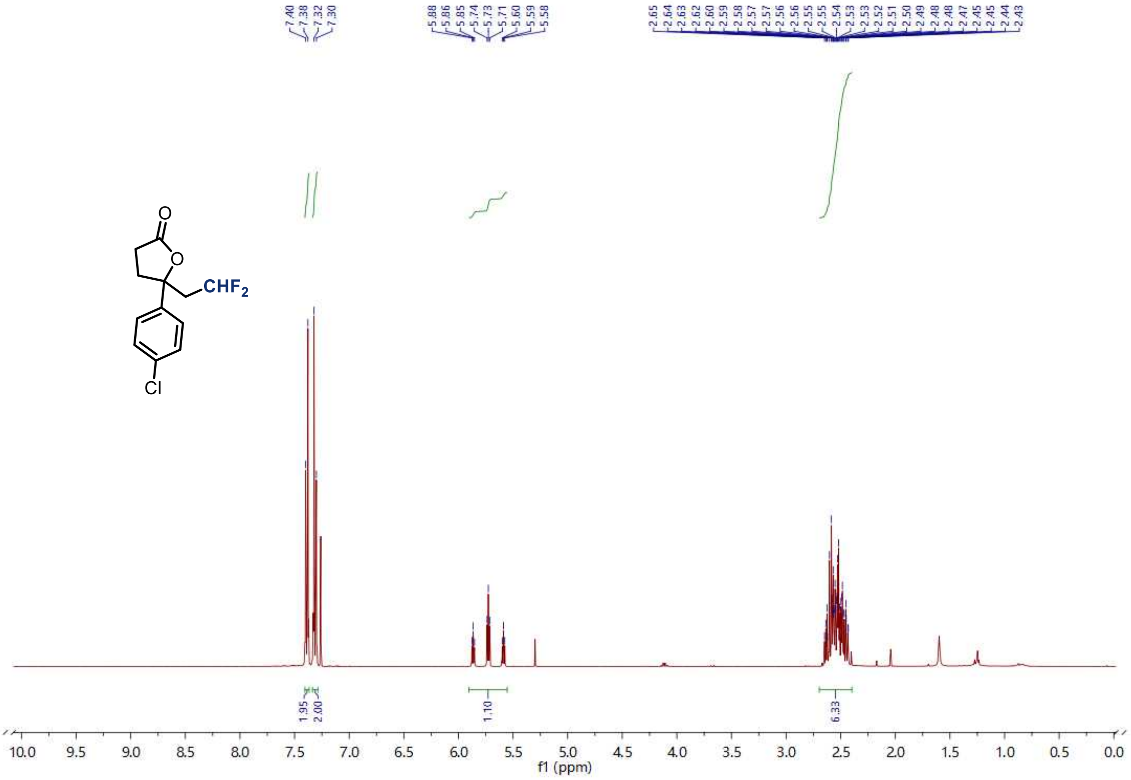

^{13}C-NMR of compound 2d (101 MHz, $CDCl_3$)

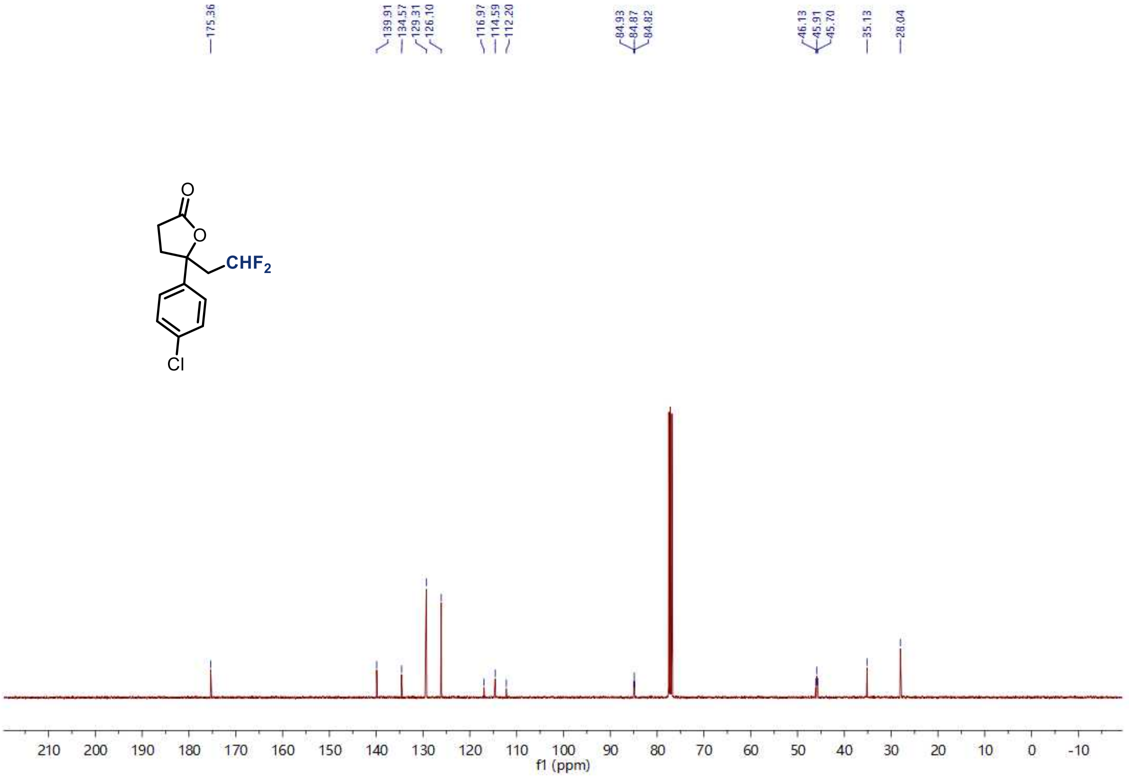

^{19}F-NMR of compound 2d (377 MHz, $CDCl_3$)

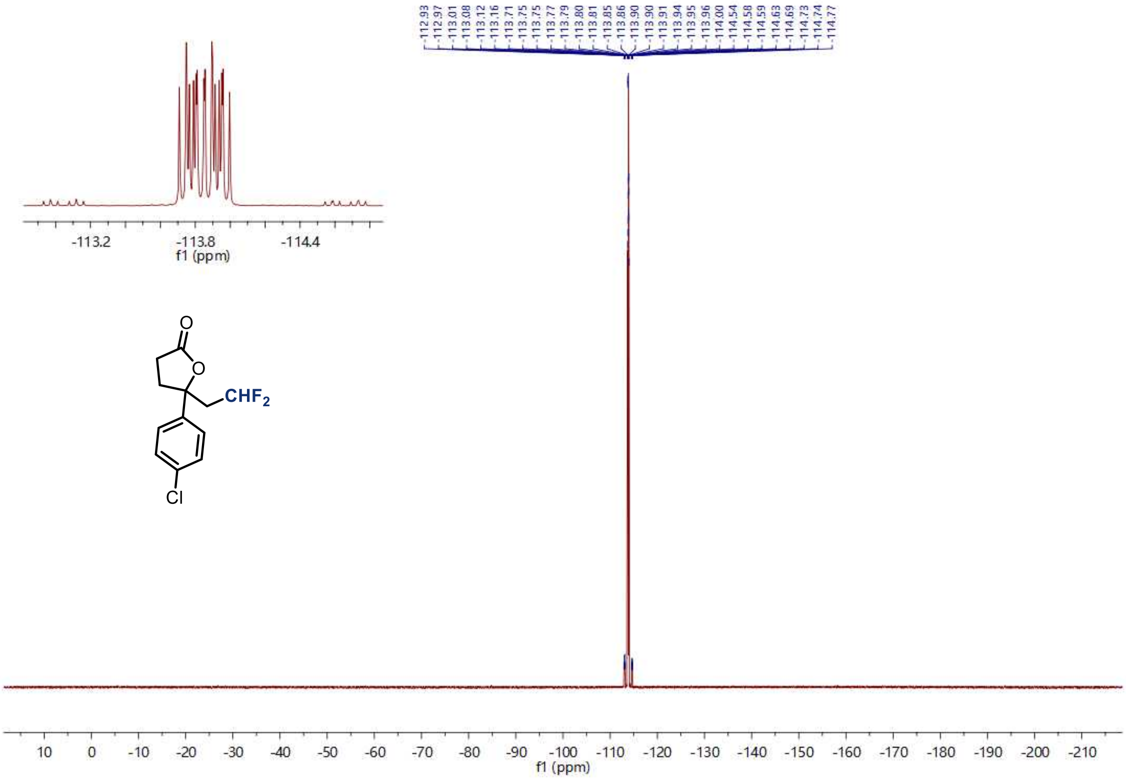

^{1}H-NMR of compound 2e (400 MHz, $CDCl_3$)

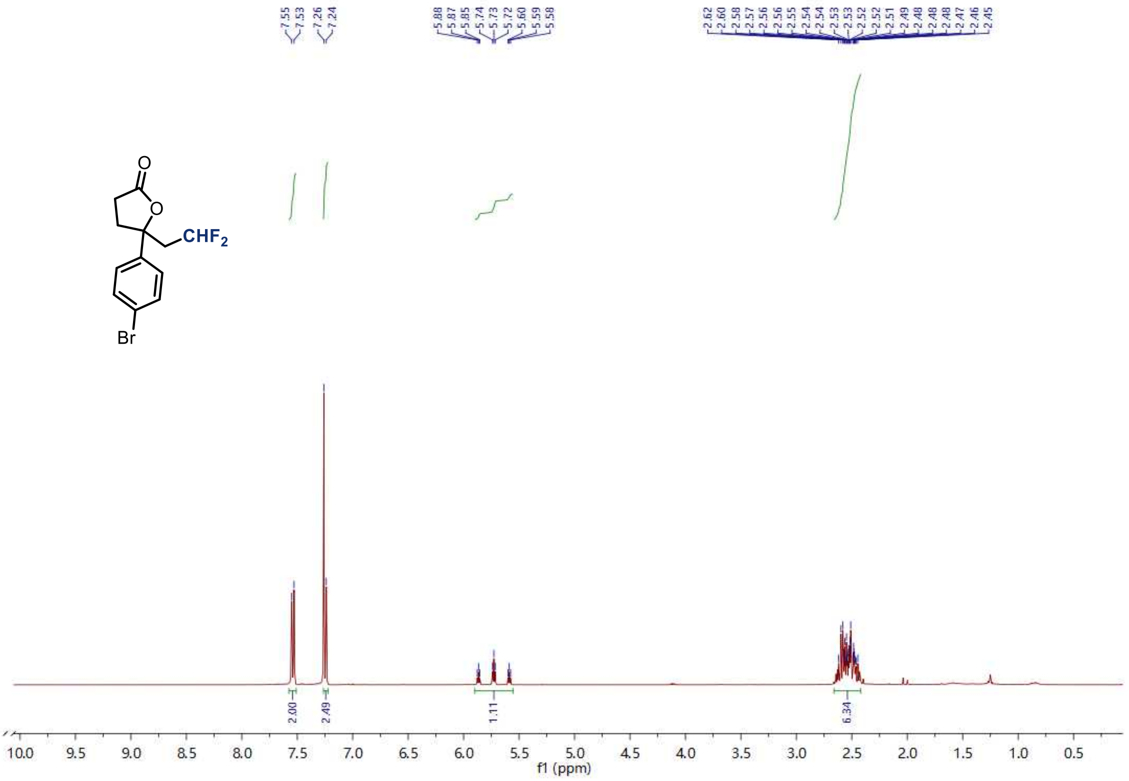

^{13}C-NMR of compound 2e (101 MHz, $CDCl_3$)

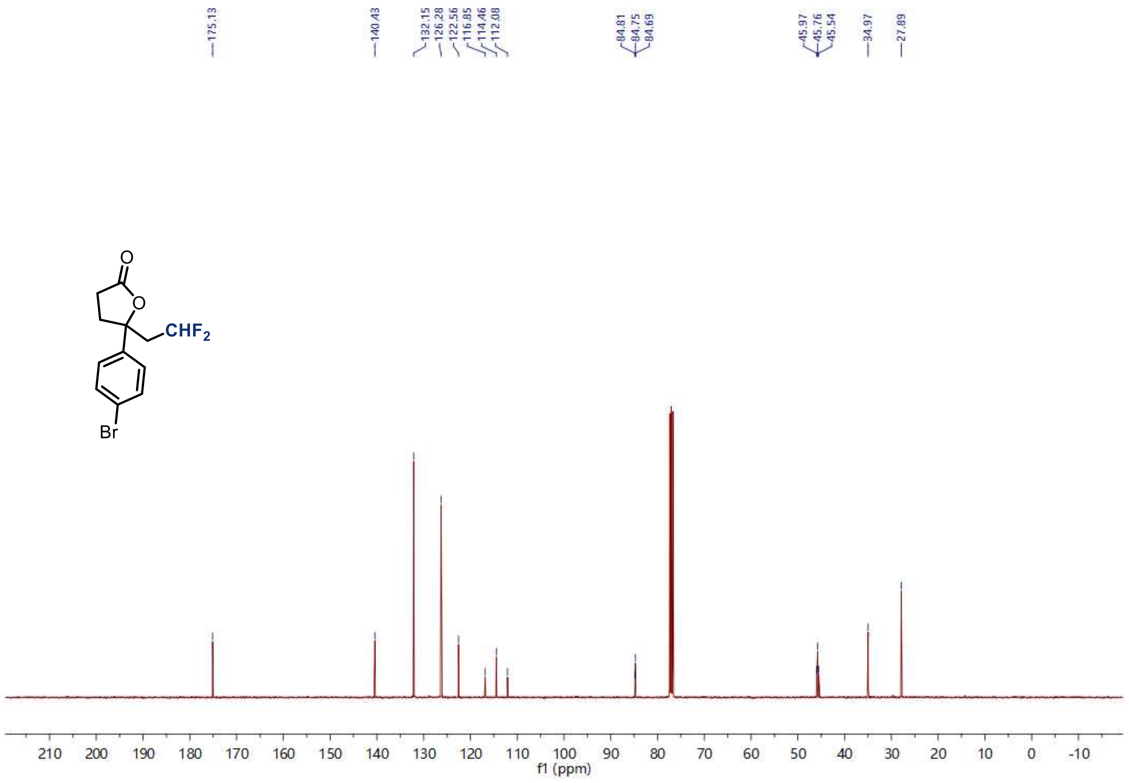

^{19}F-NMR of compound 2e (377 MHz, $CDCl_3$)

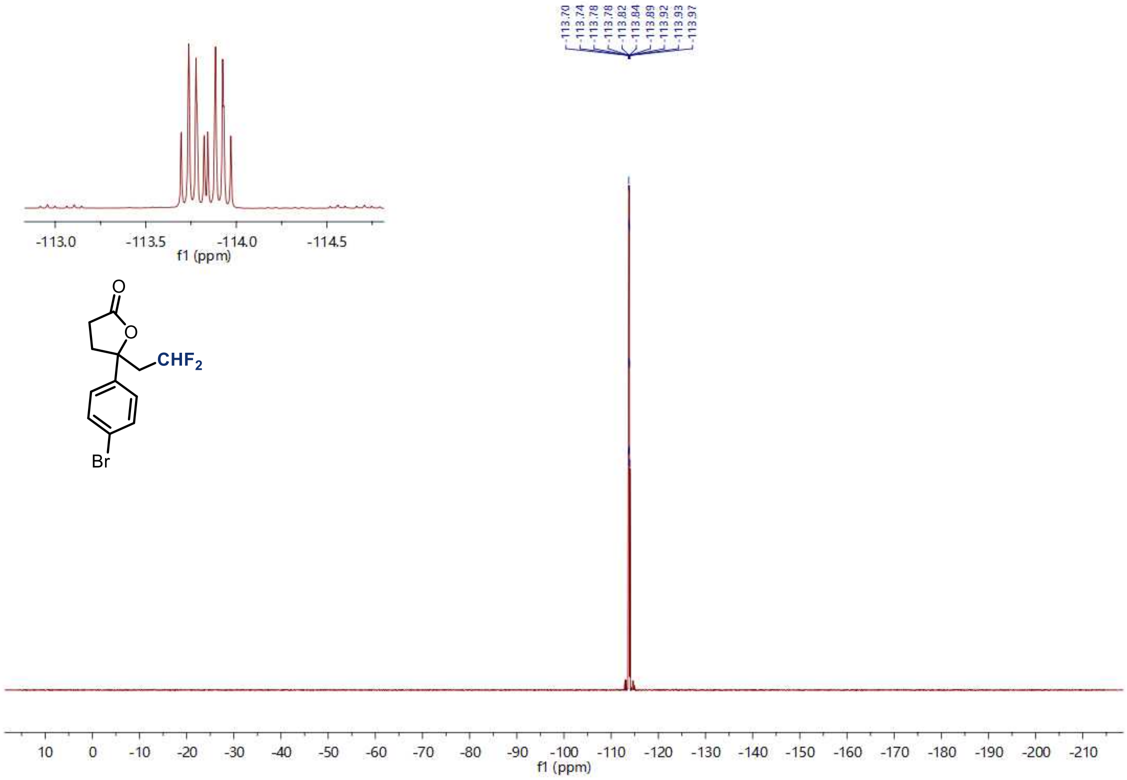

^{1}H-NMR of compound 2f (400 MHz, $CDCl_3$)

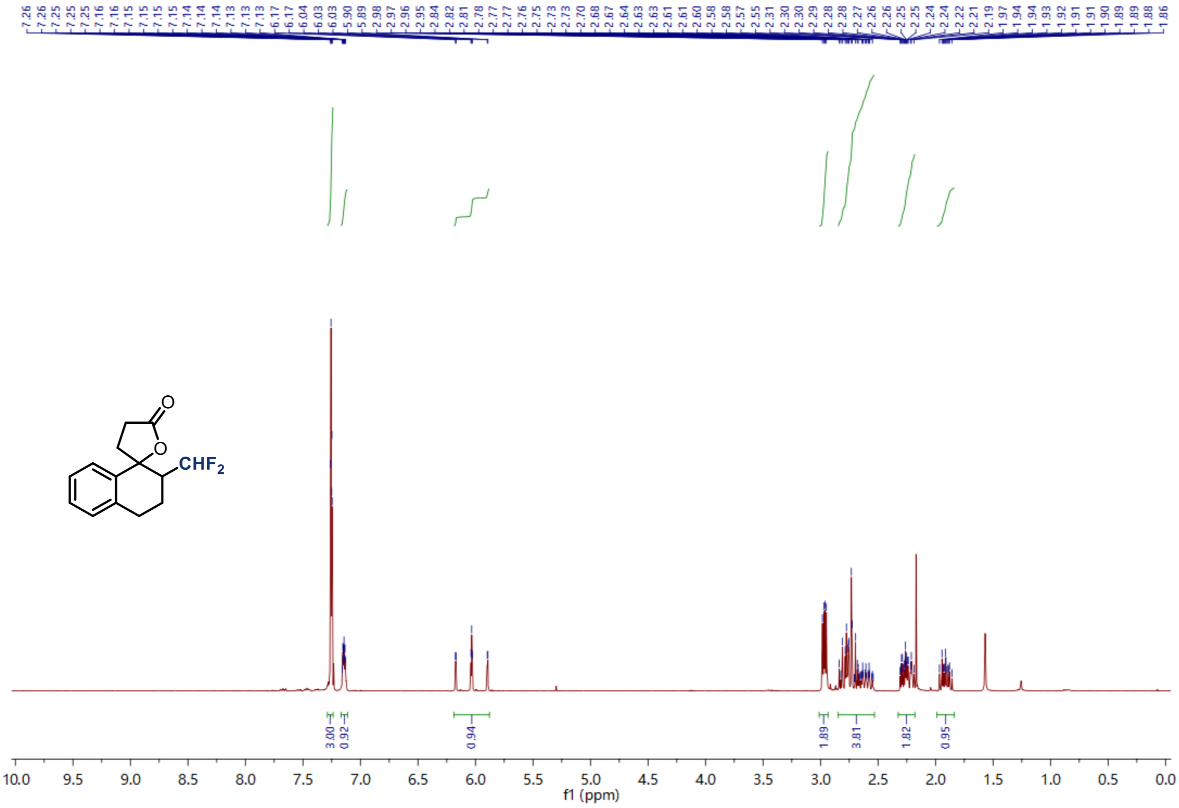

^{13}C-NMR of compound 2f (101 MHz, $CDCl_3$)

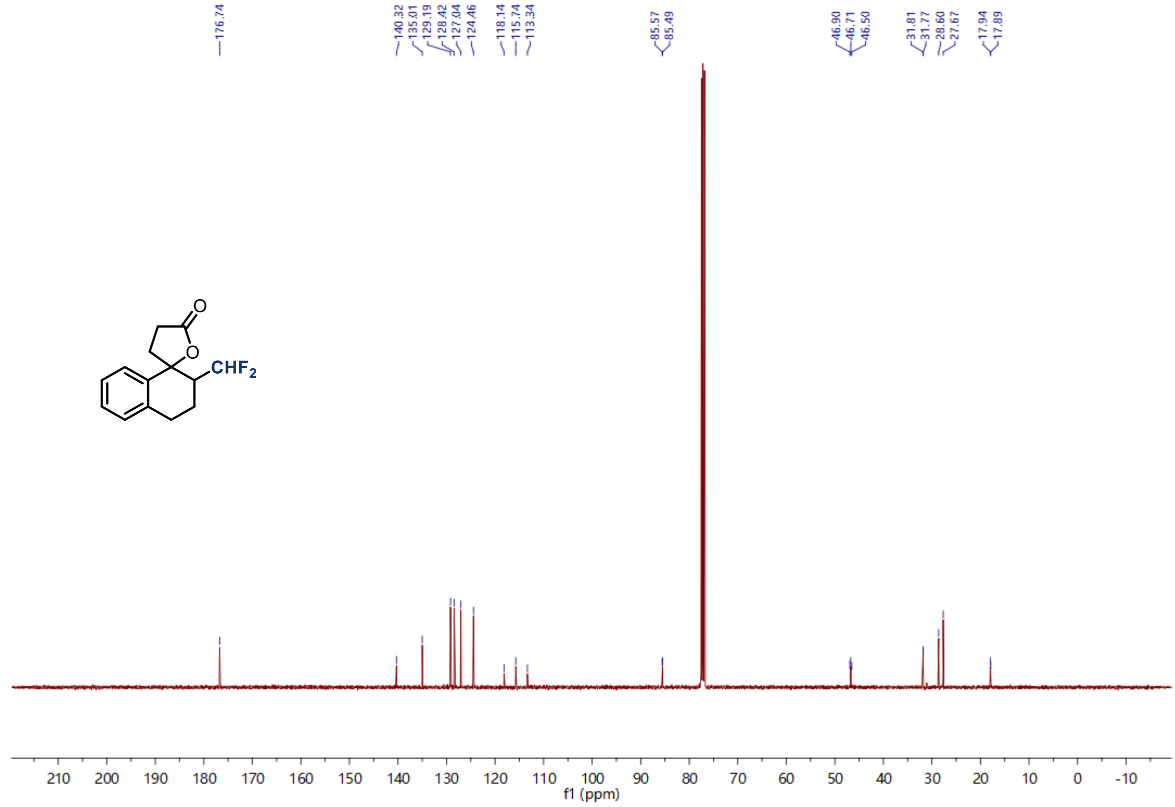

^{19}F-NMR of compound 2f (377 MHz, $CDCl_3$)

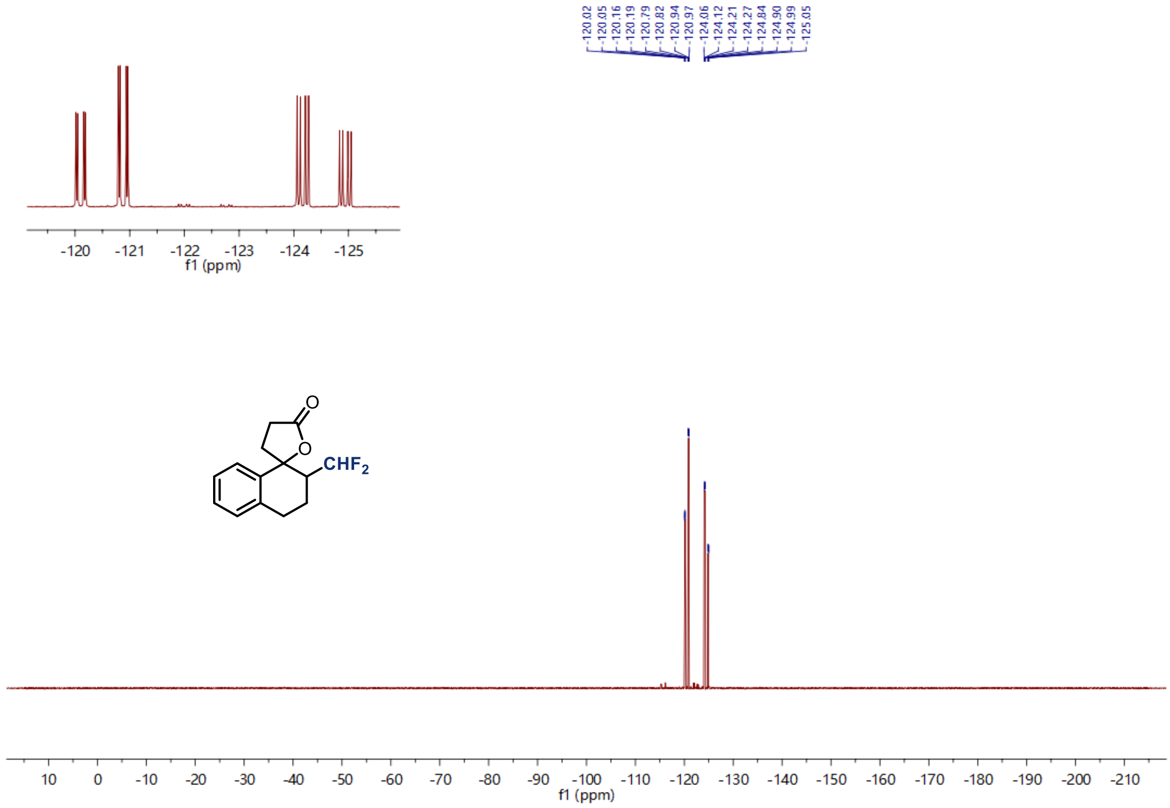

^{1}H-NMR of compound 2g (400 MHz, $CDCl_3$)

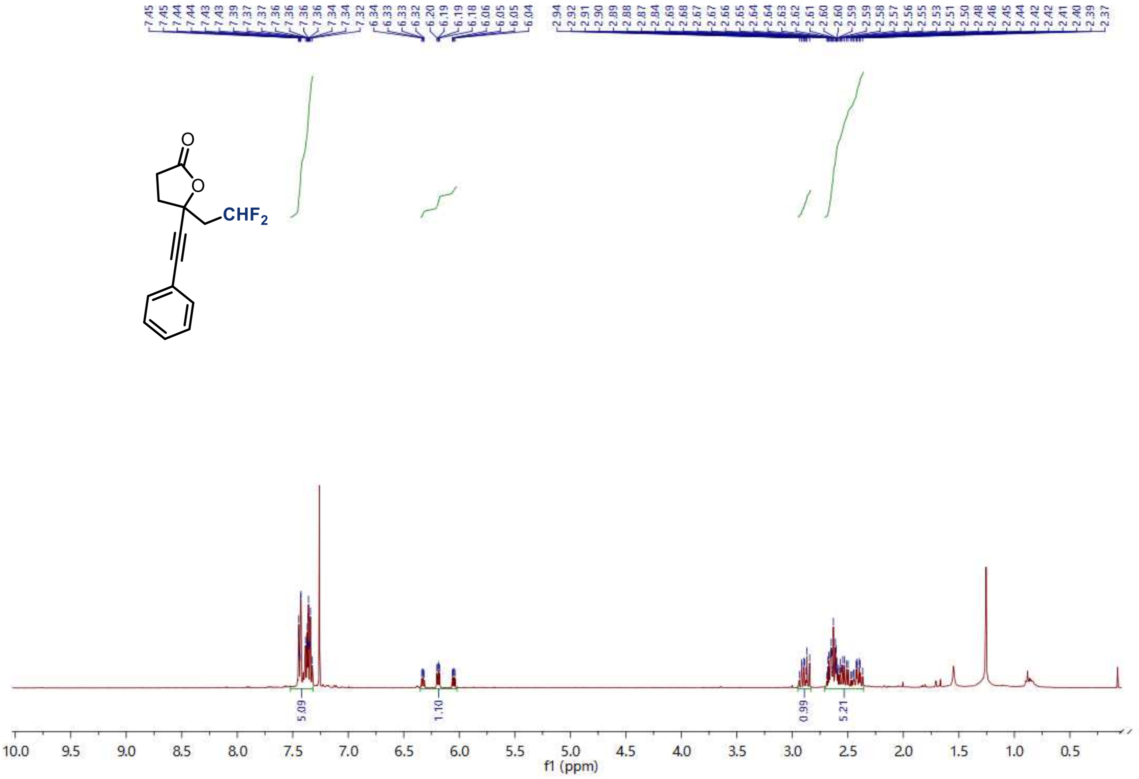

^{13}C-NMR of compound 2g (101 MHz, $CDCl_3$)

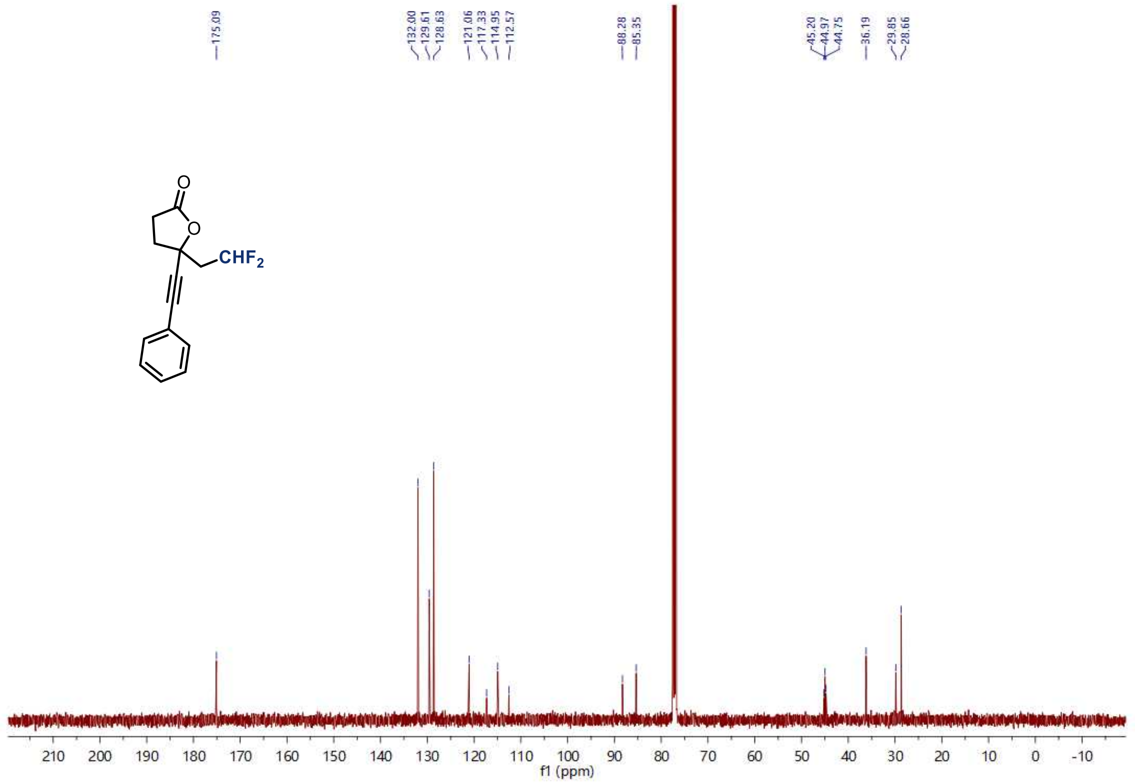

^{19}F-NMR of compound 2g (377 MHz, $CDCl_3$)

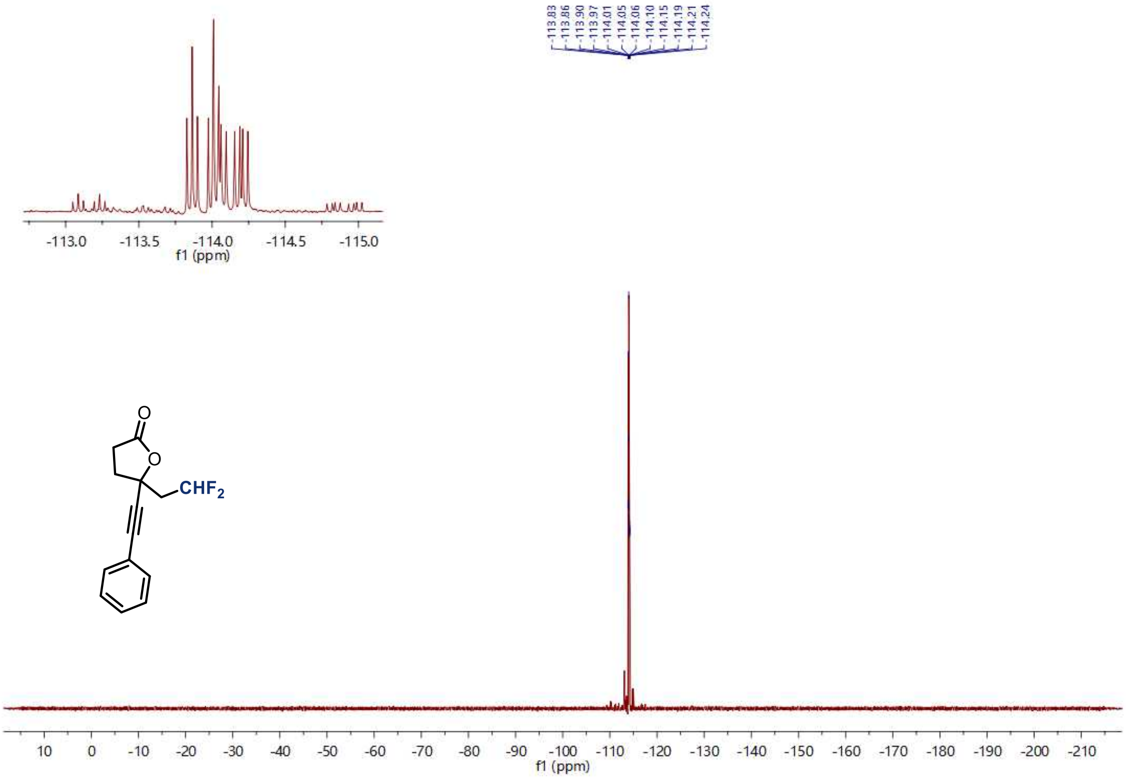

^{1}H-NMR of compound 2h (400 MHz, $CDCl_3$)

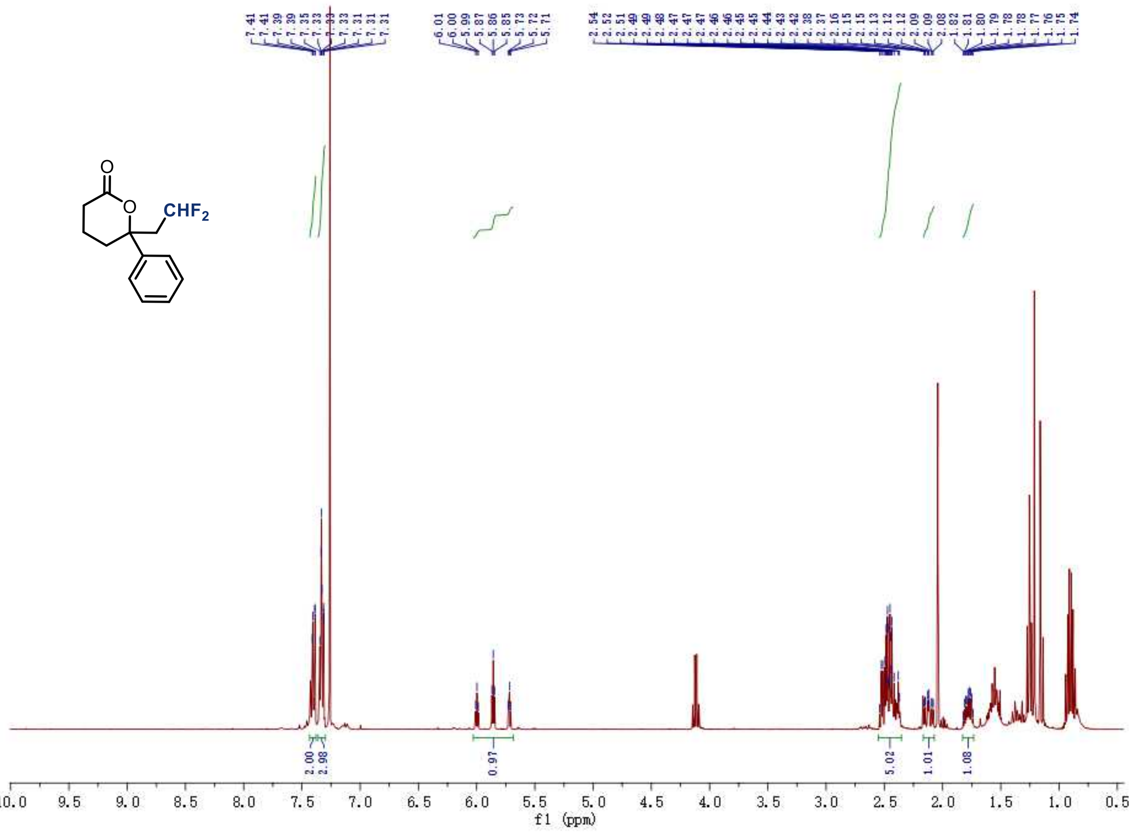

^{13}C-NMR of compound 2h (101 MHz, $CDCl_3$)

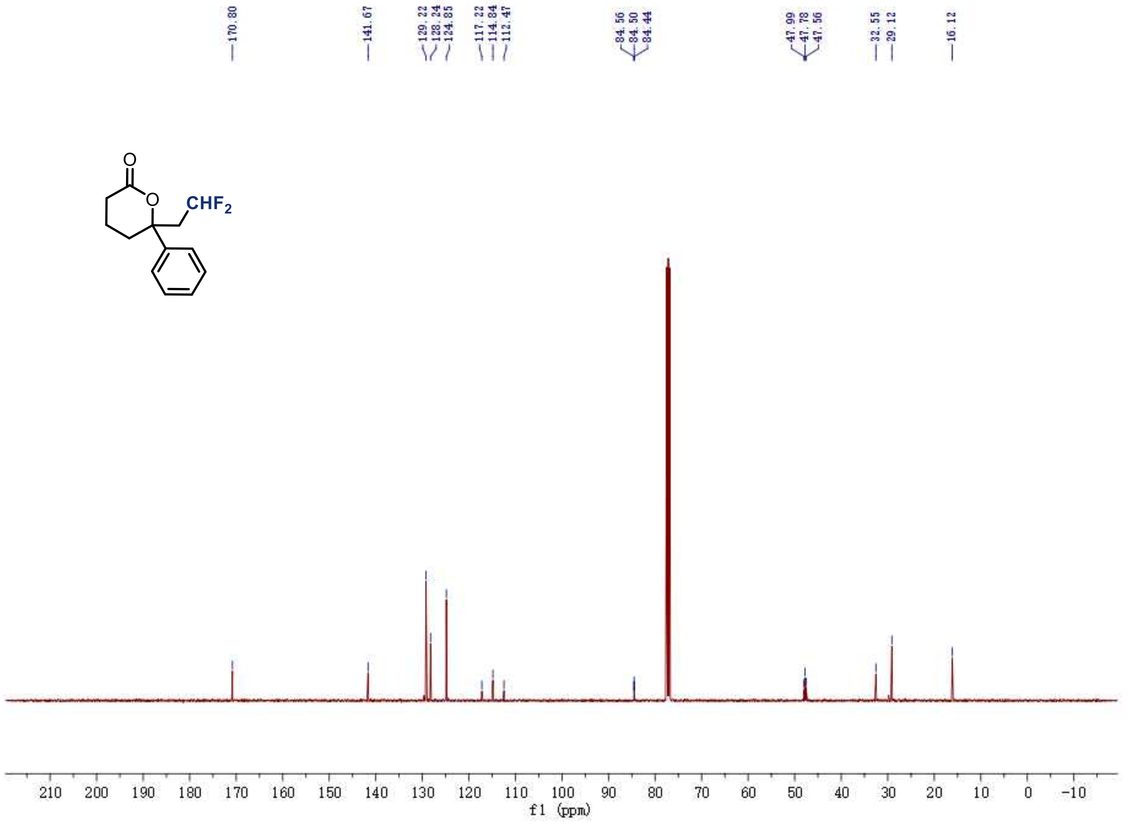

^{19}F-NMR of compound 2h (377 MHz, $CDCl_3$)

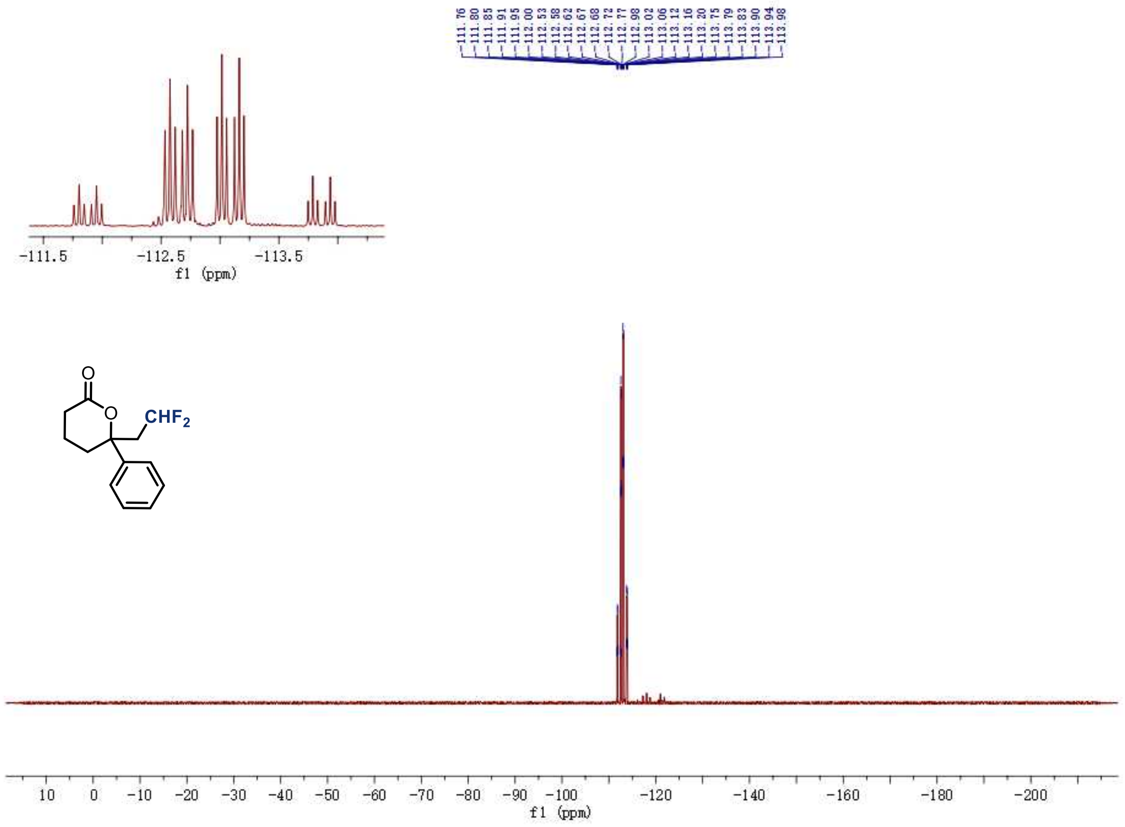

^{1}H-NMR of compound 2i (400 MHz, $CDCl_3$)

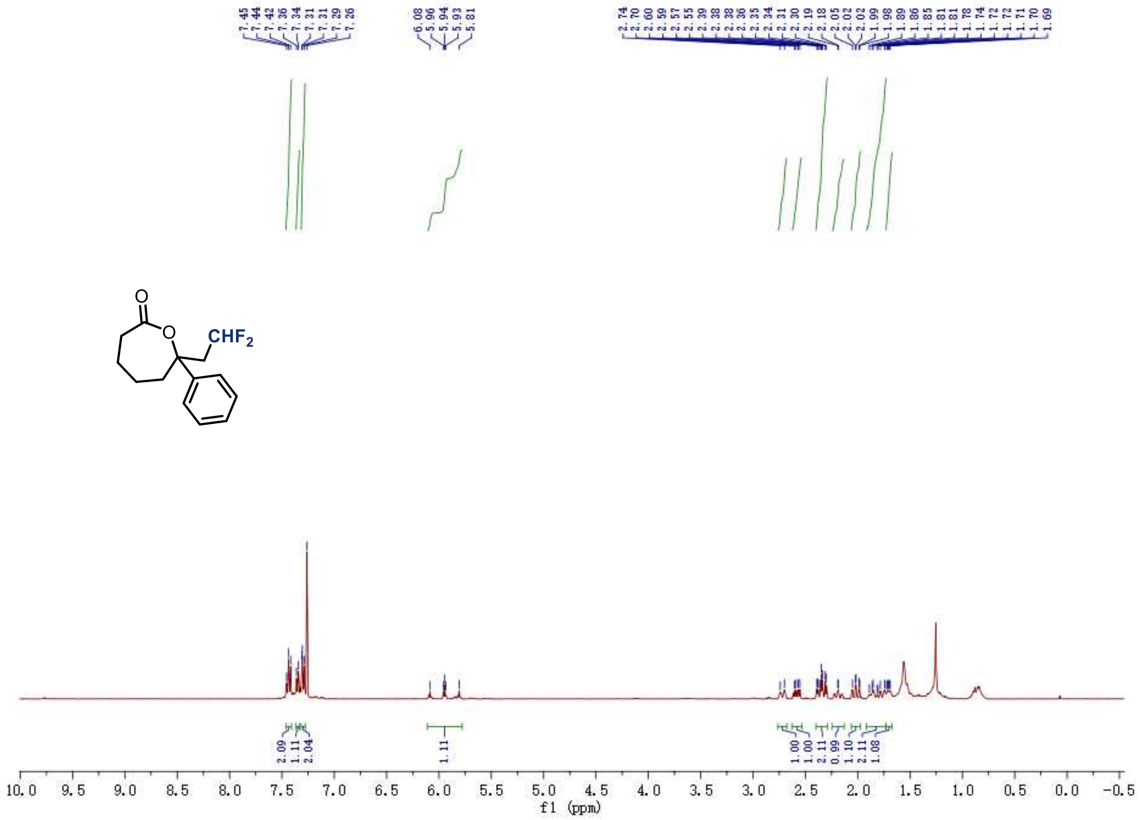

^{13}C-NMR of compound 2i (101 MHz, $CDCl_3$)

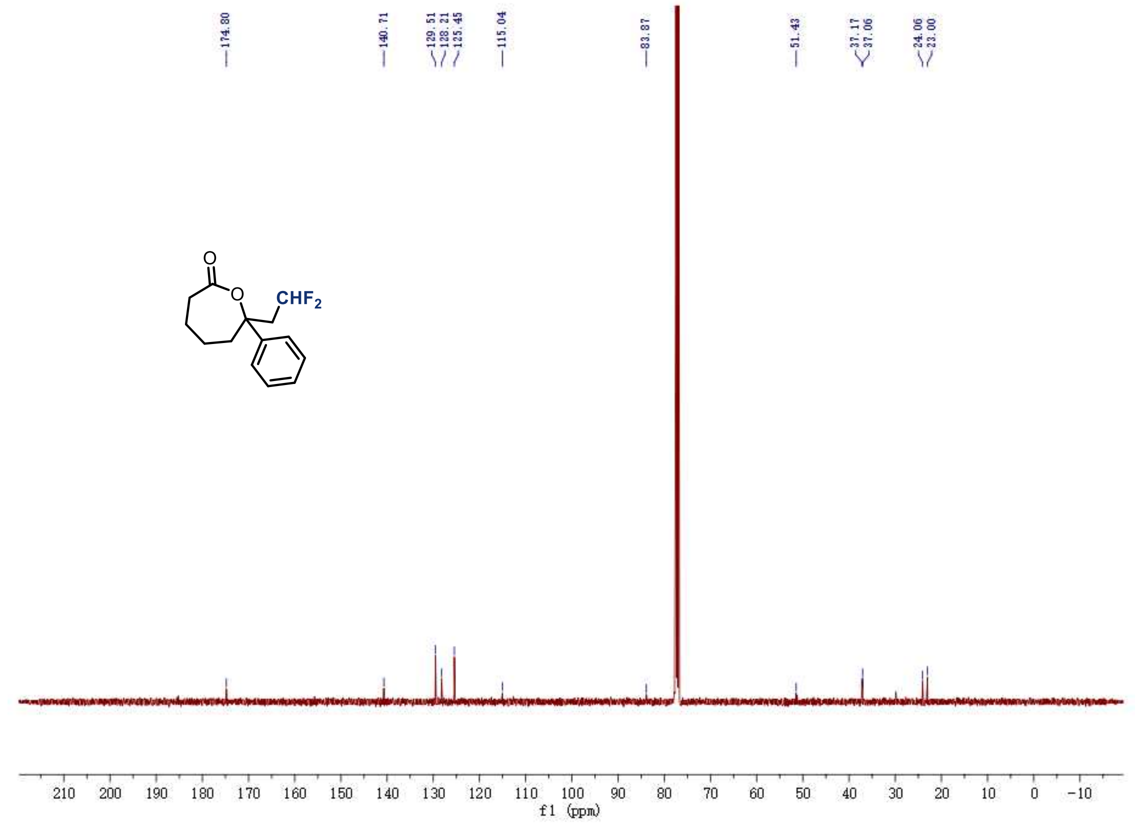

^{19}F-NMR of compound 2i (377 MHz, $CDCl_3$)

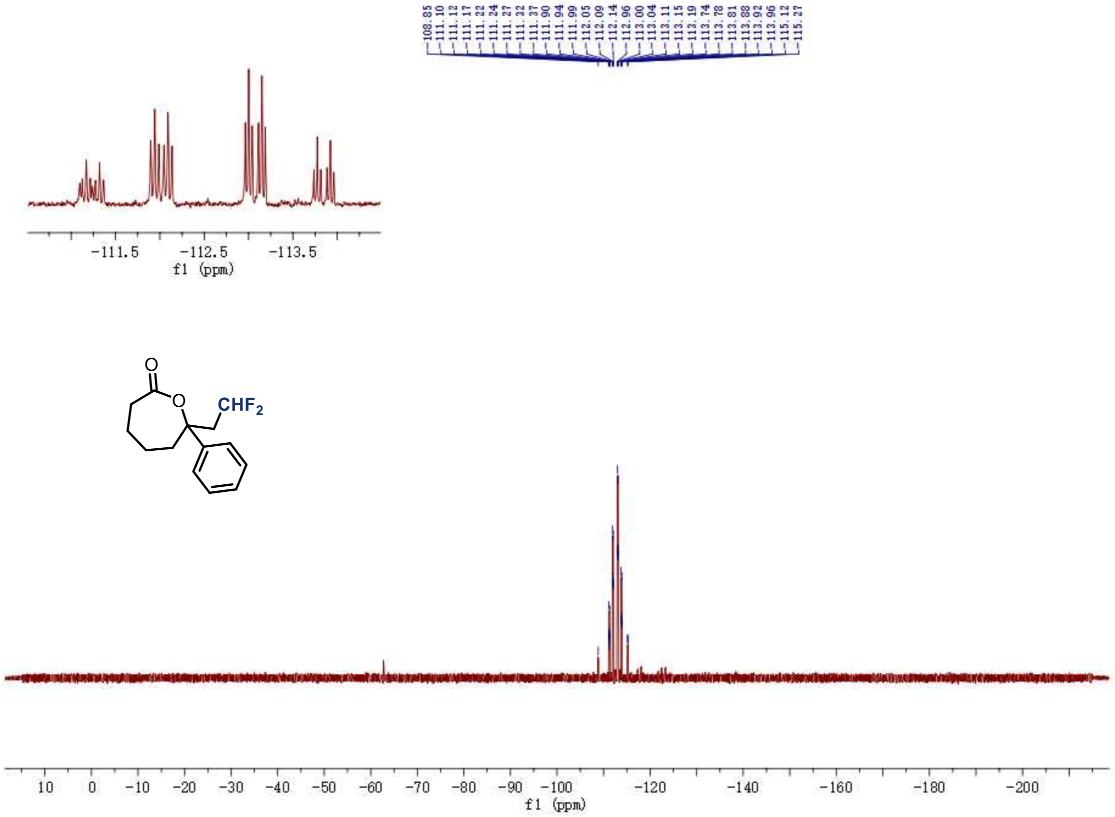

^{1}H-NMR of compound 4a (400 MHz, $CDCl_3$)

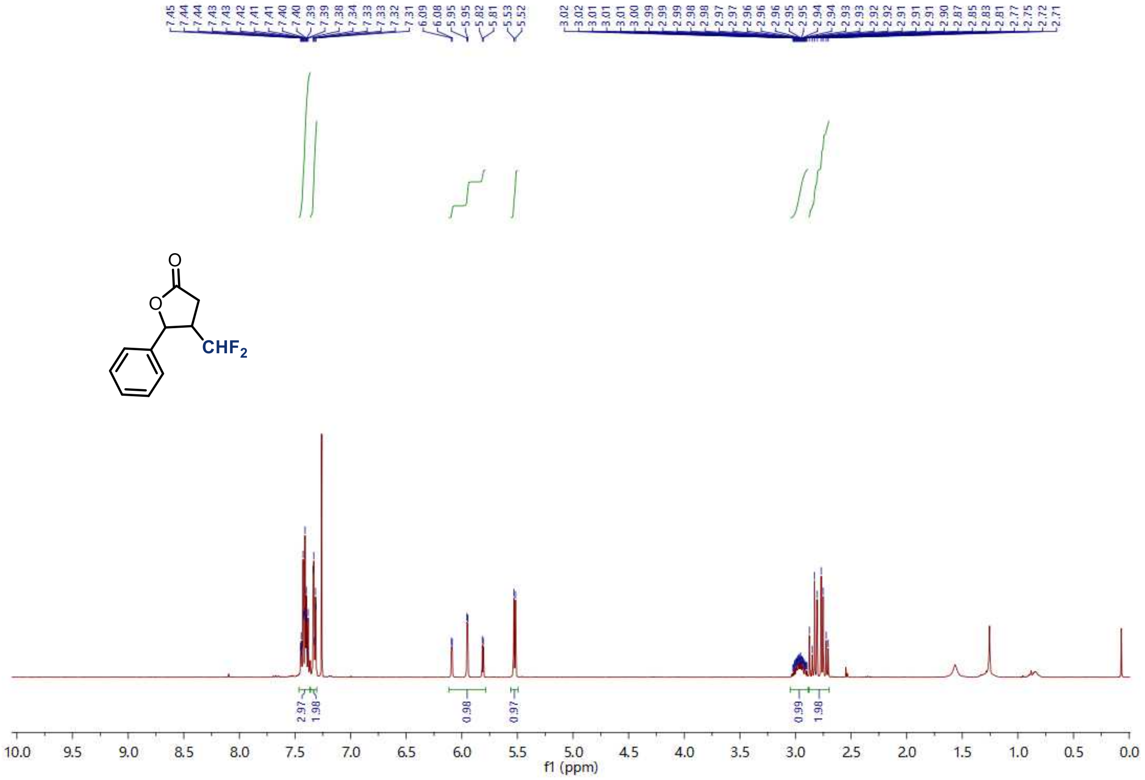

^{13}C-NMR of compound 4a (101 MHz, $CDCl_3$)

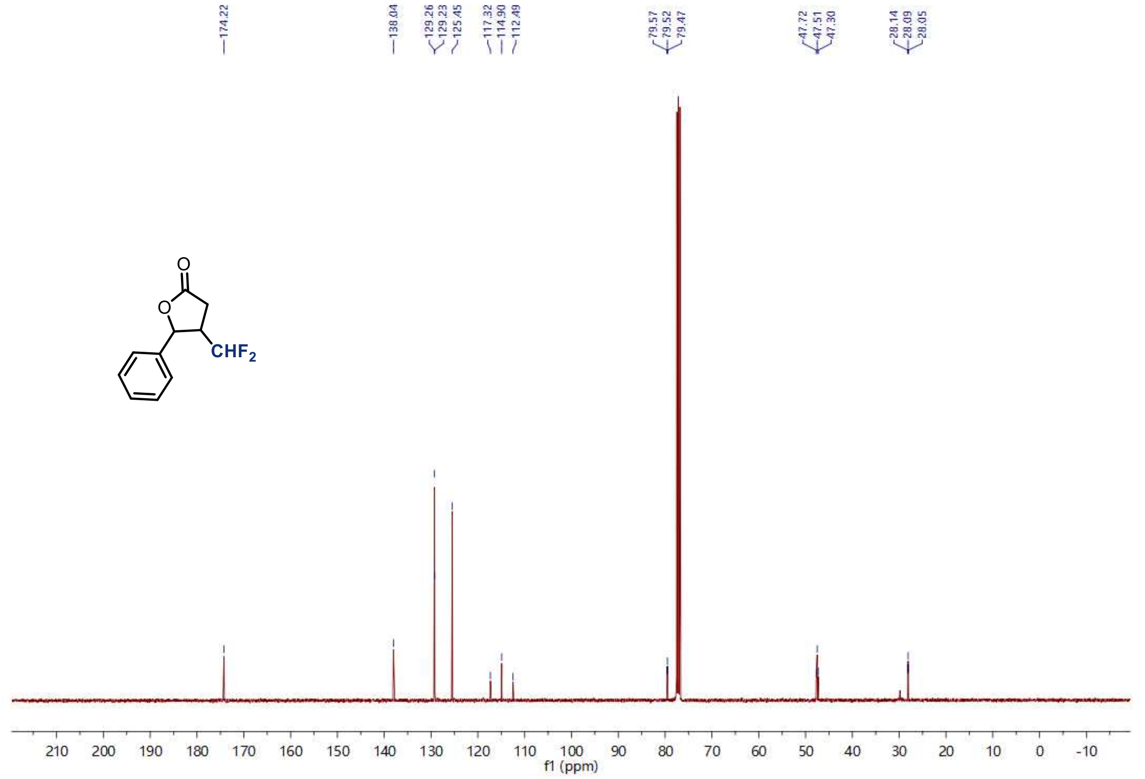

^{19}F-NMR of compound 4a (377 MHz, $CDCl_3$)

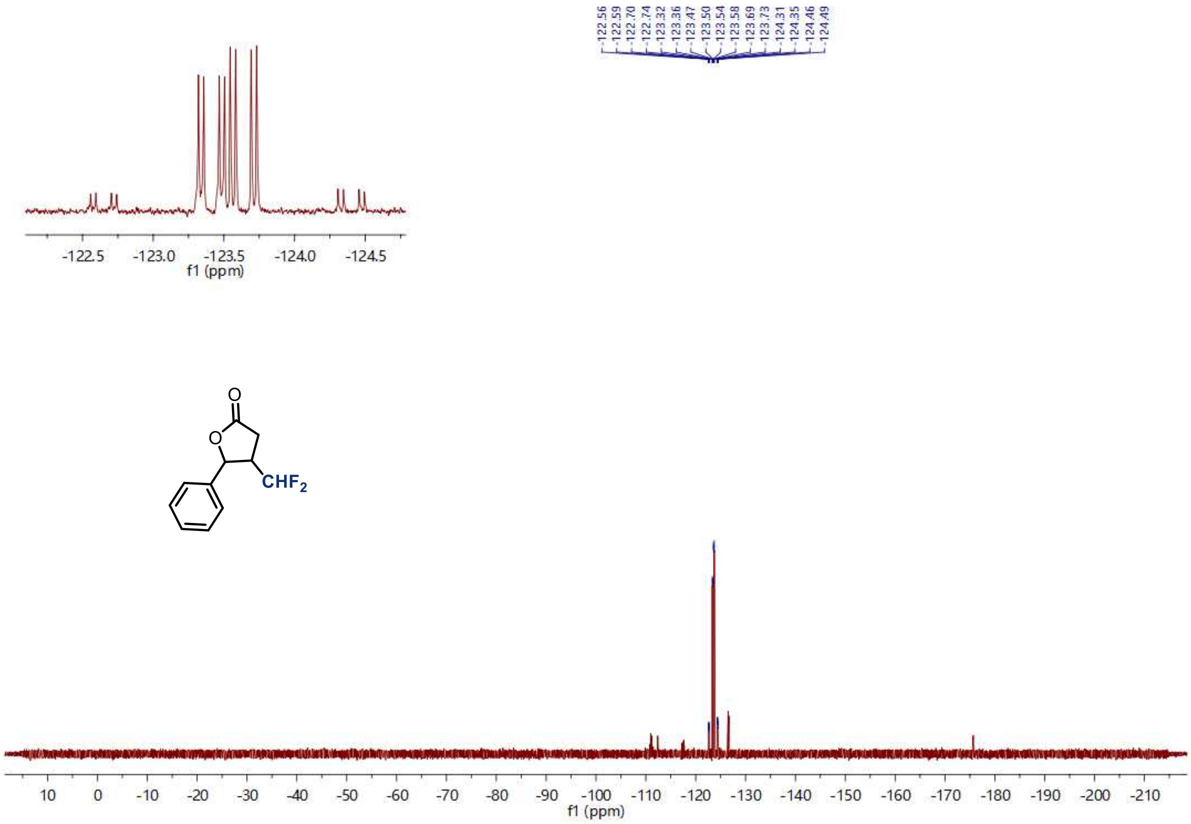

^{1}H-NMR of compound 4b (400 MHz, $CDCl_3$)

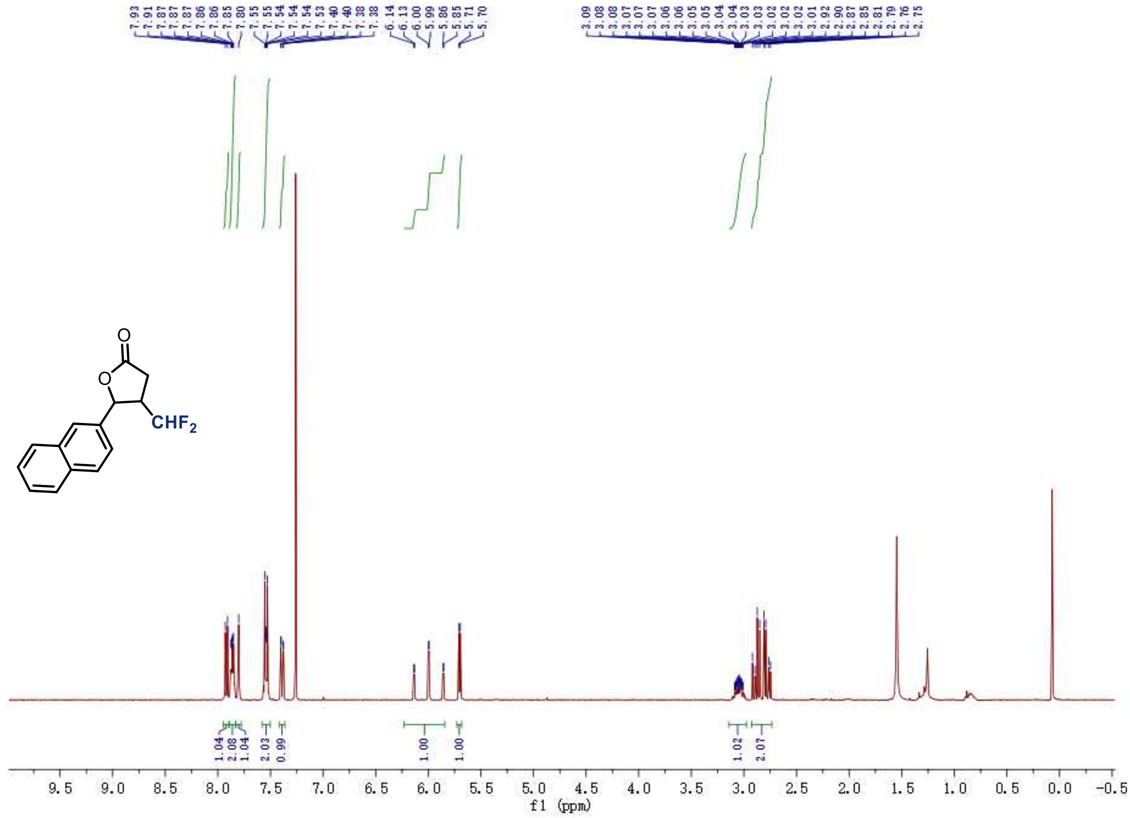

^{13}C-NMR of compound 4b (101 MHz, $CDCl_3$)

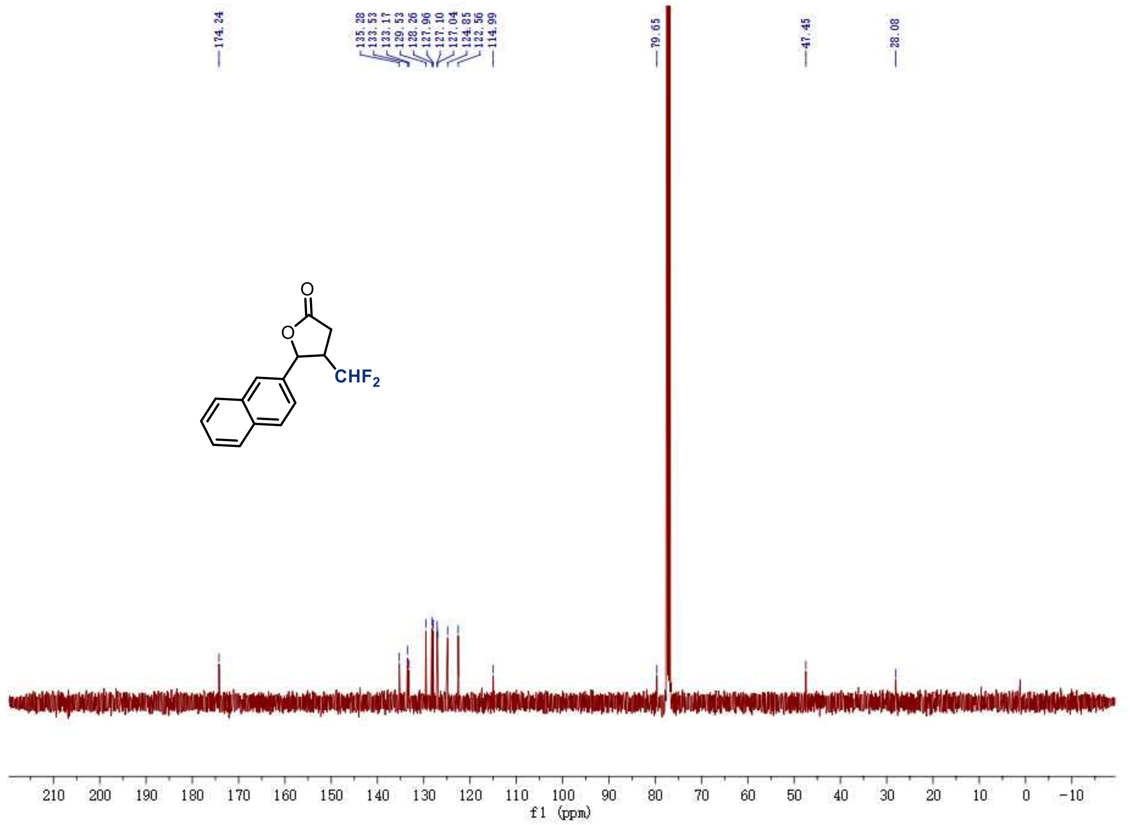

^{19}F-NMR of compound 4b (377 MHz, $CDCl_3$)

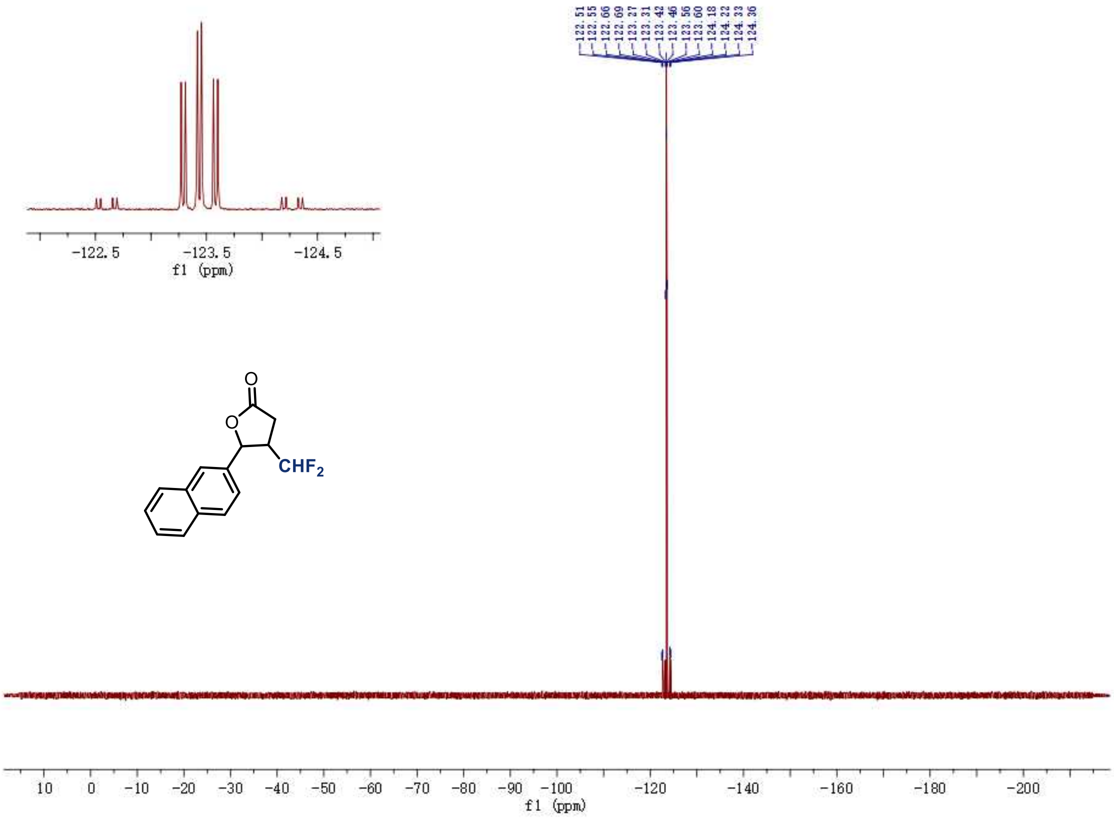

^{1}H-NMR of compound 4c (400 MHz, $CDCl_3$)

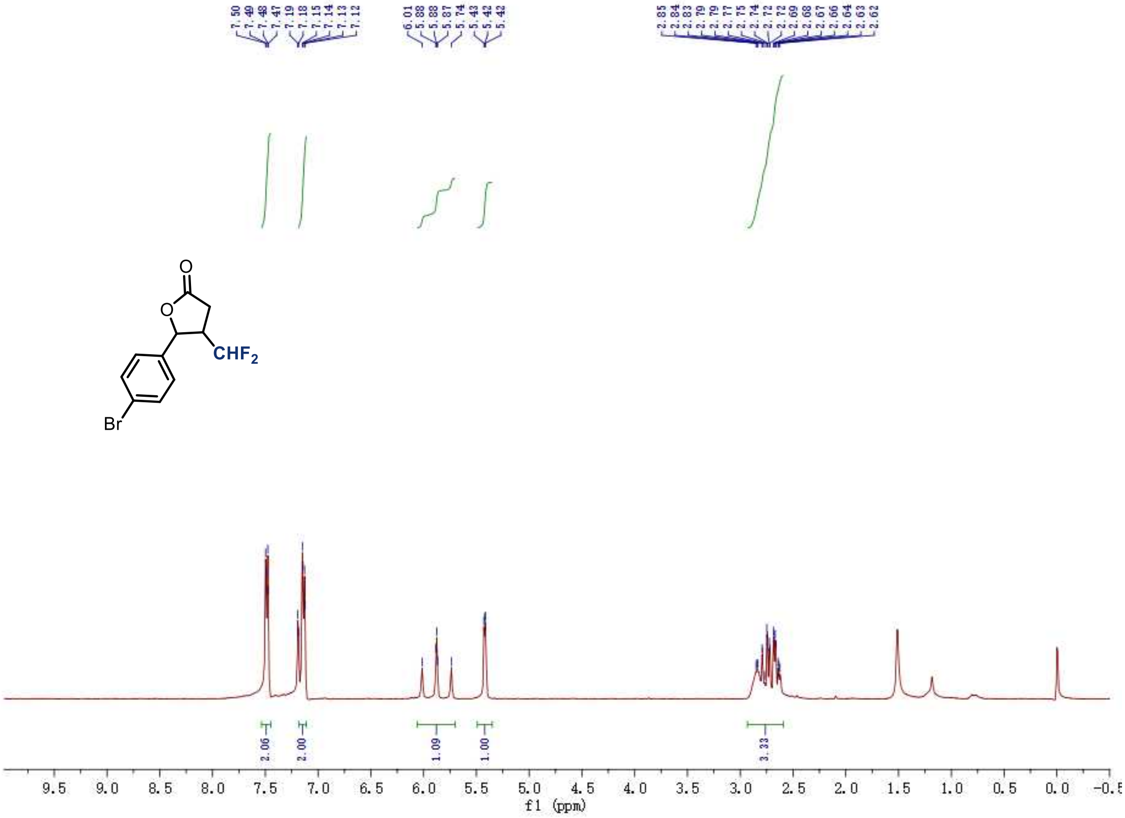

^{13}C-NMR of compound 4c (101 MHz, $CDCl_3$)

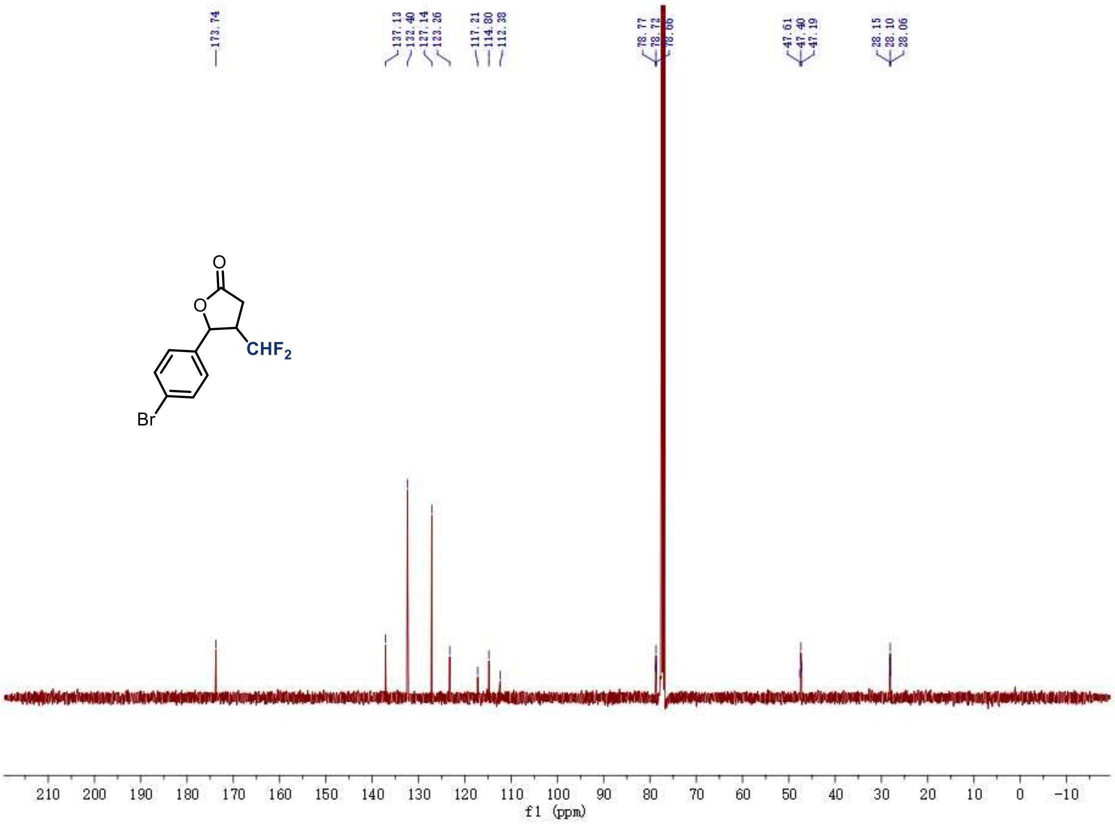

^{19}F-NMR of compound 4c (377 MHz, $CDCl_3$)

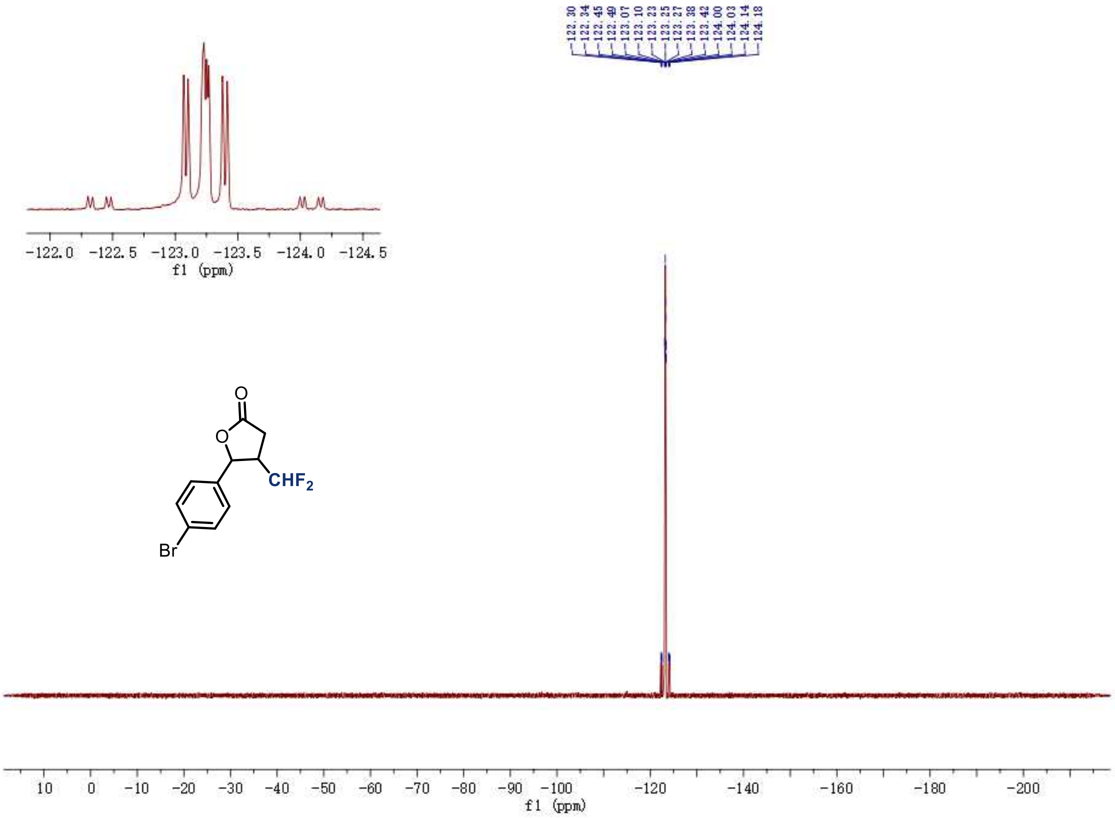

^{1}H-NMR of compound 4d (400 MHz, $CDCl_3$)

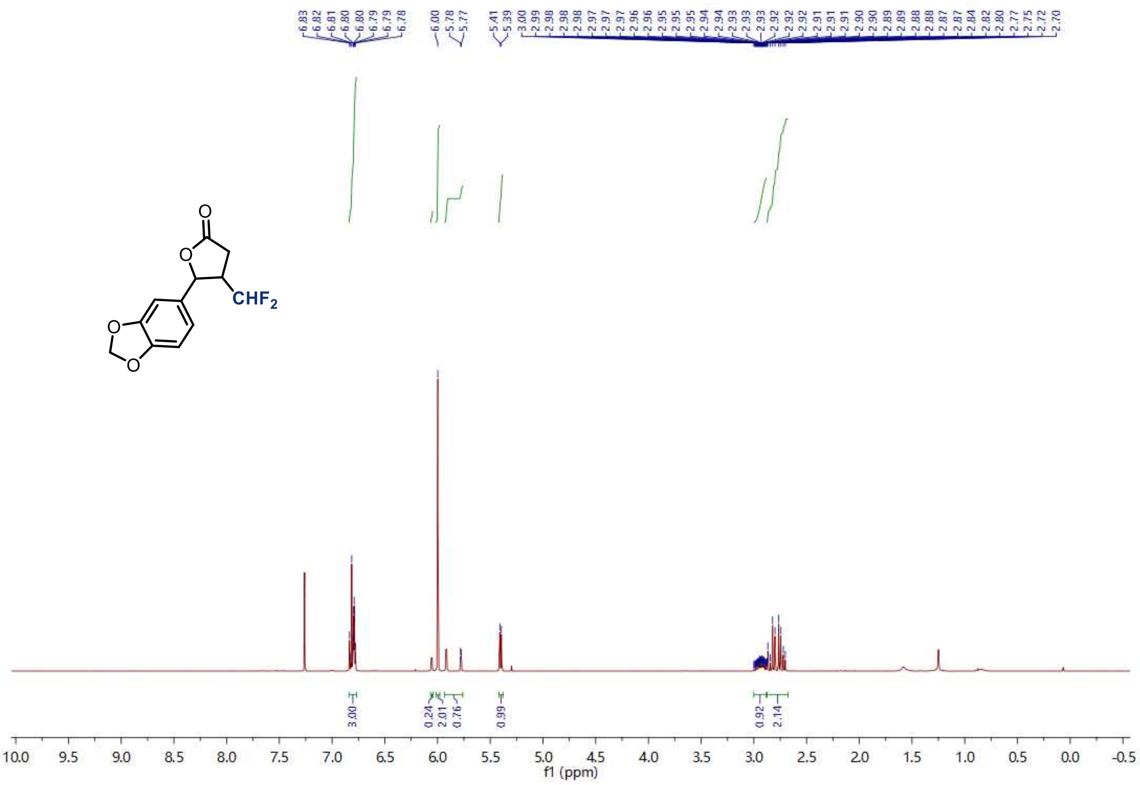

^{13}C-NMR of compound 4d (101 MHz, $CDCl_3$)

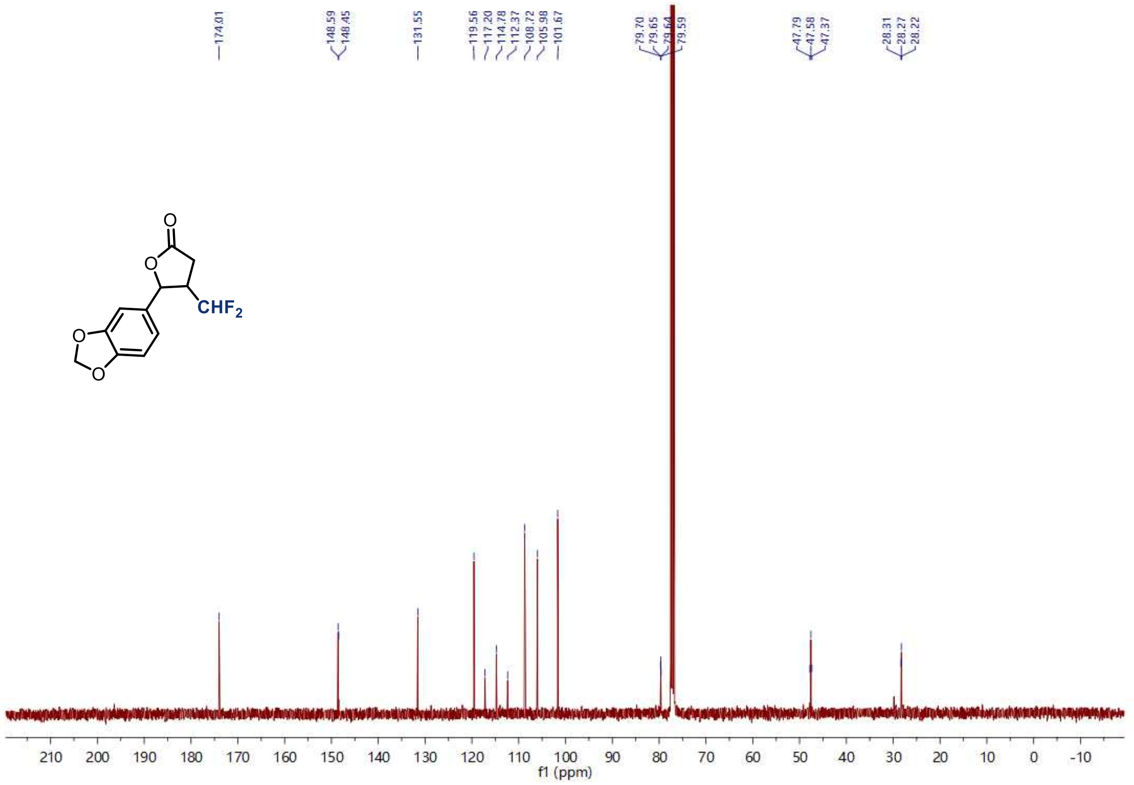

^{19}F-NMR of compound 4d (377 MHz, $CDCl_3$)

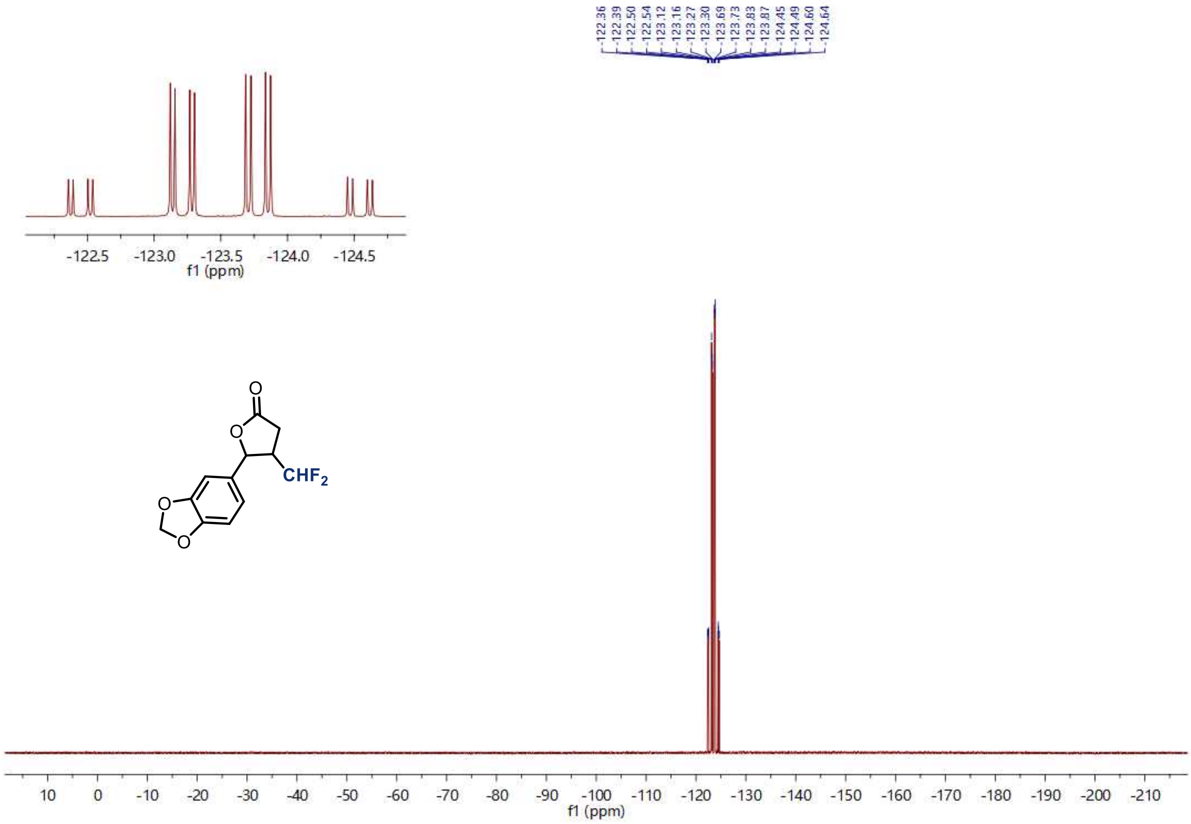

^{1}H-NMR of compound 4e (400 MHz, CDCl$_3$)

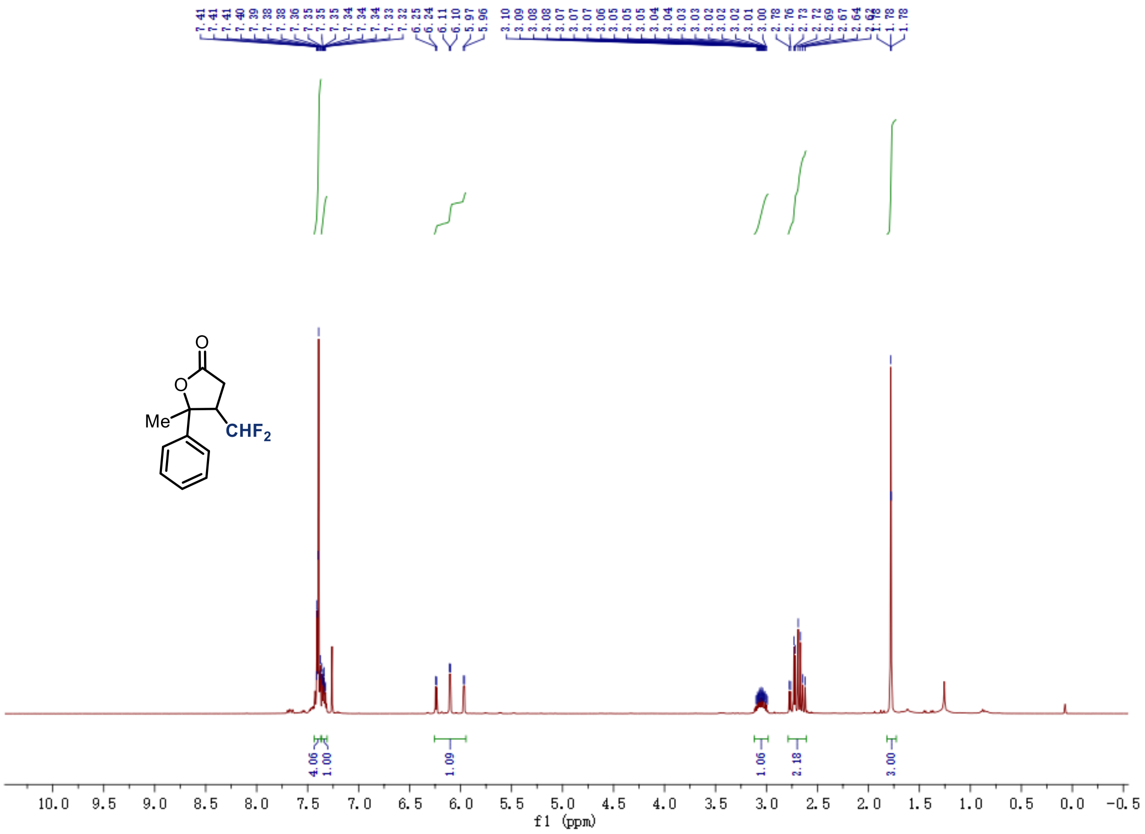

^{13}C-NMR of compound 4e (101 MHz, CDCl$_3$)

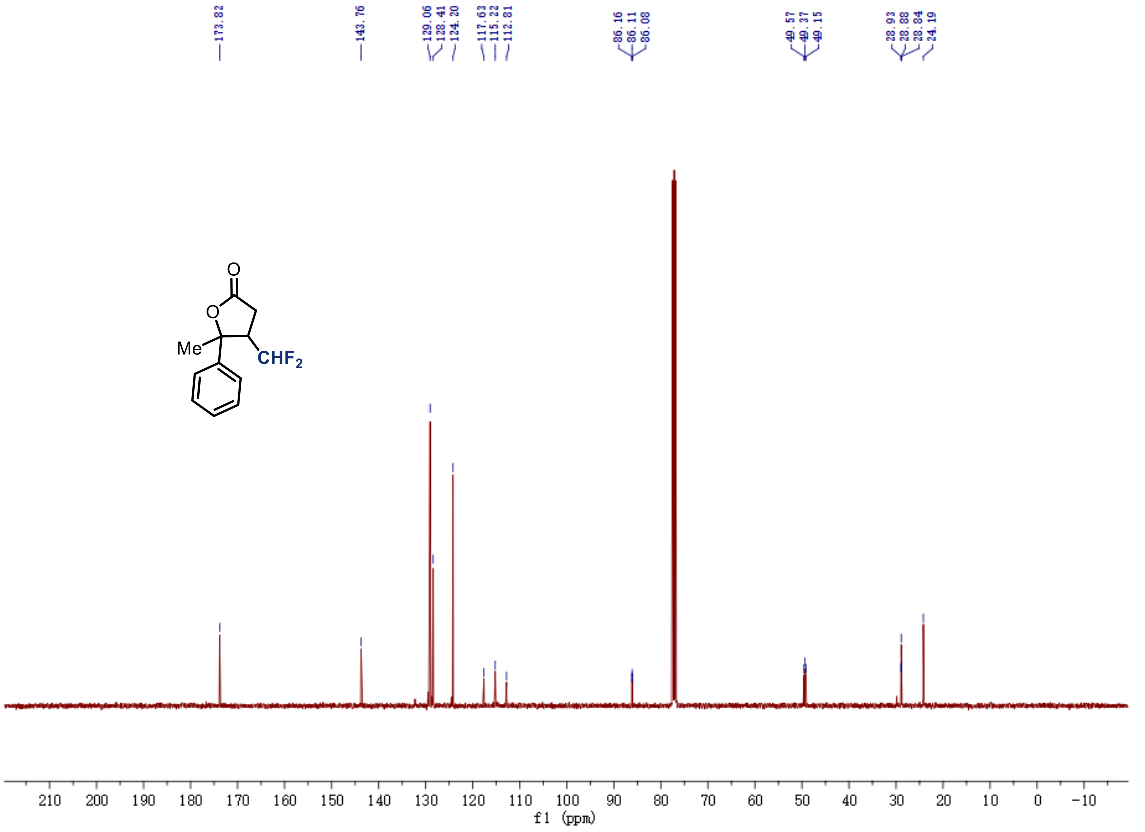

^{19}F-NMR of compound 4e (377 MHz, $CDCl_3$)

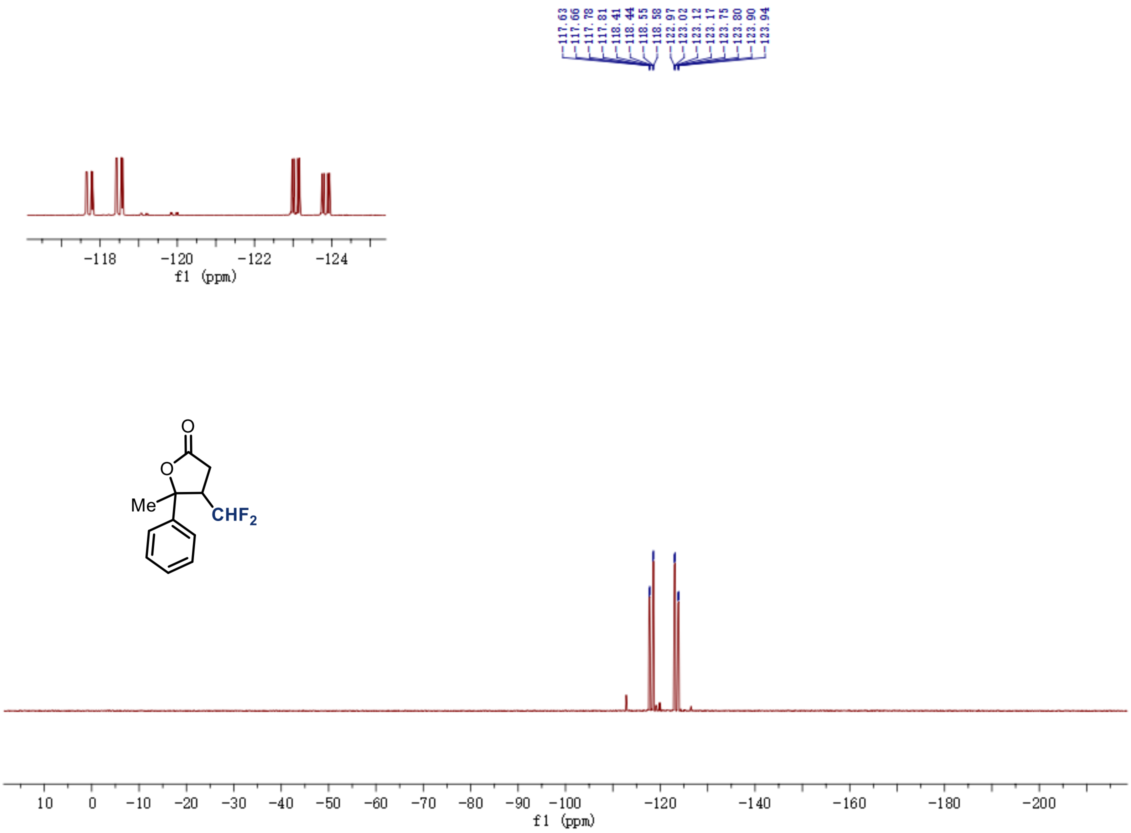

^{1}H-NMR of compound 5b (400 MHz, $CDCl_3$)

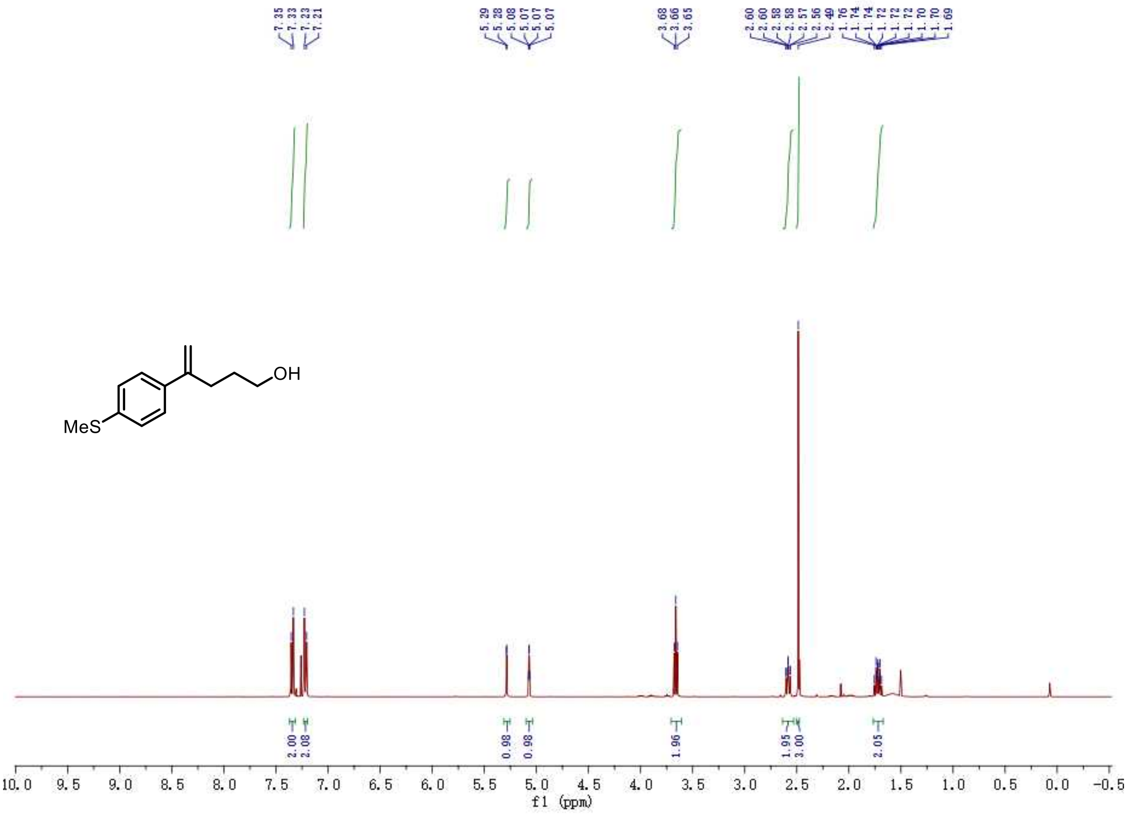

^{13}C-NMR of compound 5b (101 MHz, $CDCl_3$)

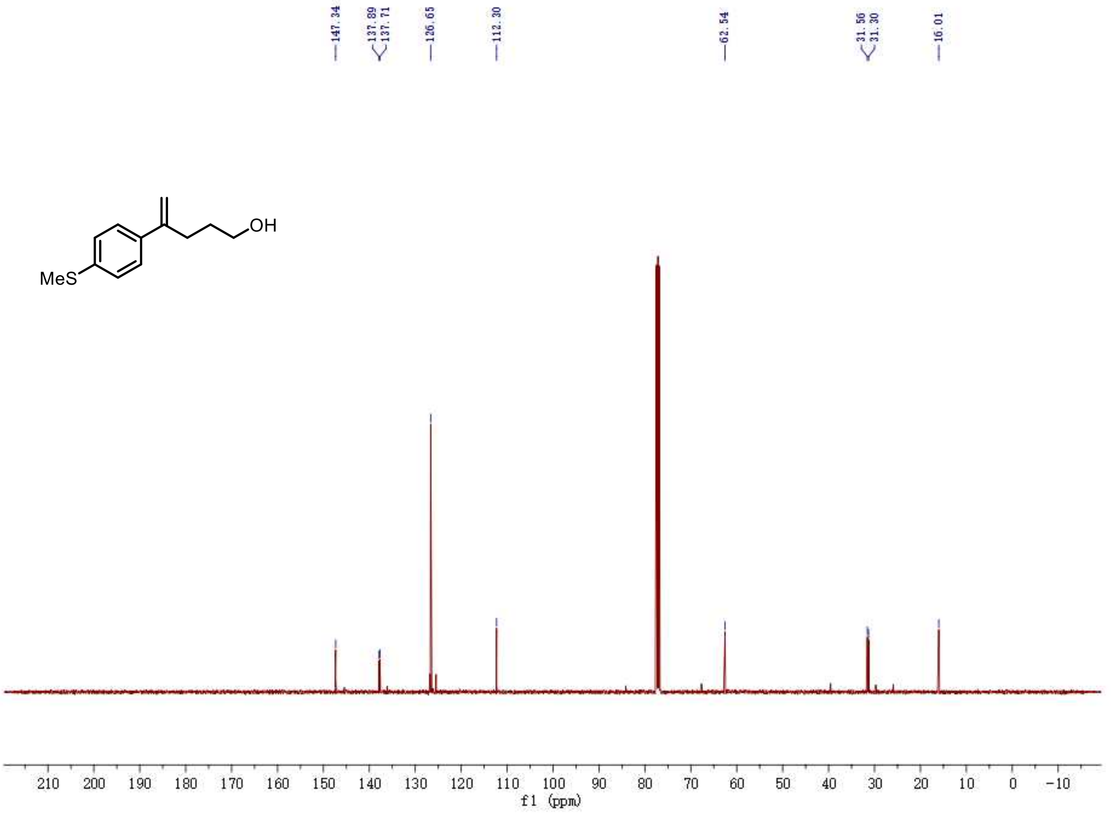

^{1}H-NMR of compound 6a (400 MHz, $CDCl_3$)

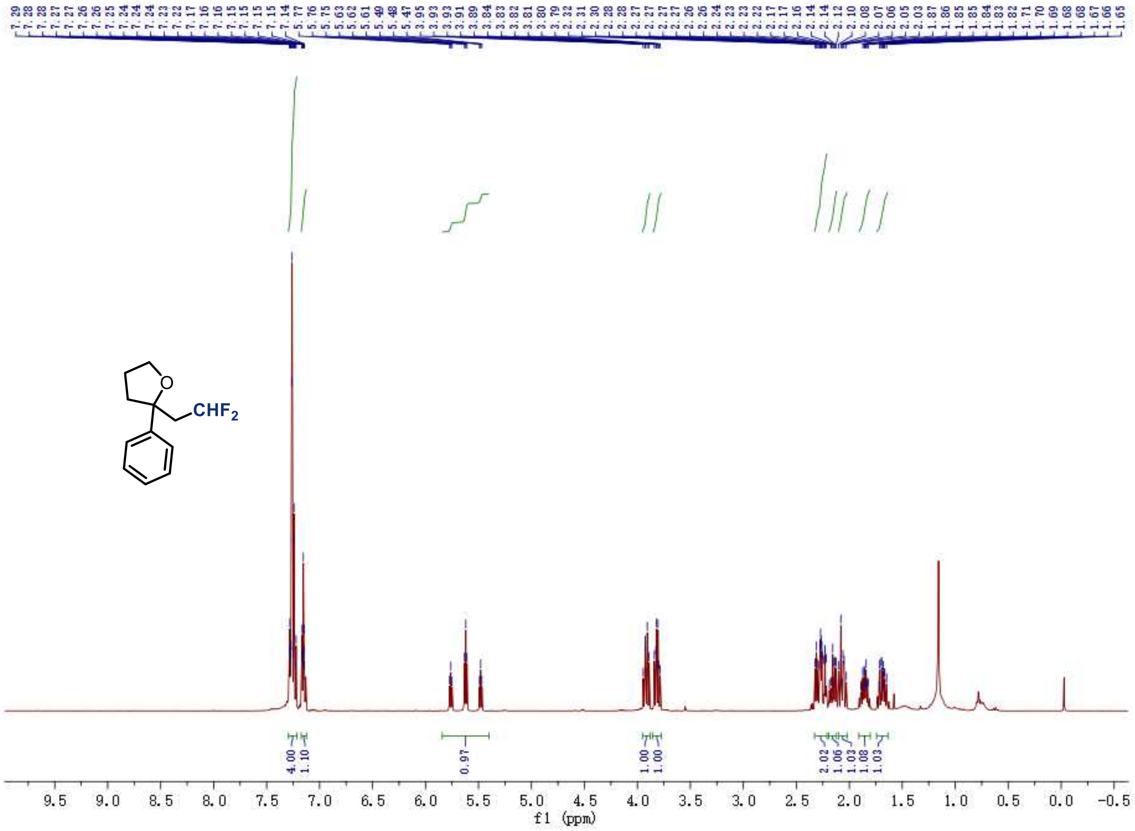

^{13}C-NMR of compound 6a (101 MHz, $CDCl_3$)

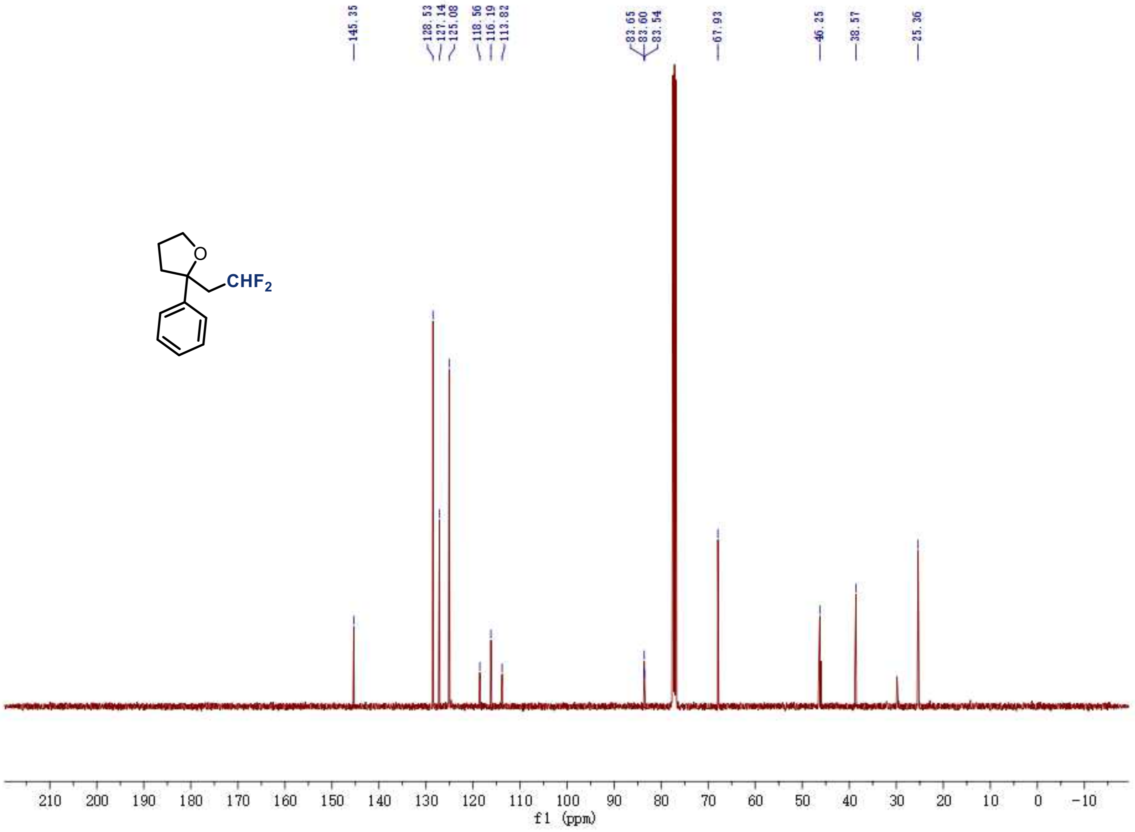

^{19}F-NMR of compound 6a (377 MHz, $CDCl_3$)

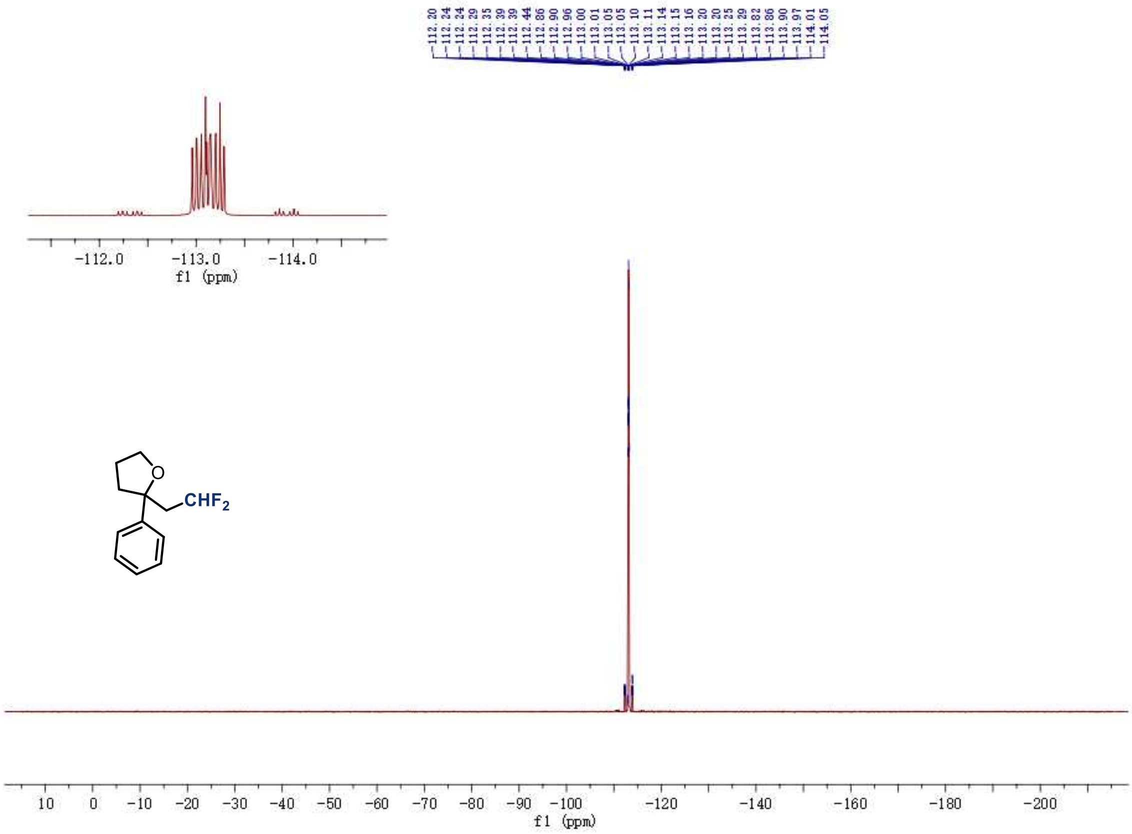

^{1}H-NMR of compound 6b (400 MHz, $CDCl_3$)

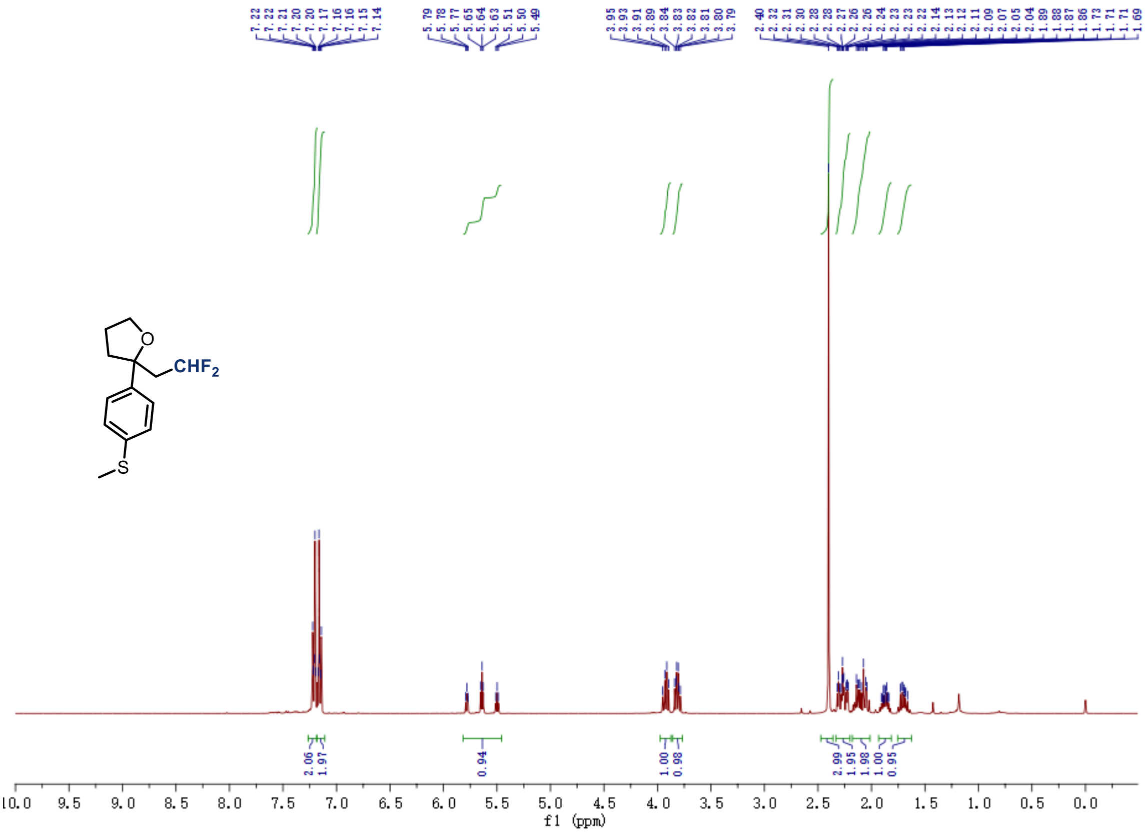

^{13}C-NMR of compound 6b (101 MHz, $CDCl_3$)

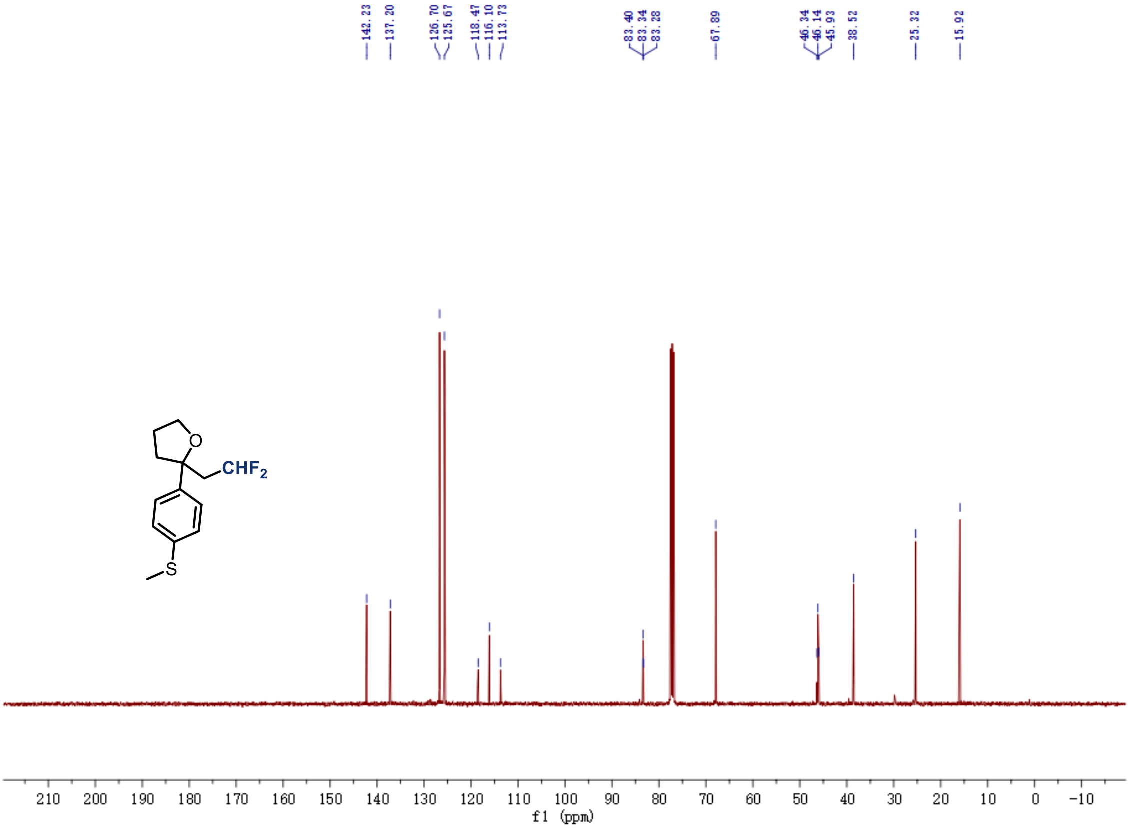

^{19}F-NMR of compound 6b (377 MHz, $CDCl_3$)

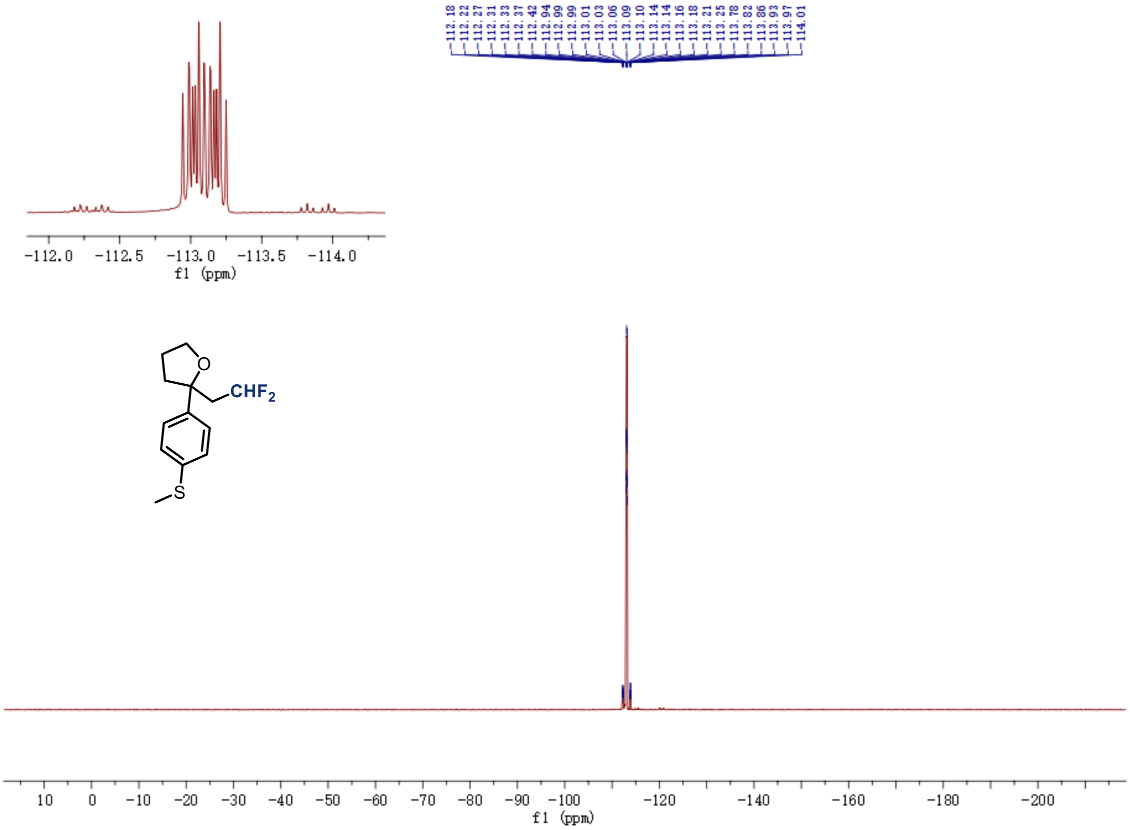

^{1}H-NMR of compound 6c (400 MHz, $CDCl_3$)

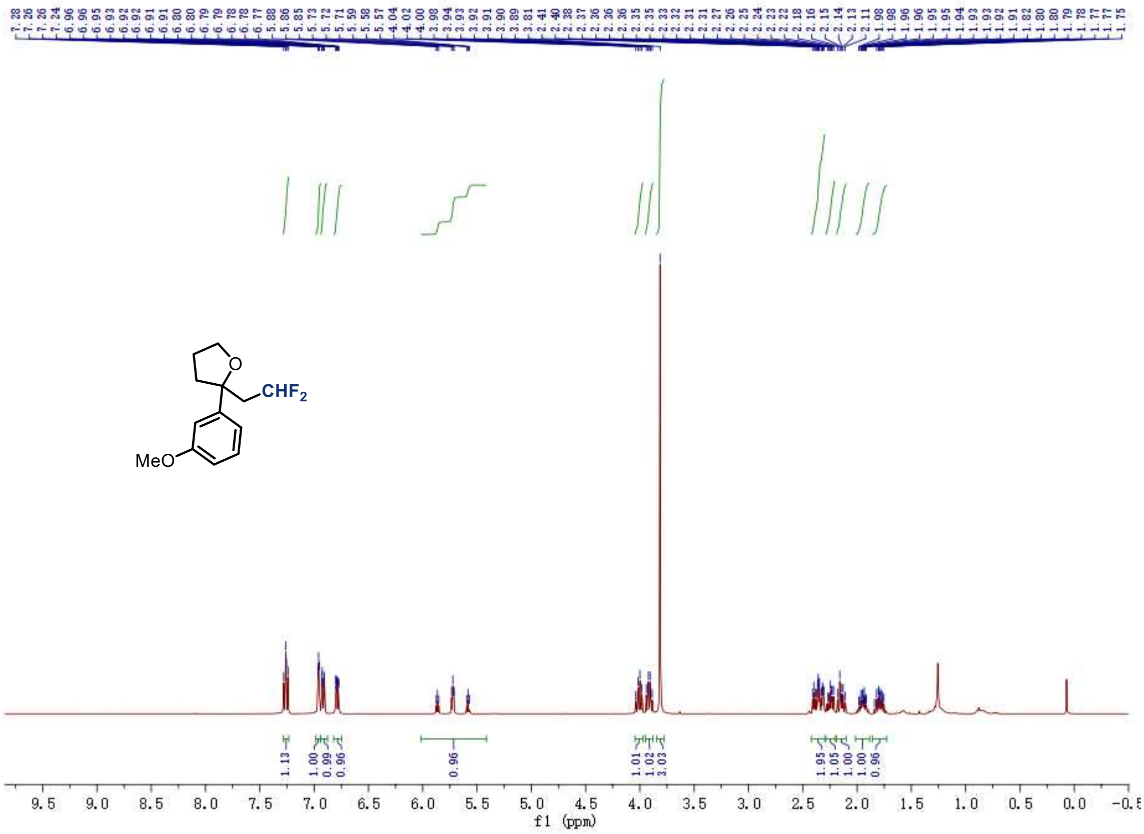

^{13}C-NMR of compound 6c (101 MHz, $CDCl_3$)

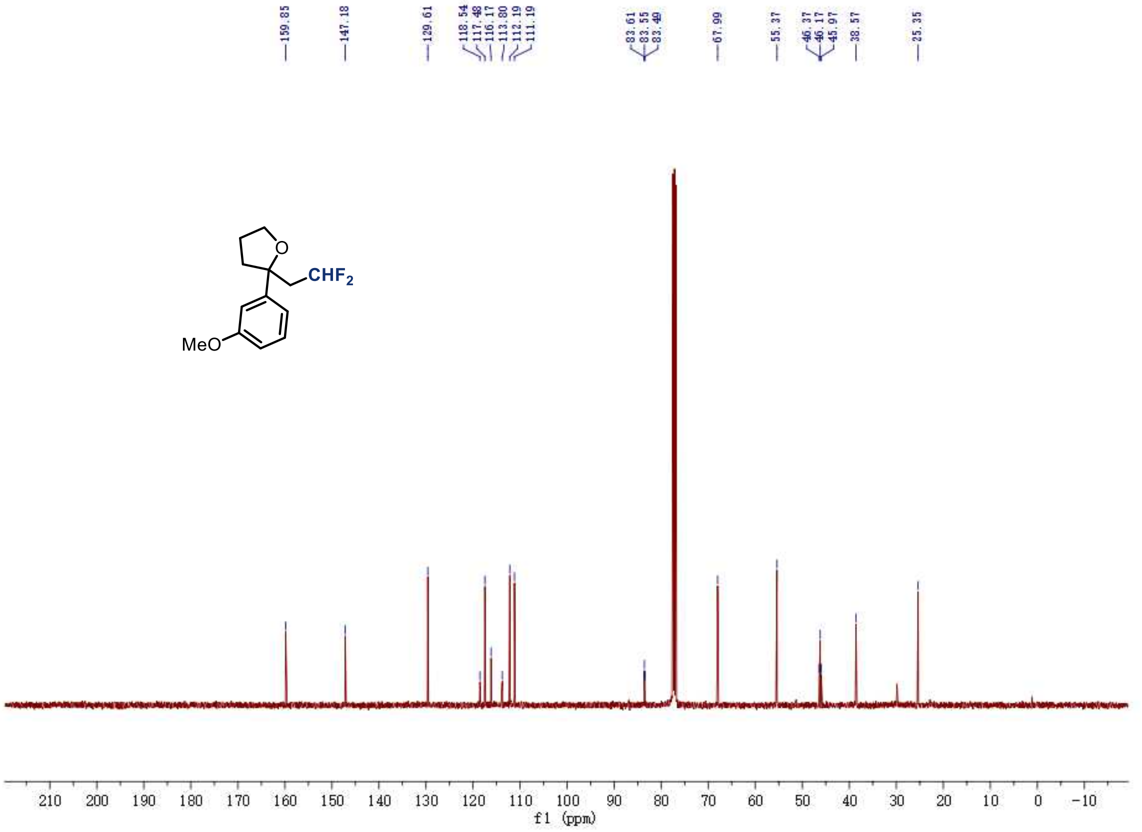

^{19}F-NMR of compound 6c (377 MHz, $CDCl_3$)

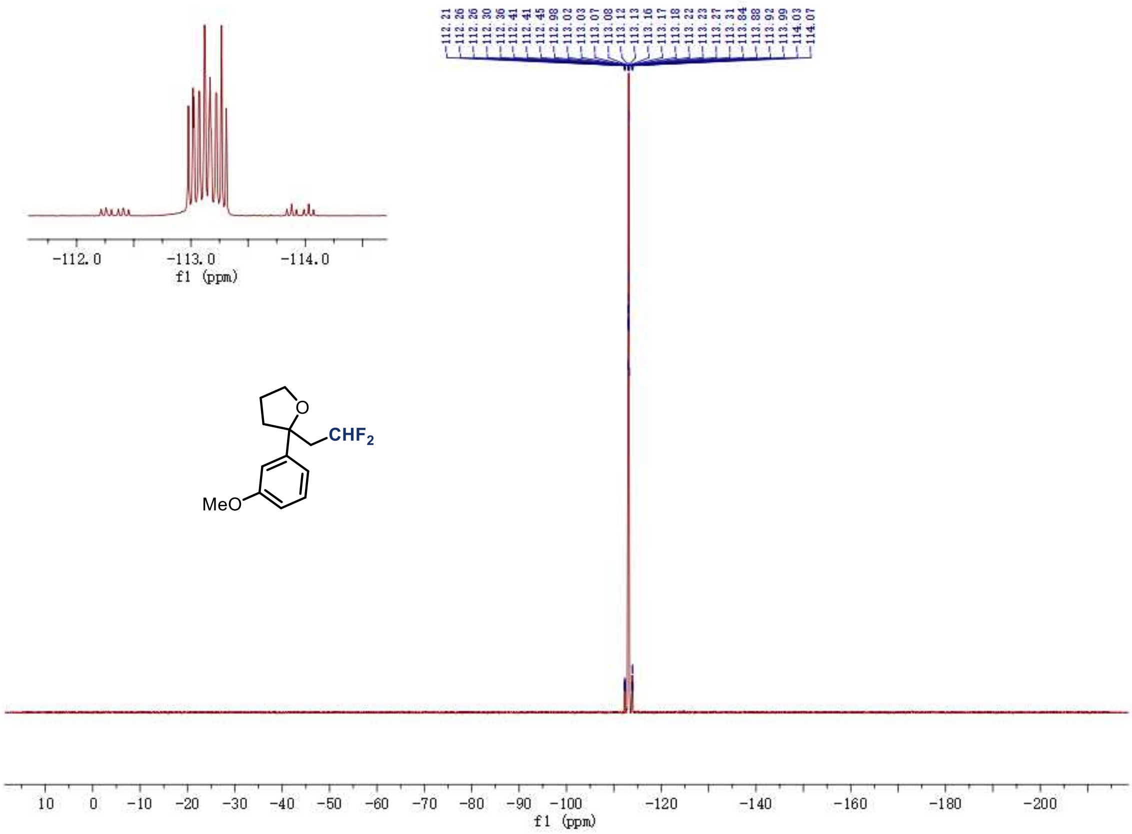

^{1}H-NMR of compound 6d (400 MHz, $CDCl_3$)

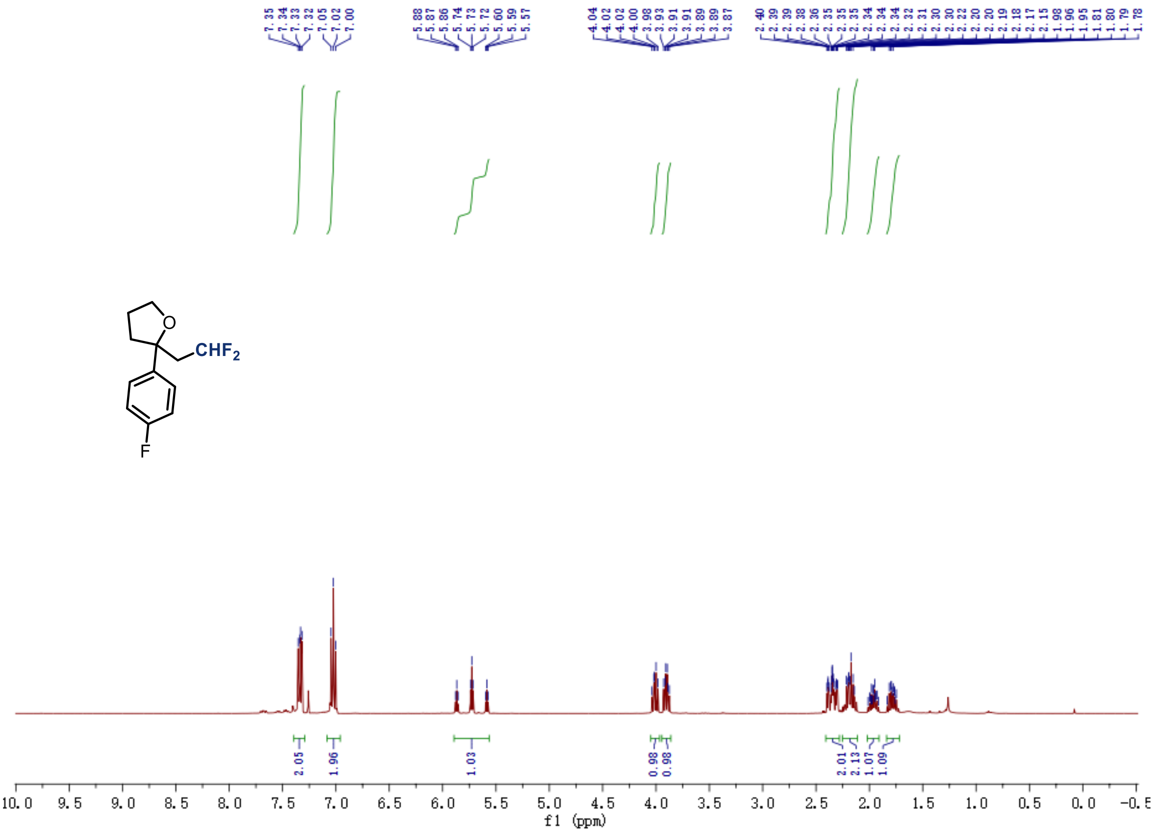

^{13}C-NMR of compound 6d (101 MHz, $CDCl_3$)

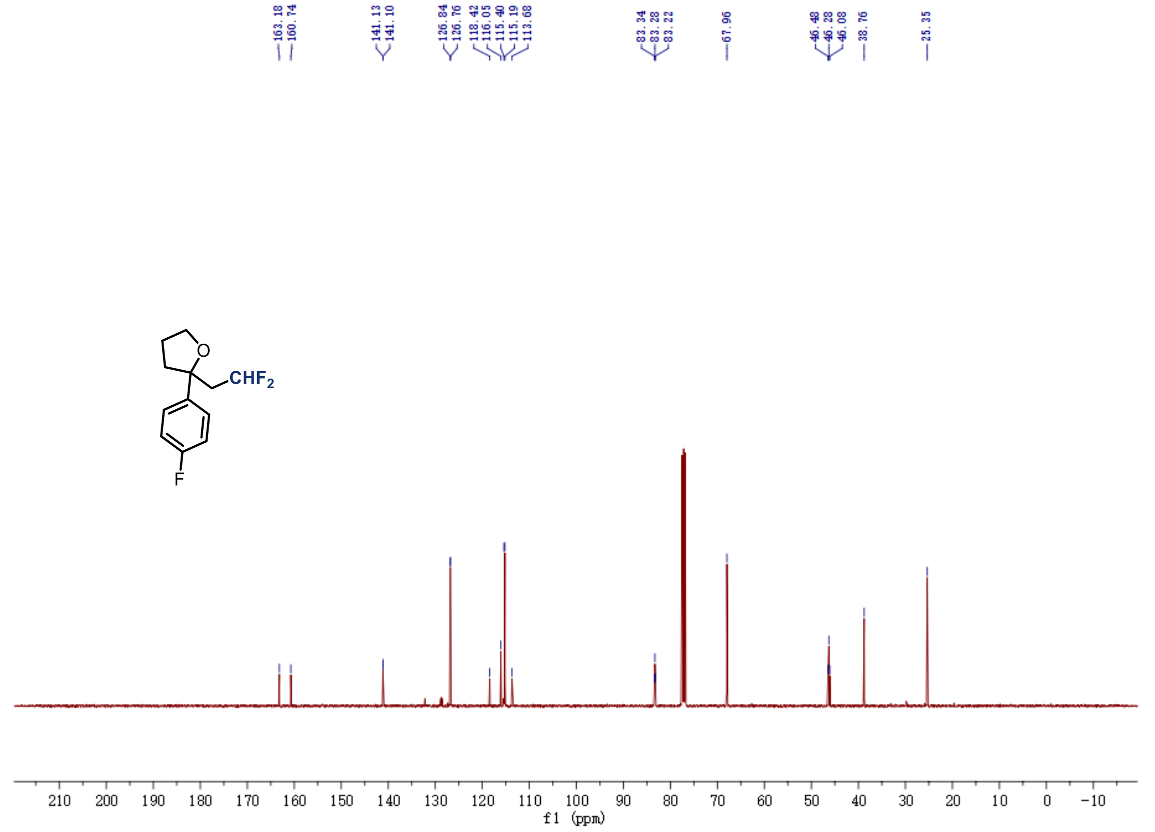

^{19}F-NMR of compound 6d (377 MHz, $CDCl_3$)

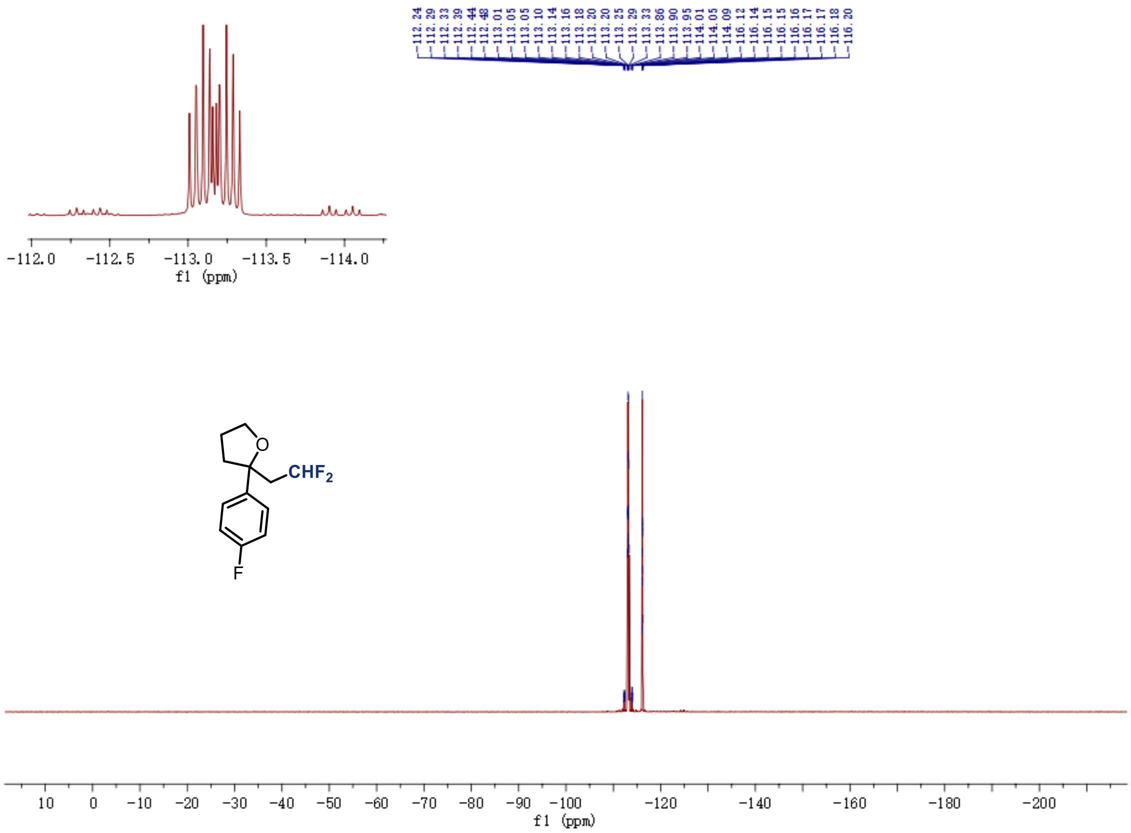

^{1}H-NMR of compound 6e (400 MHz, $CDCl_3$)

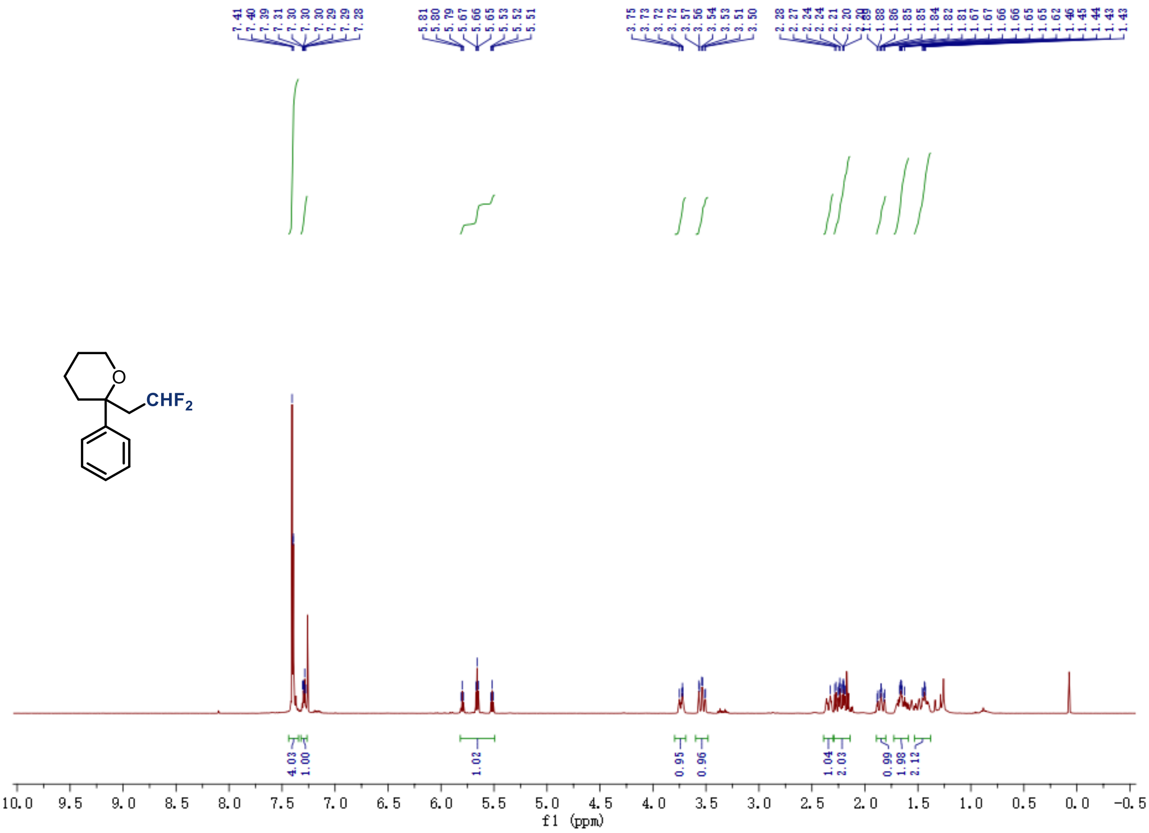

^{13}C-NMR of compound 6e (101 MHz, $CDCl_3$)

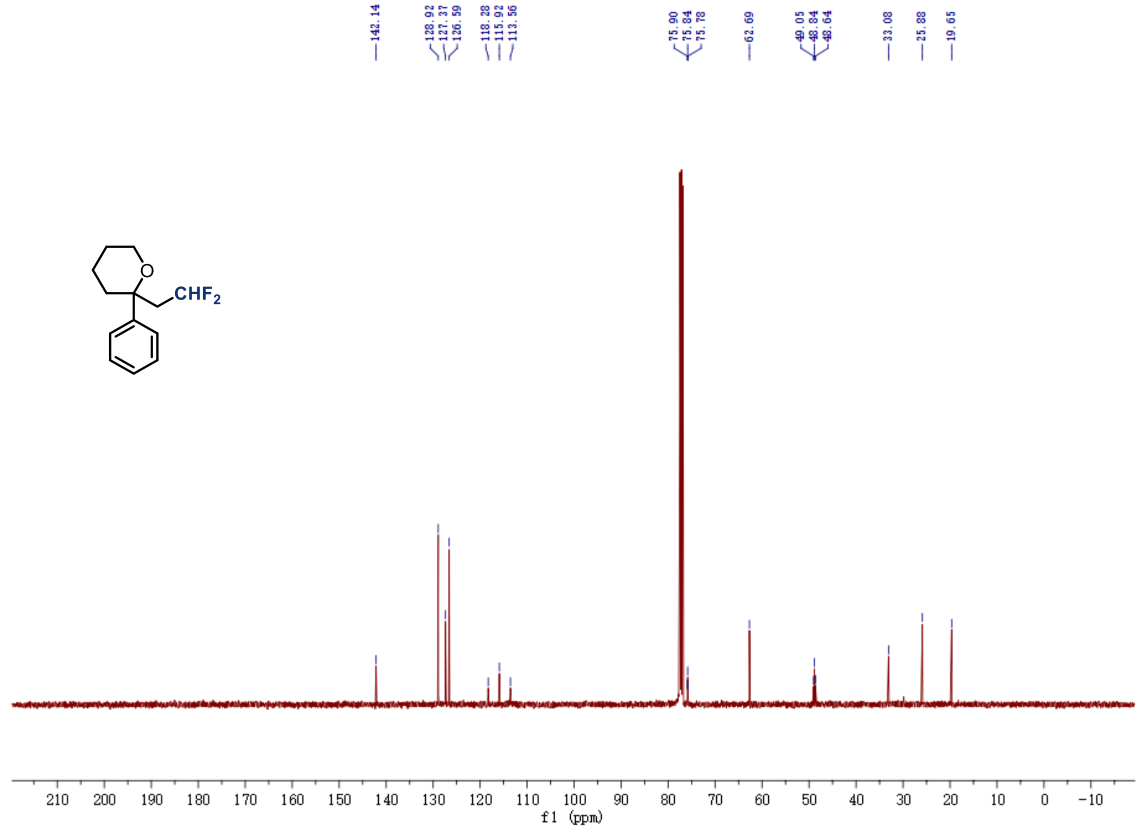

^{19}F-NMR of compound 6e (377 MHz, CDCl$_3$)

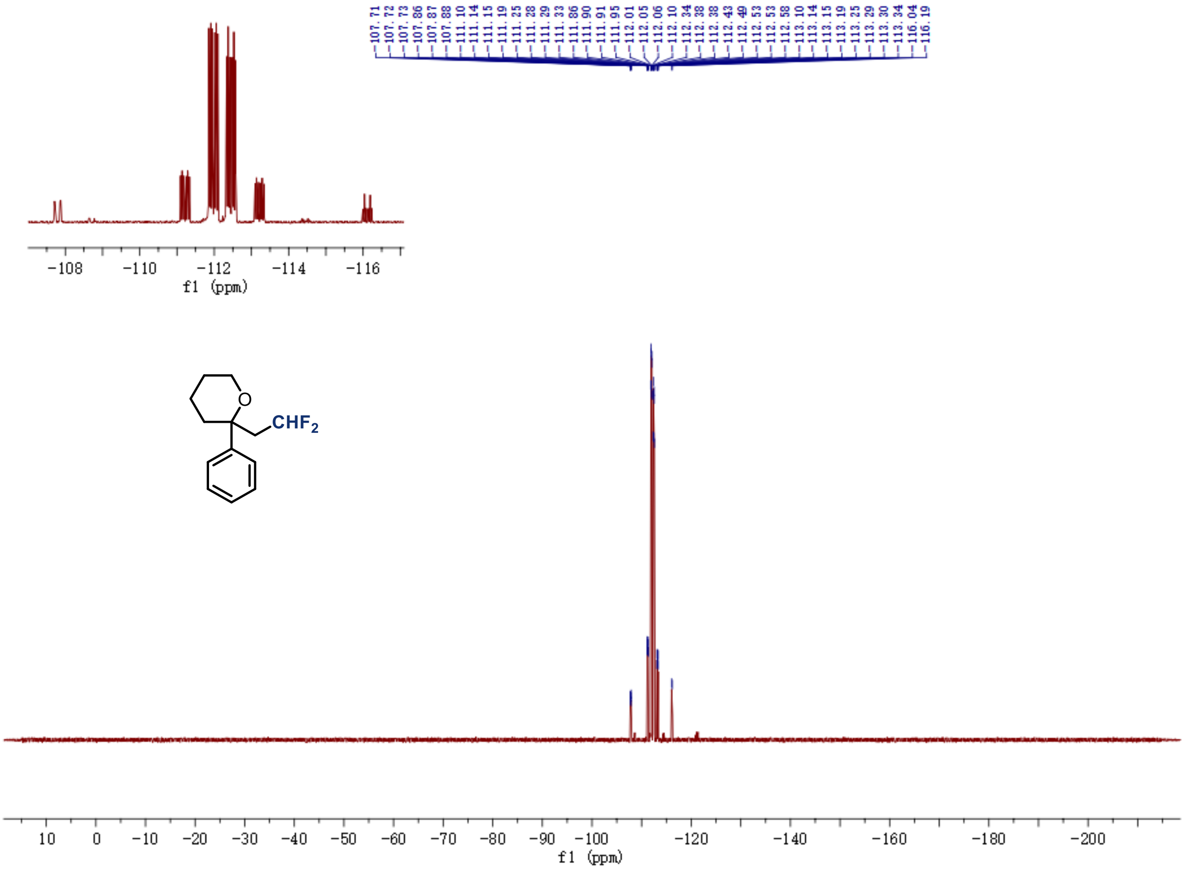

^{1}H-NMR of compound 7c (400 MHz, $CDCl_3$)

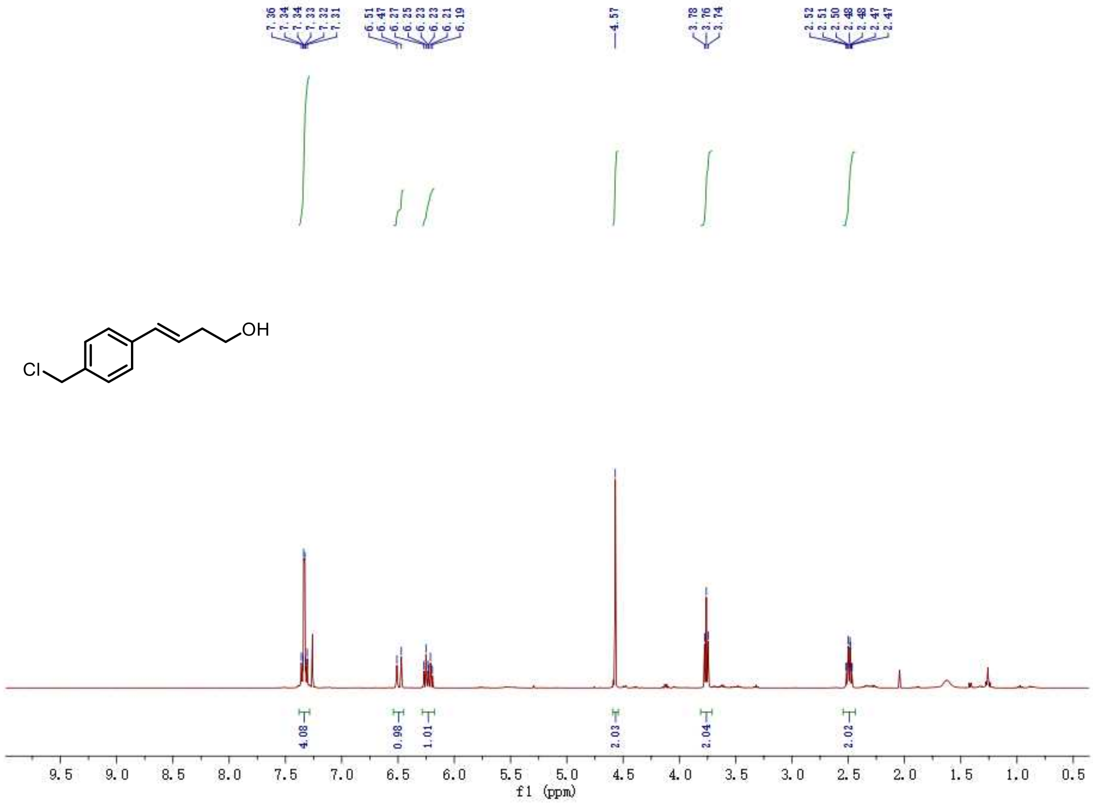

^{13}C-NMR of compound 7c (101 MHz, $CDCl_3$)

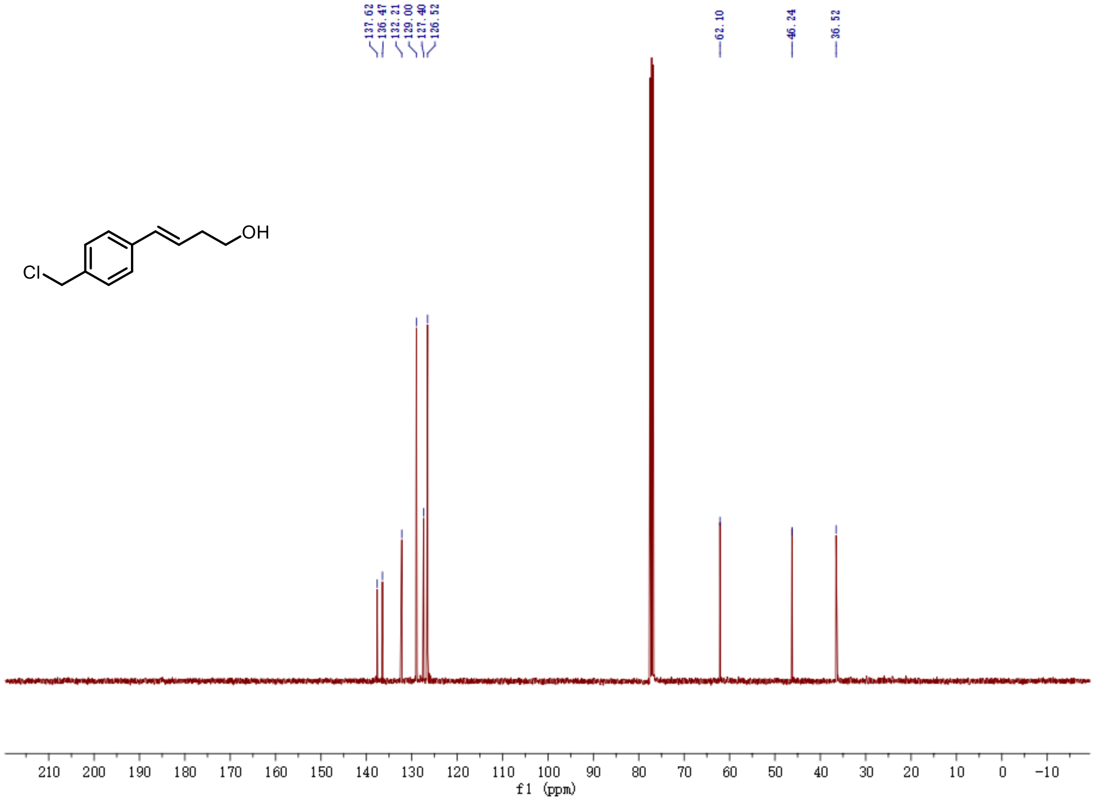

¹H-NMR of compound 7e (400 MHz, $CDCl_3$)

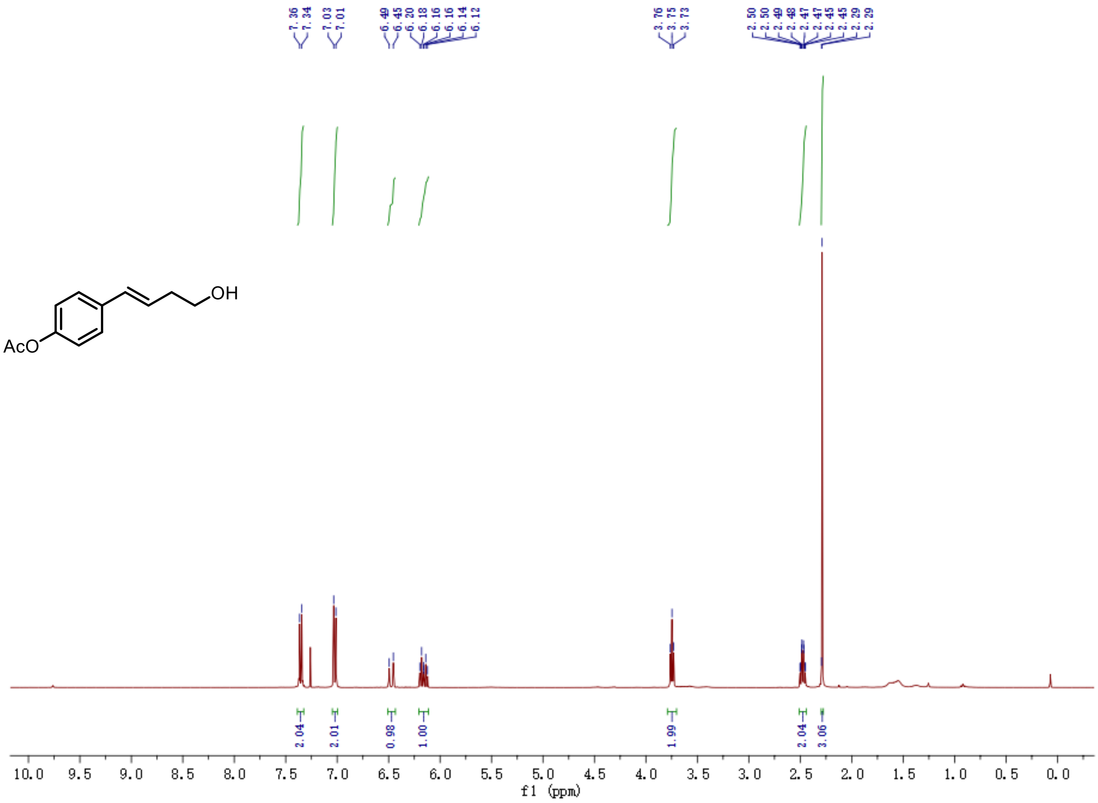

¹³C-NMR of compound 7e (101 MHz, $CDCl_3$)

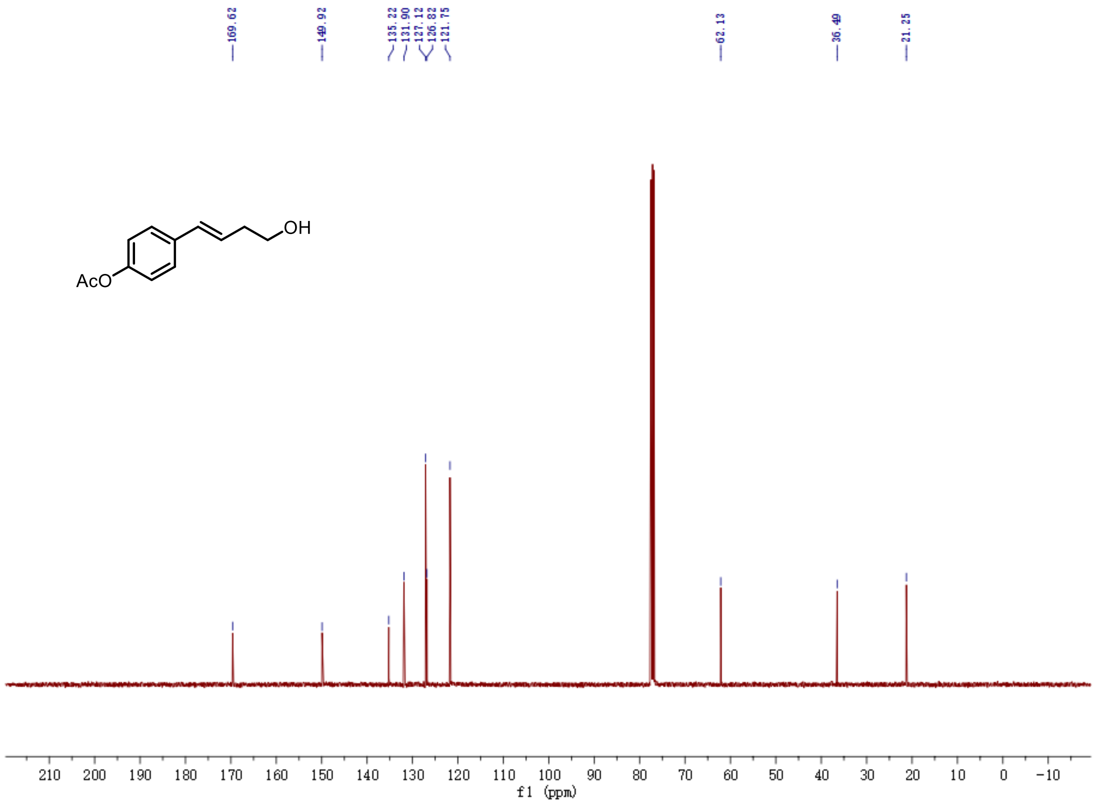

^{1}H-NMR of compound 8a (400 MHz, $CDCl_3$)

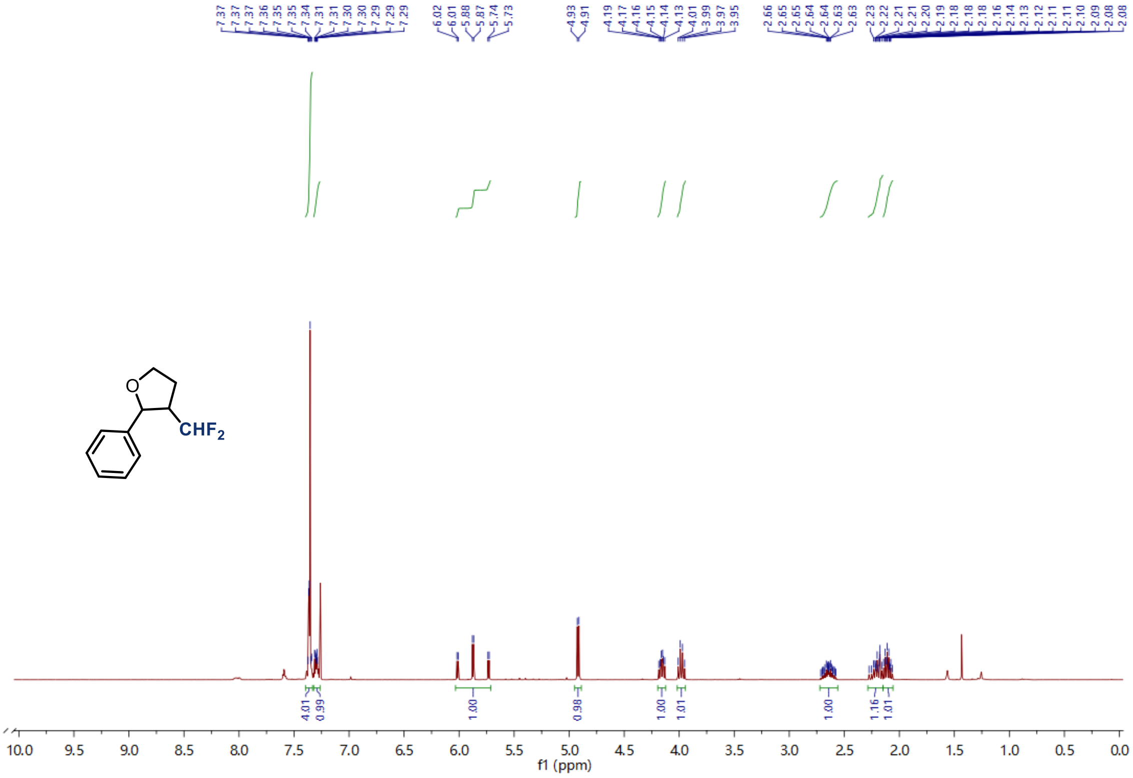

^{13}C-NMR of compound 8a (101 MHz, $CDCl_3$)

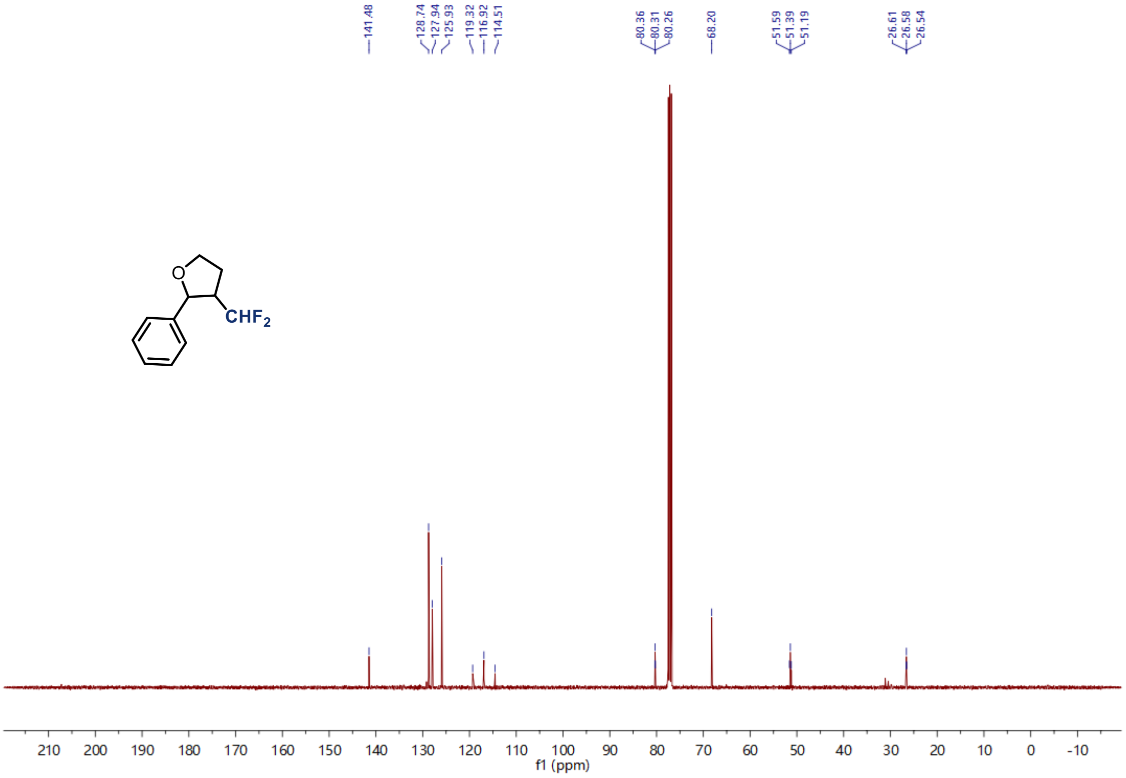

^{19}F-NMR of compound 8a (377 MHz, $CDCl_3$)

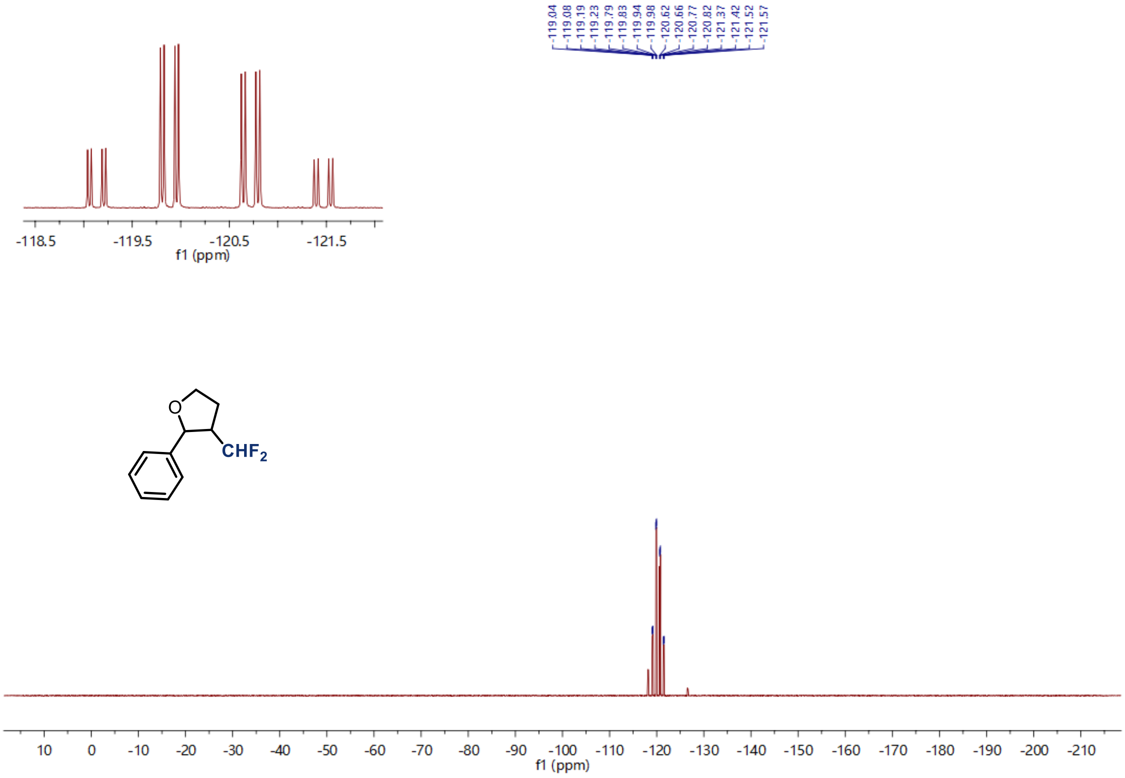

^{1}H-NMR of compound 8b (400 MHz, CDCl$_3$)

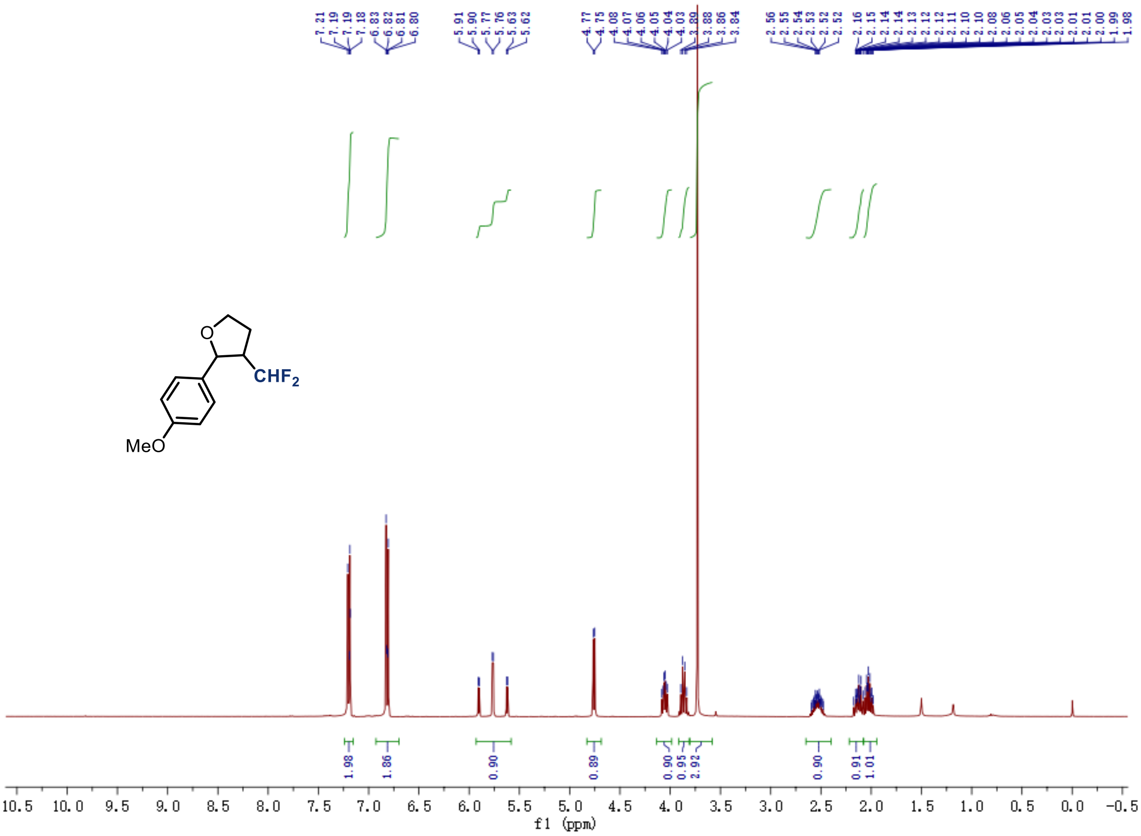

^{13}C-NMR of compound 8b (101 MHz, CDCl$_3$)

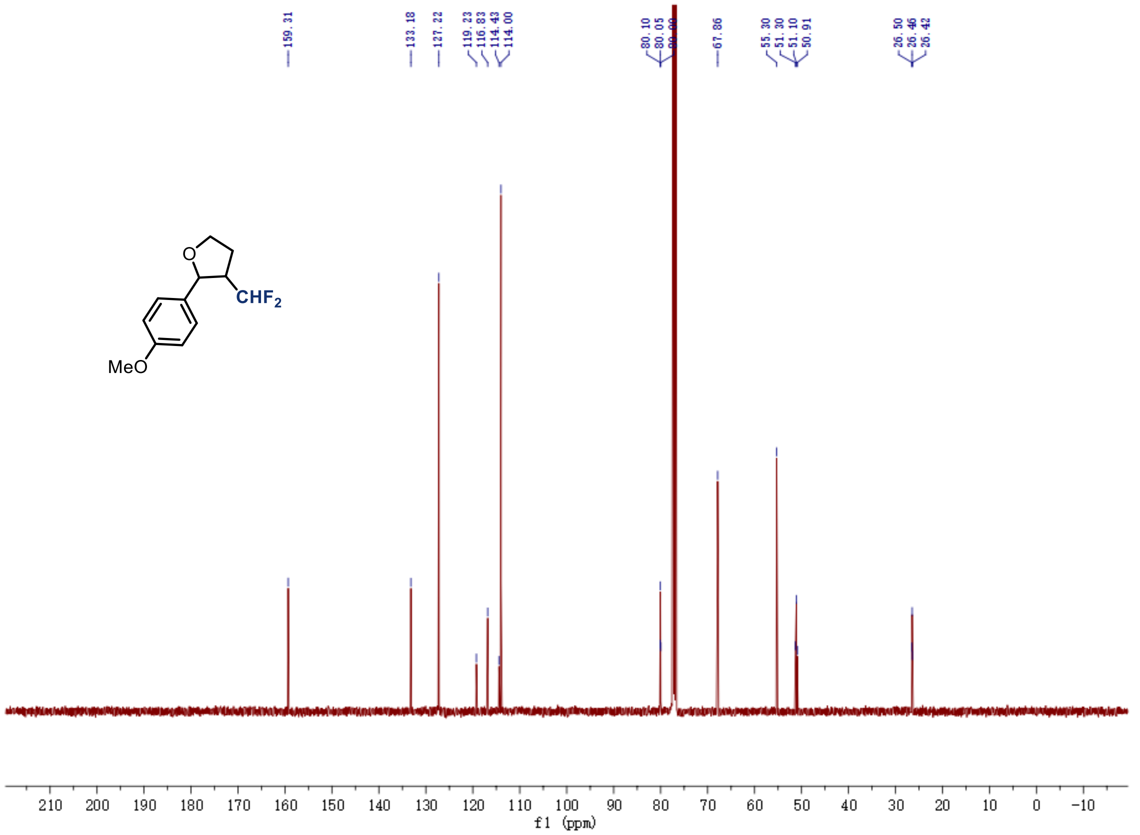

^{19}F-NMR of compound 8b (377 MHz, $CDCl_3$)

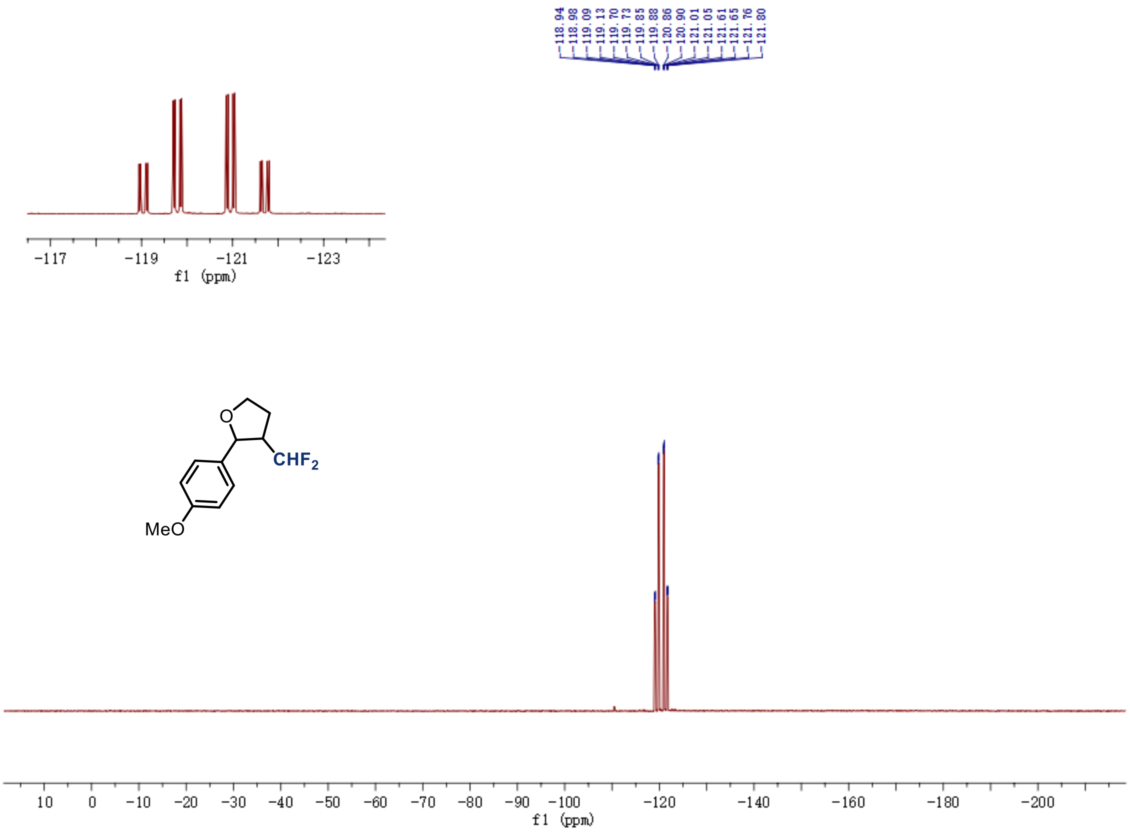

^{1}H-NMR of compound 8c (400 MHz, $CDCl_3$)

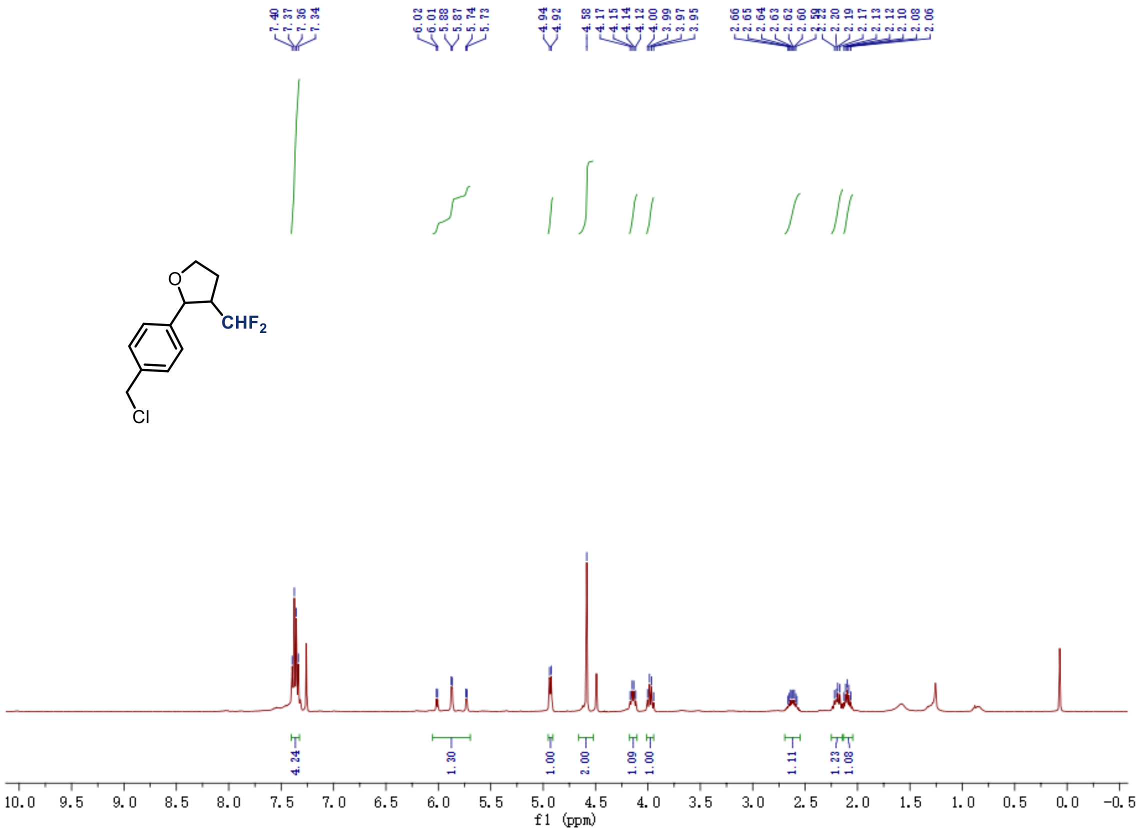

^{13}C-NMR of compound 8c (101 MHz, $CDCl_3$)

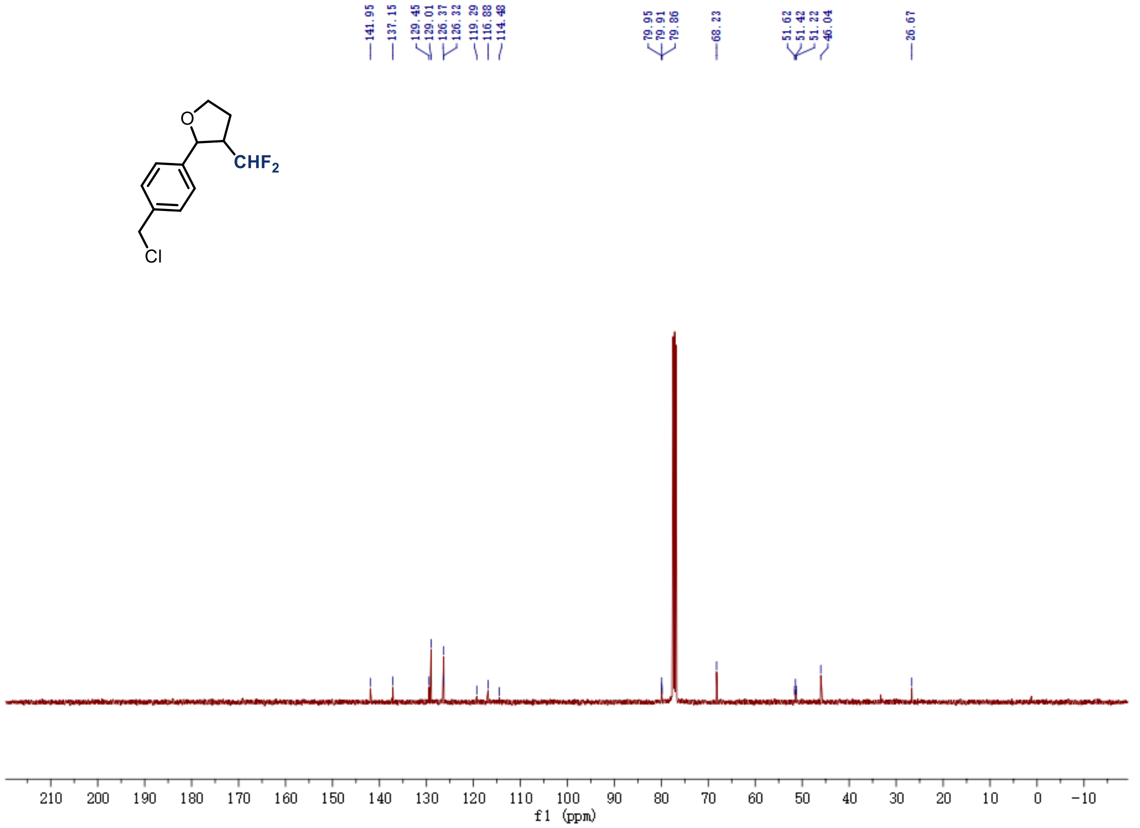

^{19}F-NMR of compound 8c (377 MHz, CDCl$_3$)

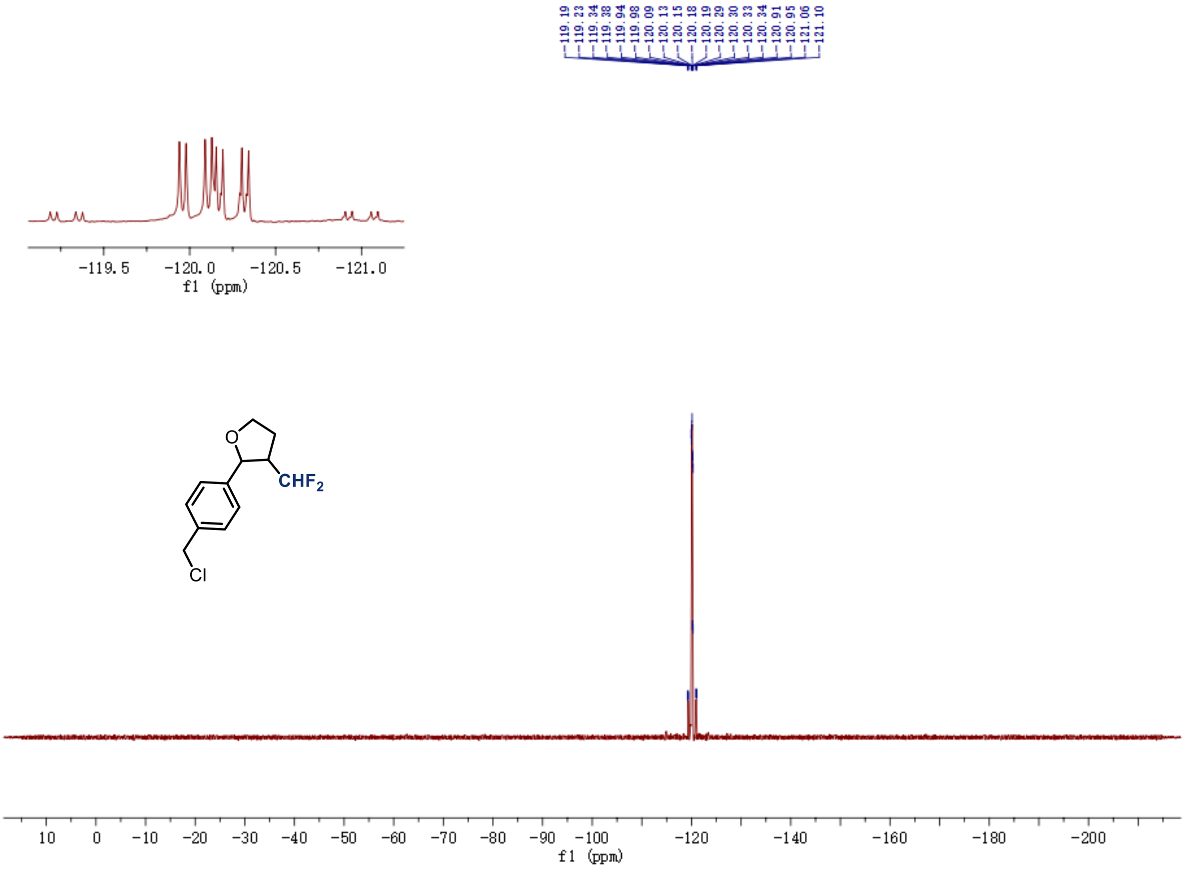

^{1}H-NMR of compound 8d (400 MHz, $CDCl_3$)

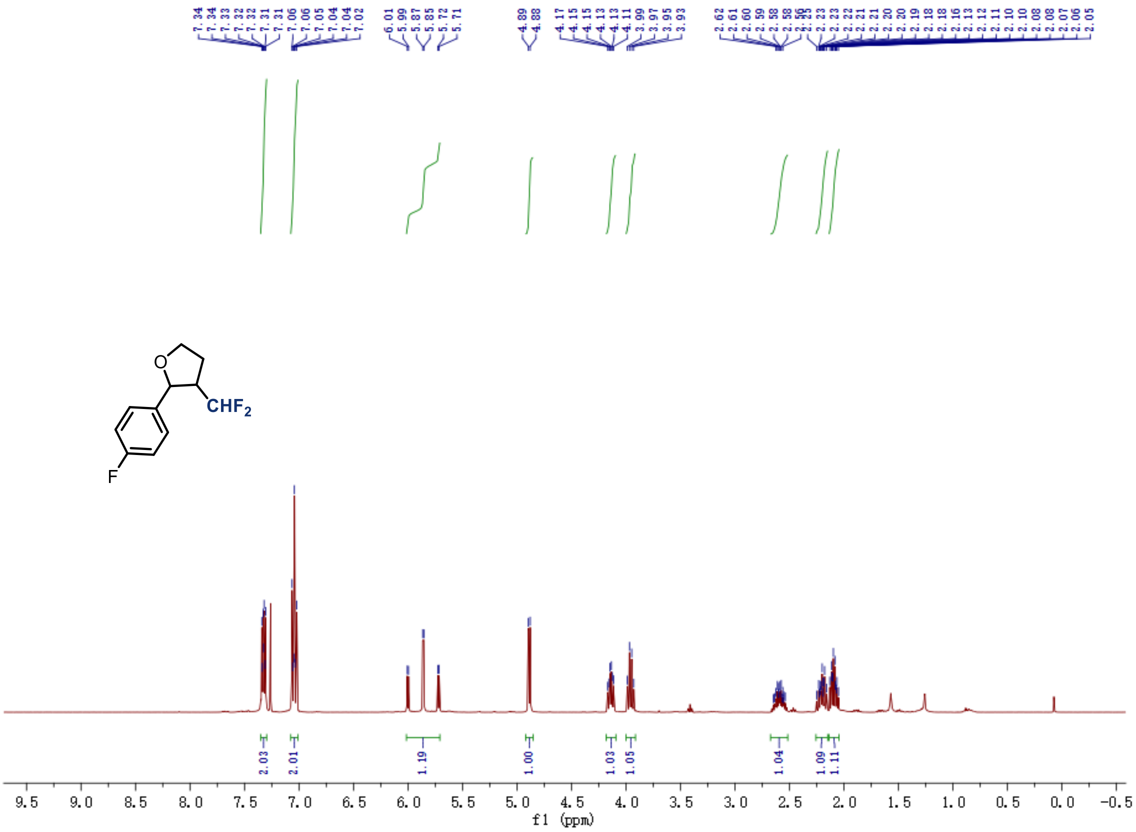

^{13}C-NMR of compound 8d (101 MHz, $CDCl_3$)

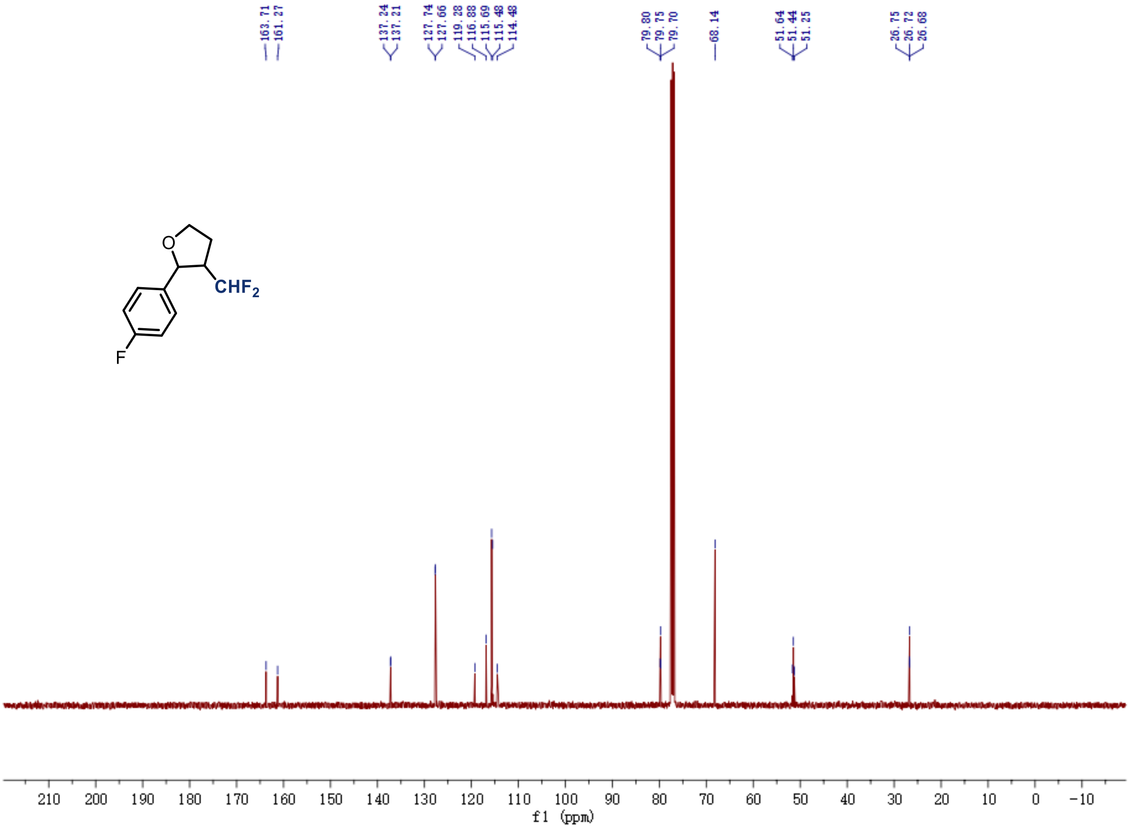

^{19}F-NMR of compound 8d (377 MHz, $CDCl_3$)

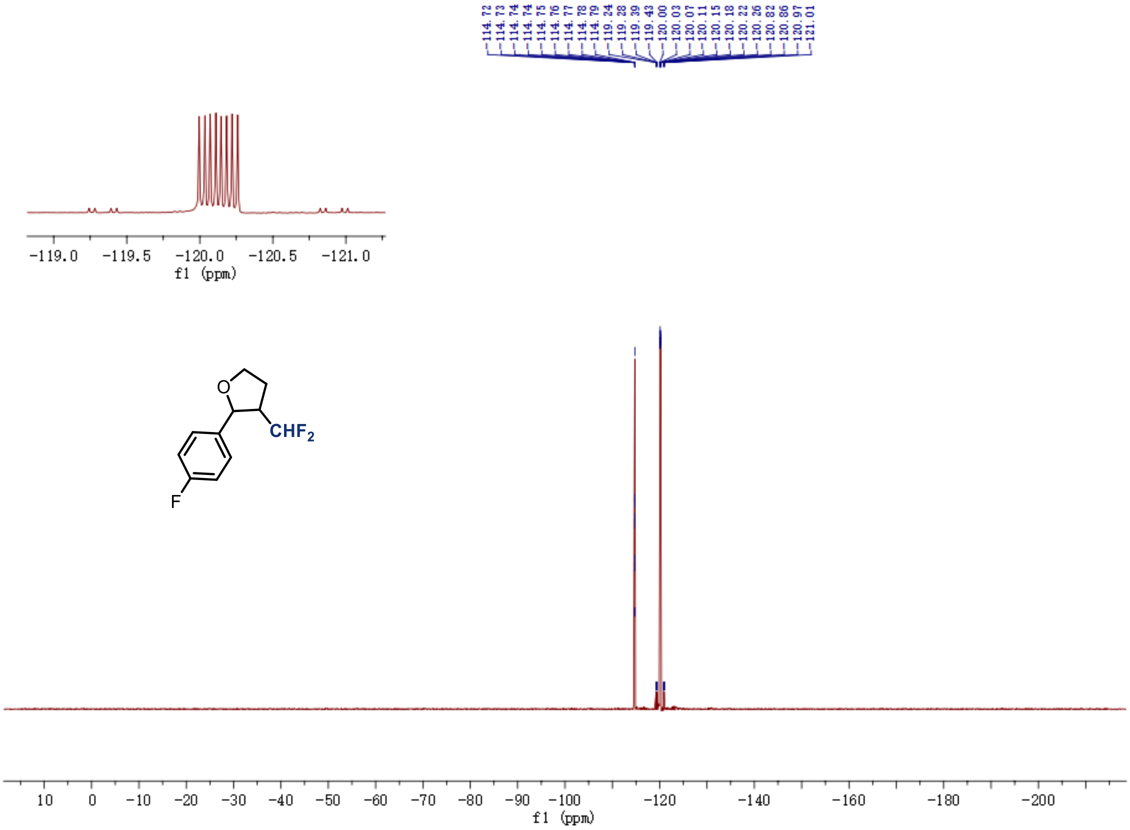

^{1}H-NMR of compound 8e (400 MHz, $CDCl_3$)

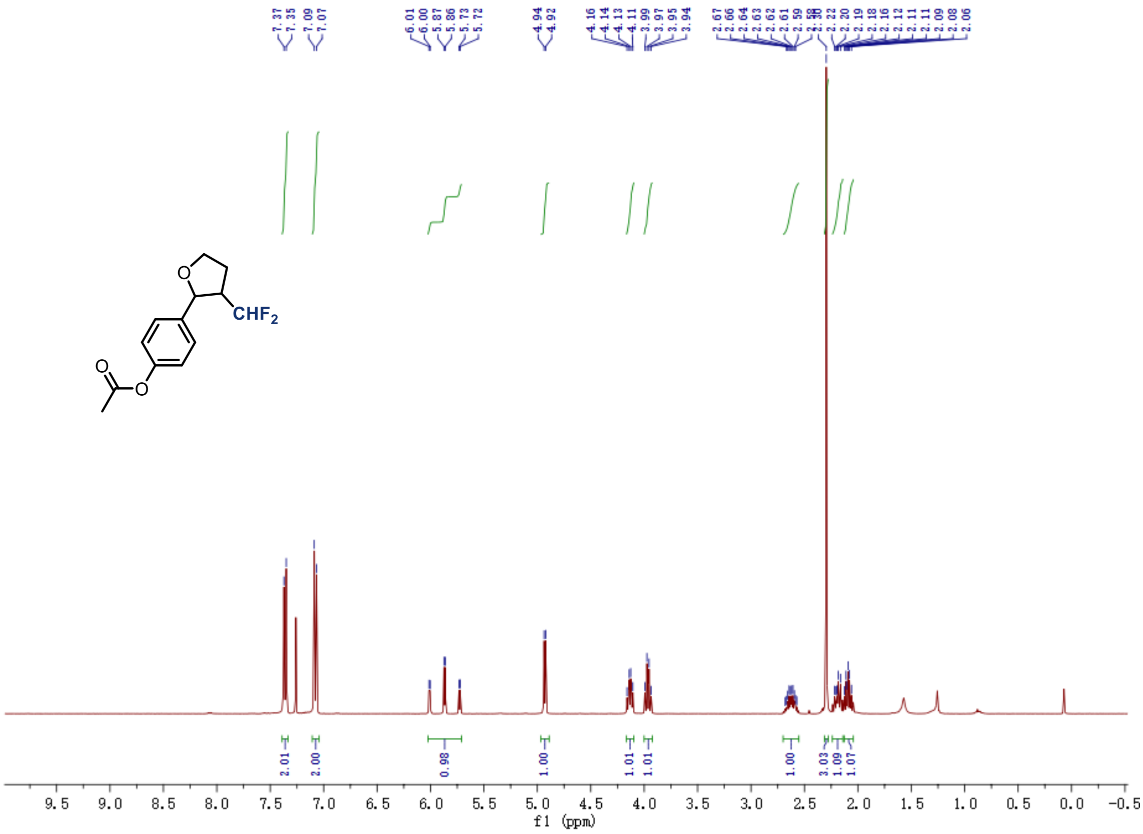

^{13}C-NMR of compound 8e (101 MHz, $CDCl_3$)

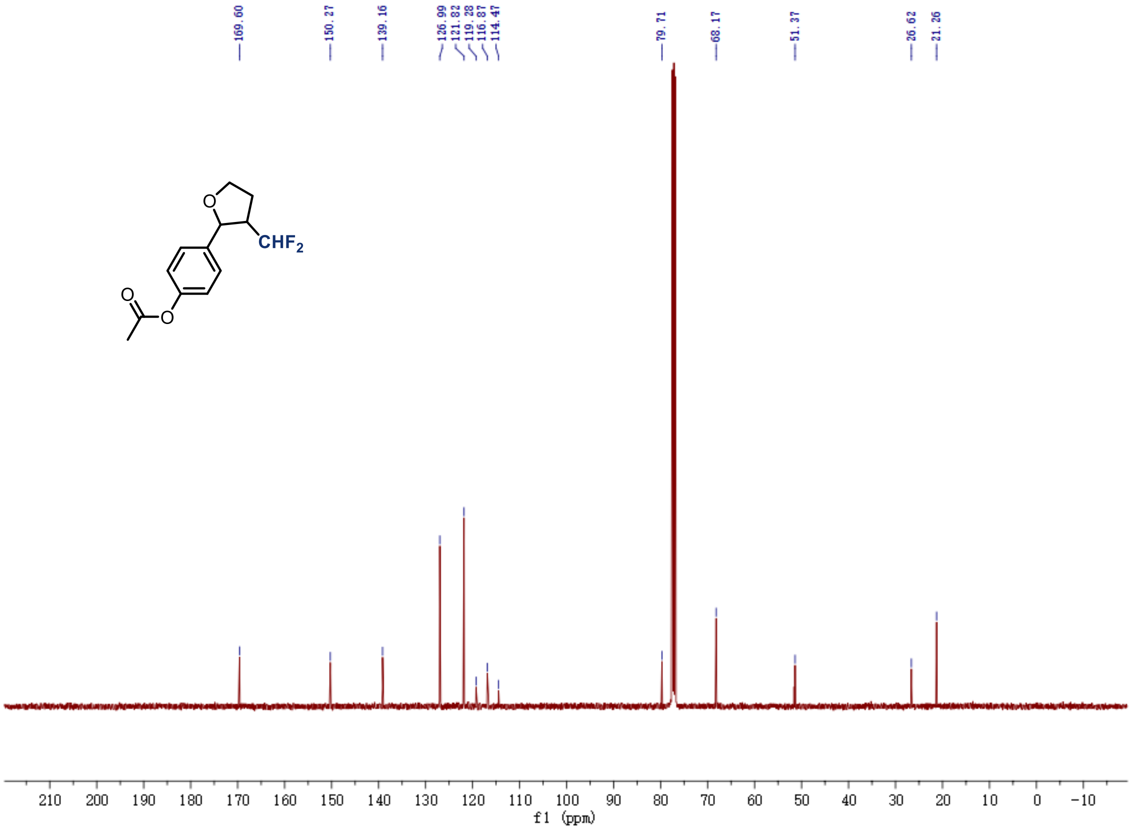

^{19}F-NMR of compound 8e (377 MHz, $CDCl_3$)

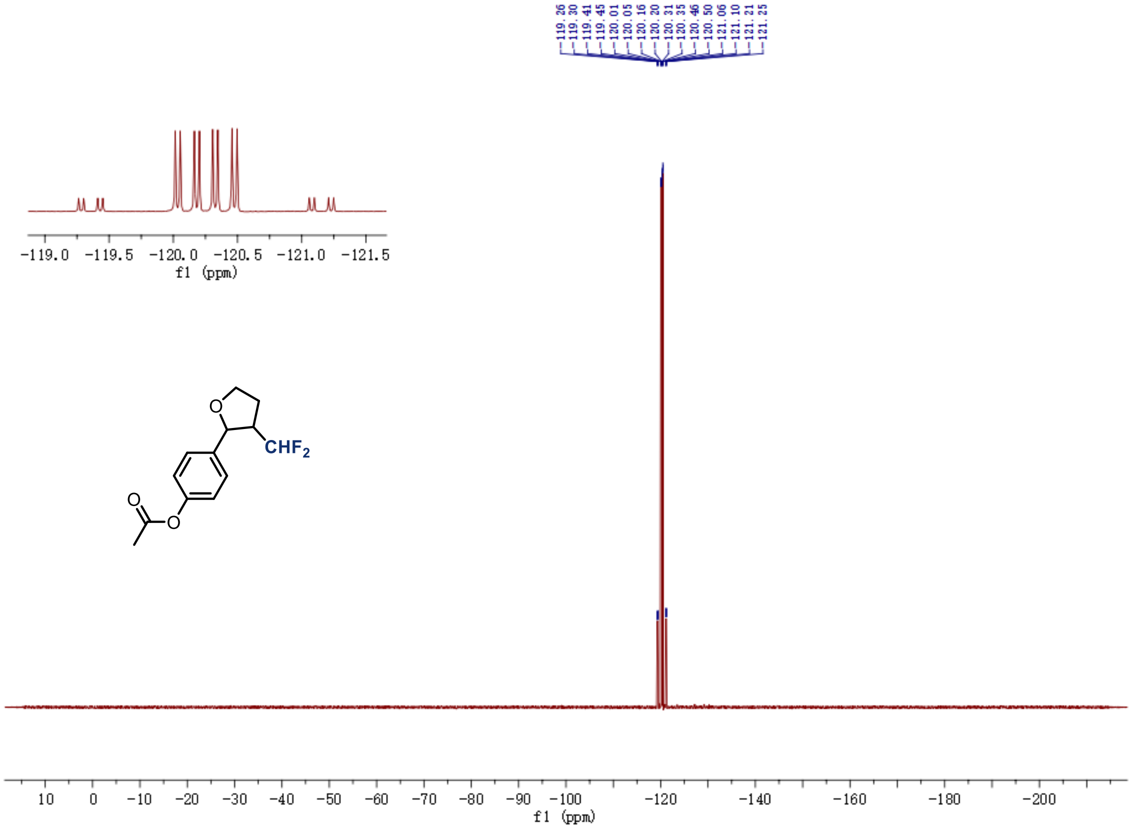

^{1}H-NMR of compound 10a (400 MHz, $CDCl_3$)

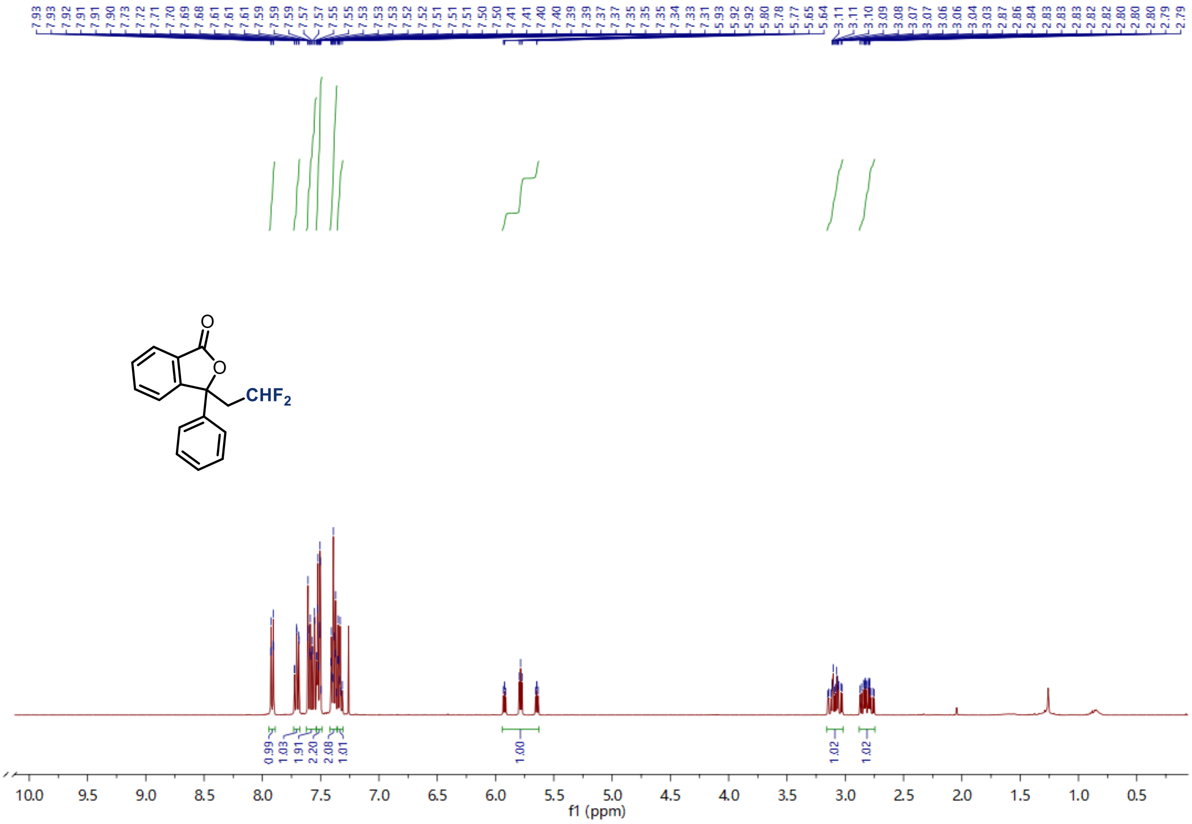

^{13}C-NMR of compound 10a (101 MHz, $CDCl_3$)

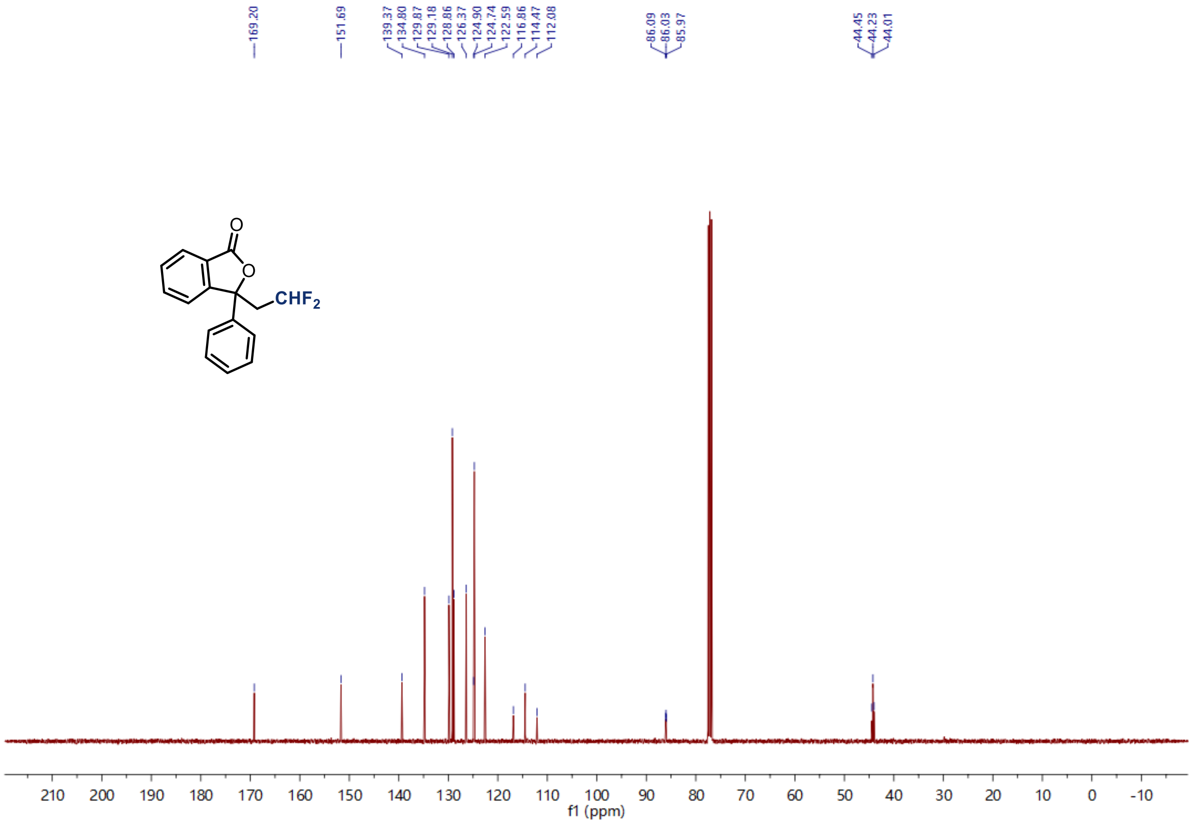

^{19}F-NMR of compound 10a (377 MHz, $CDCl_3$)

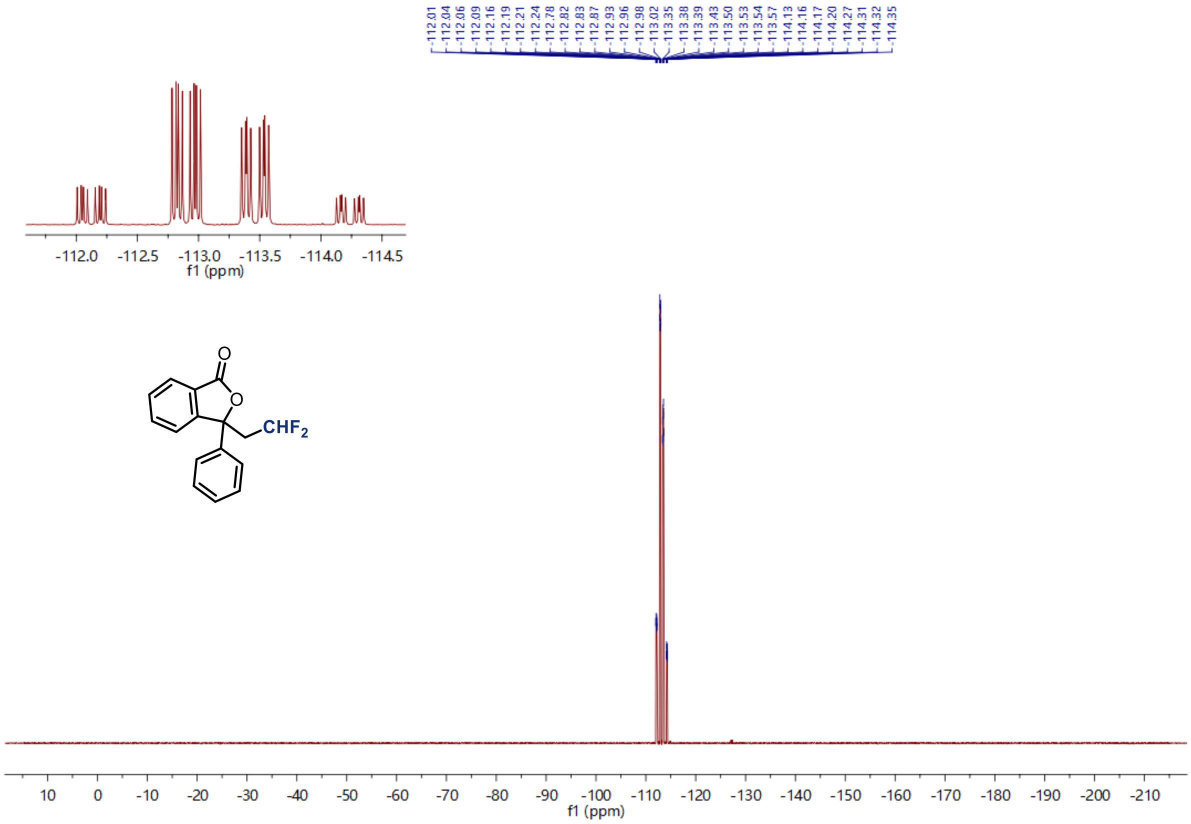

^{1}H-NMR of compound 10b (400 MHz, $CDCl_3$)

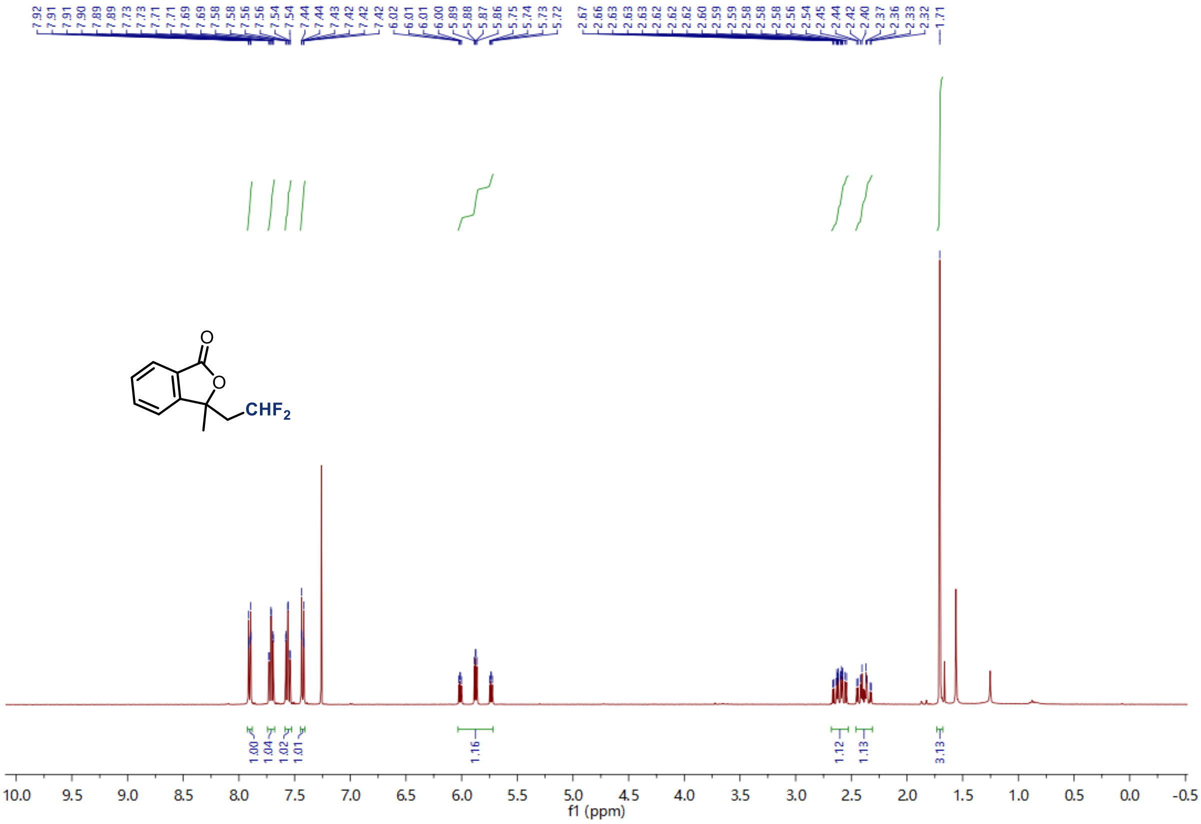

^{13}C-NMR of compound 10b (101 MHz, $CDCl_3$)

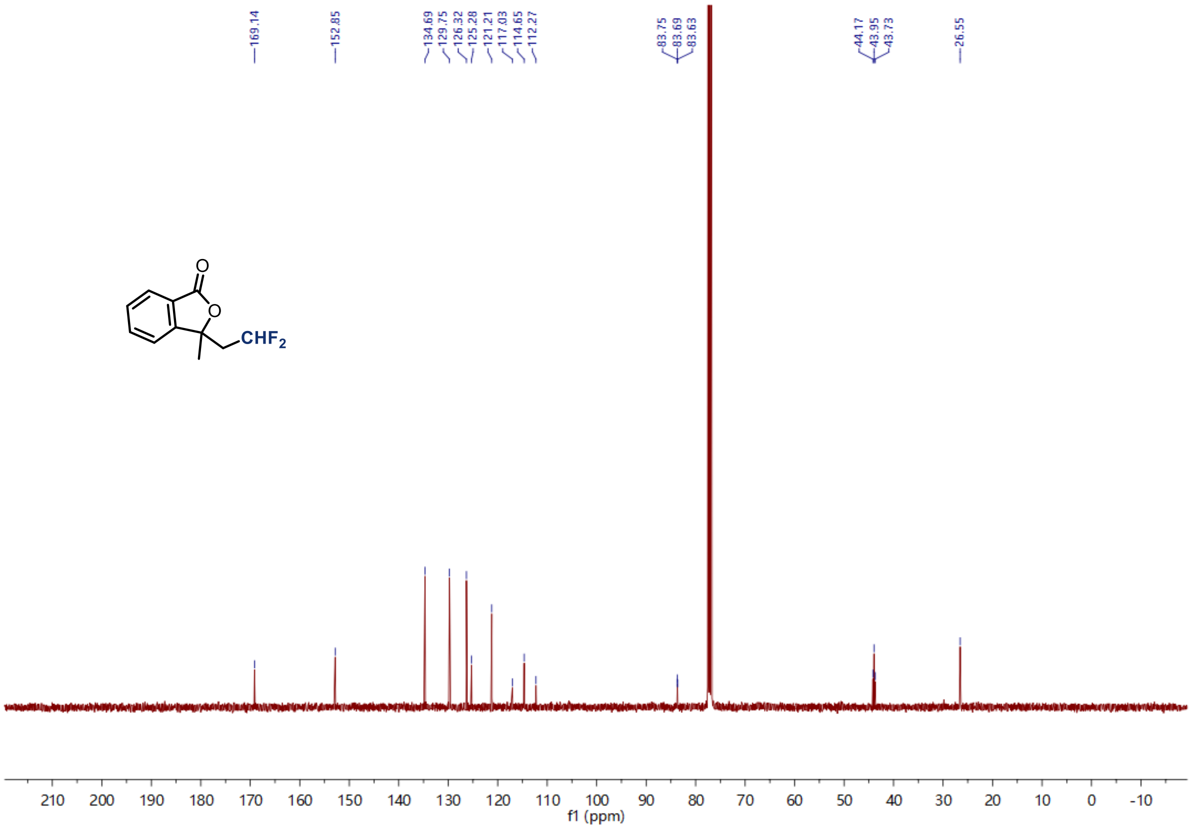

^{19}F-NMR of compound 10b (377 MHz, $CDCl_3$)

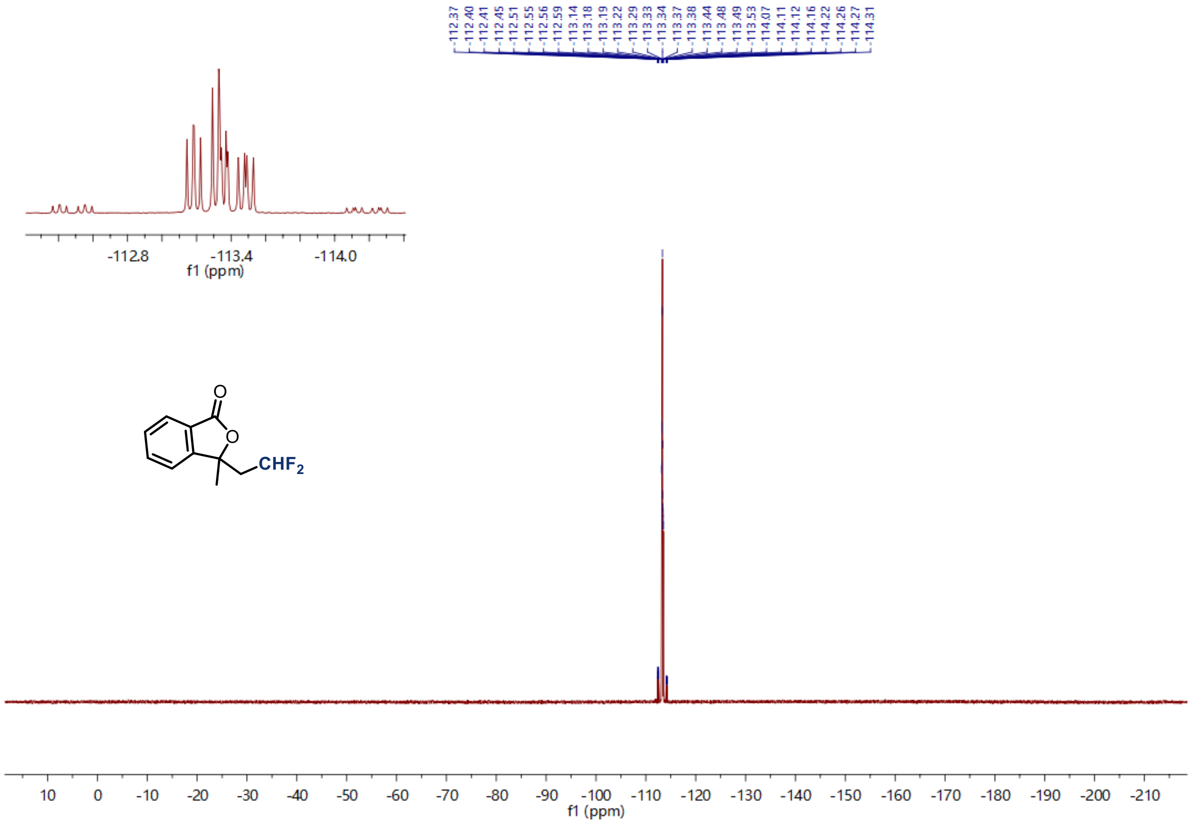

^{1}H-NMR of compound 12 (400 MHz, $CDCl_3$)

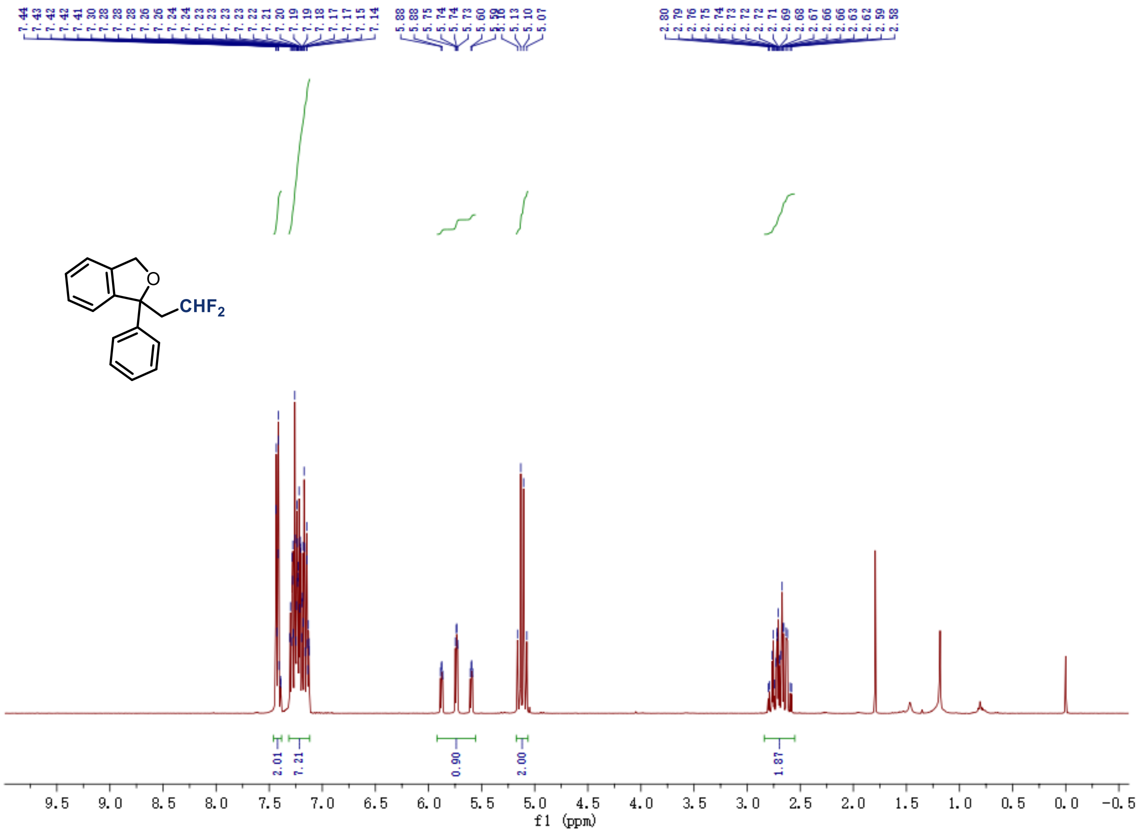

^{13}C-NMR of compound 12 (101 MHz, $CDCl_3$)

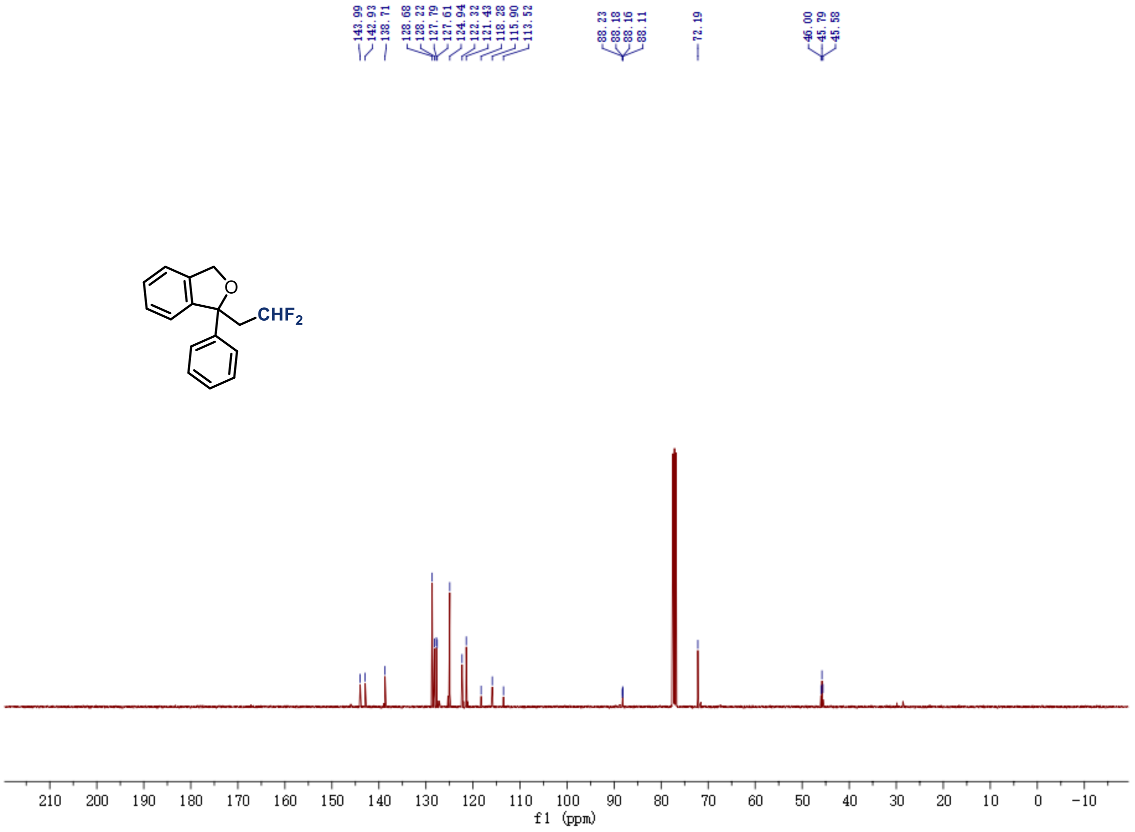

^{19}F-NMR of compound 12 (377 MHz, $CDCl_3$)

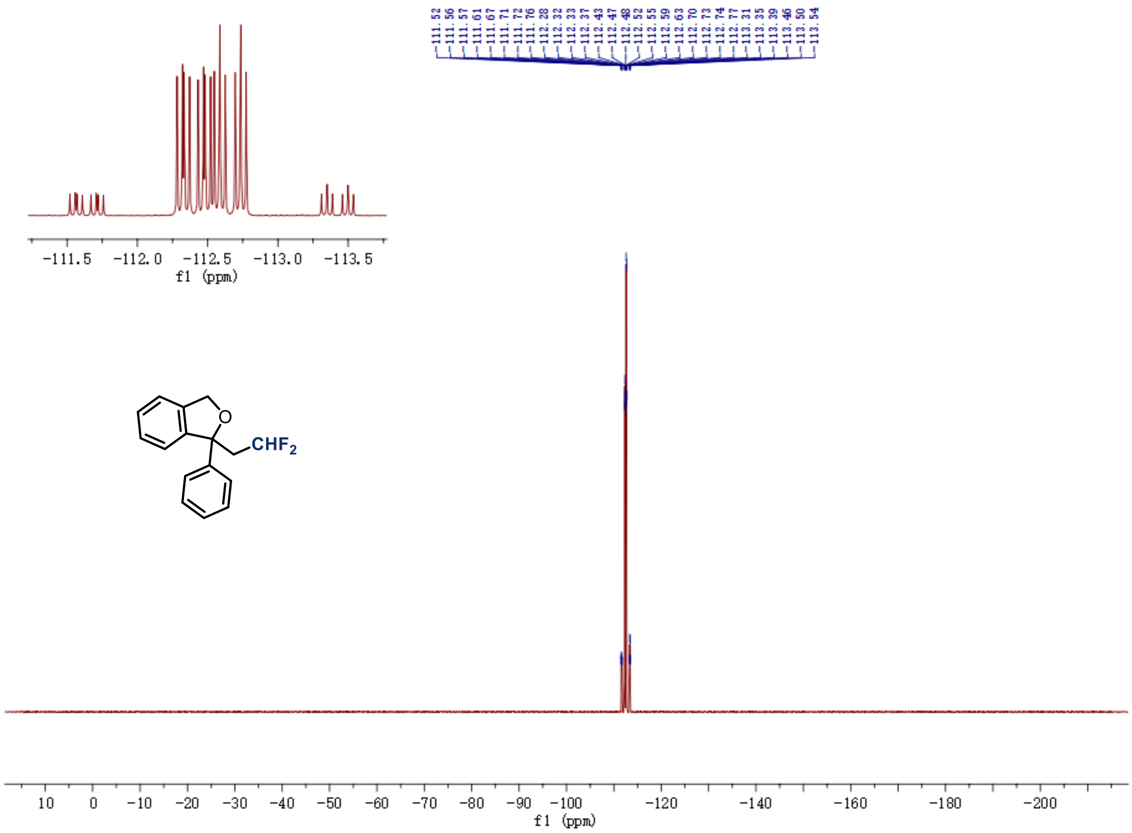

^{1}H-NMR of compound 14 (400 MHz, $CDCl_3$)

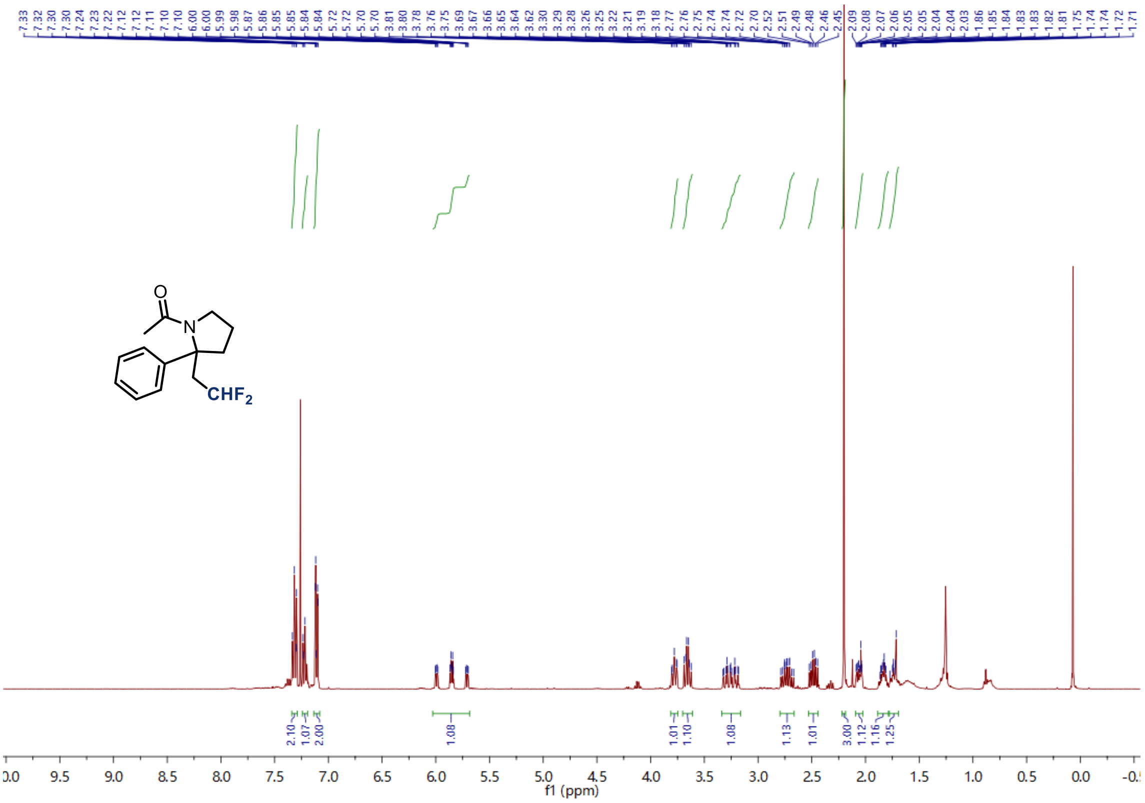

^{13}C-NMR of compound 14 (101 MHz, $CDCl_3$)

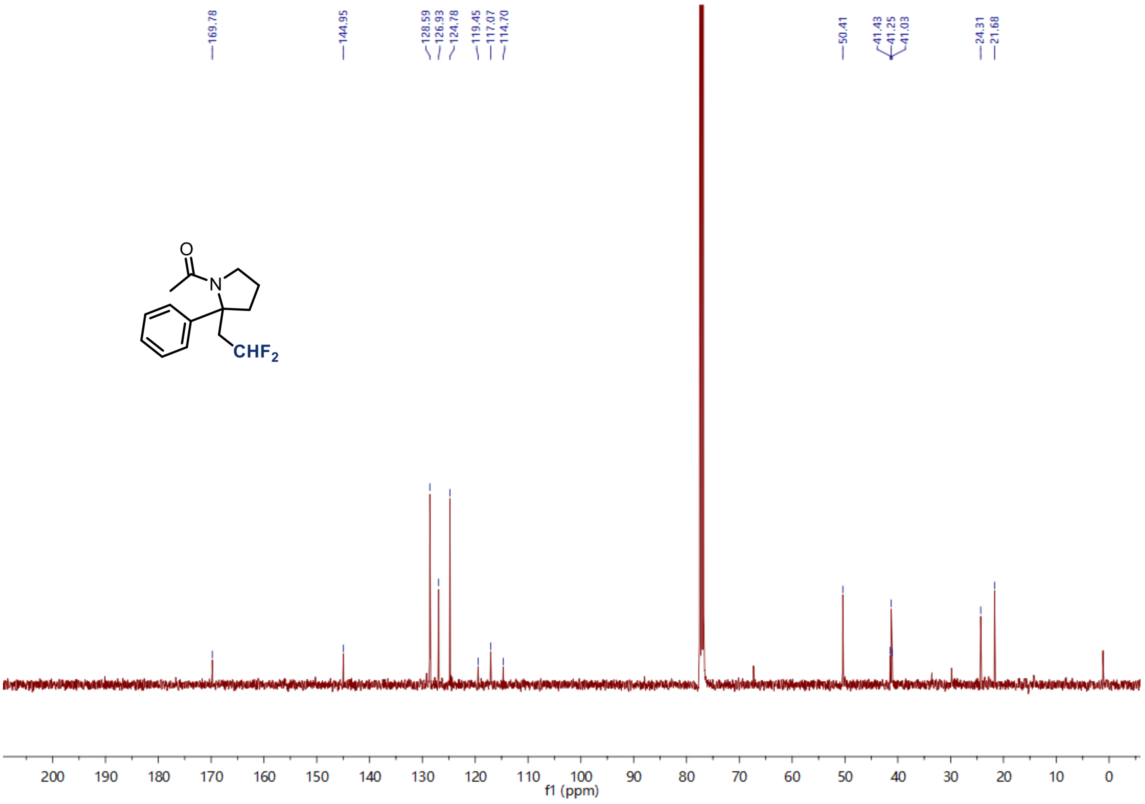

^{19}F-NMR of compound 14 (377 MHz, $CDCl_3$)

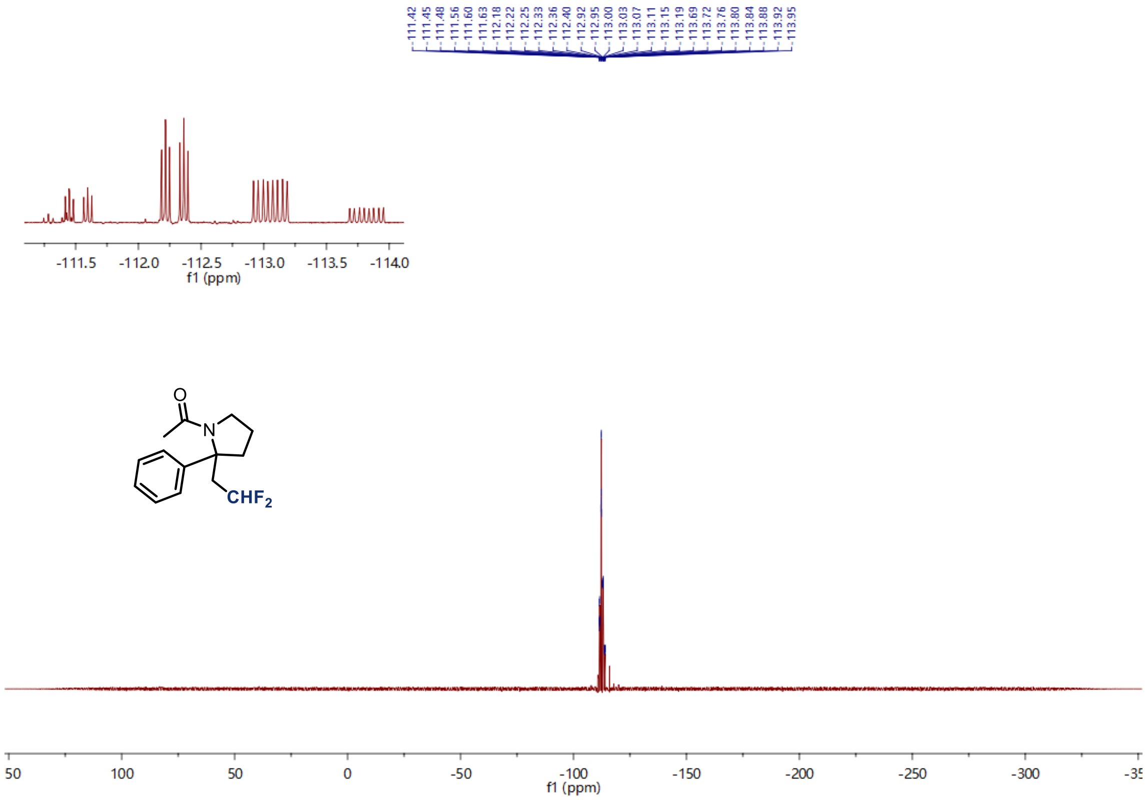

^{1}H-NMR of compound 17 (400 MHz, $CDCl_3$)

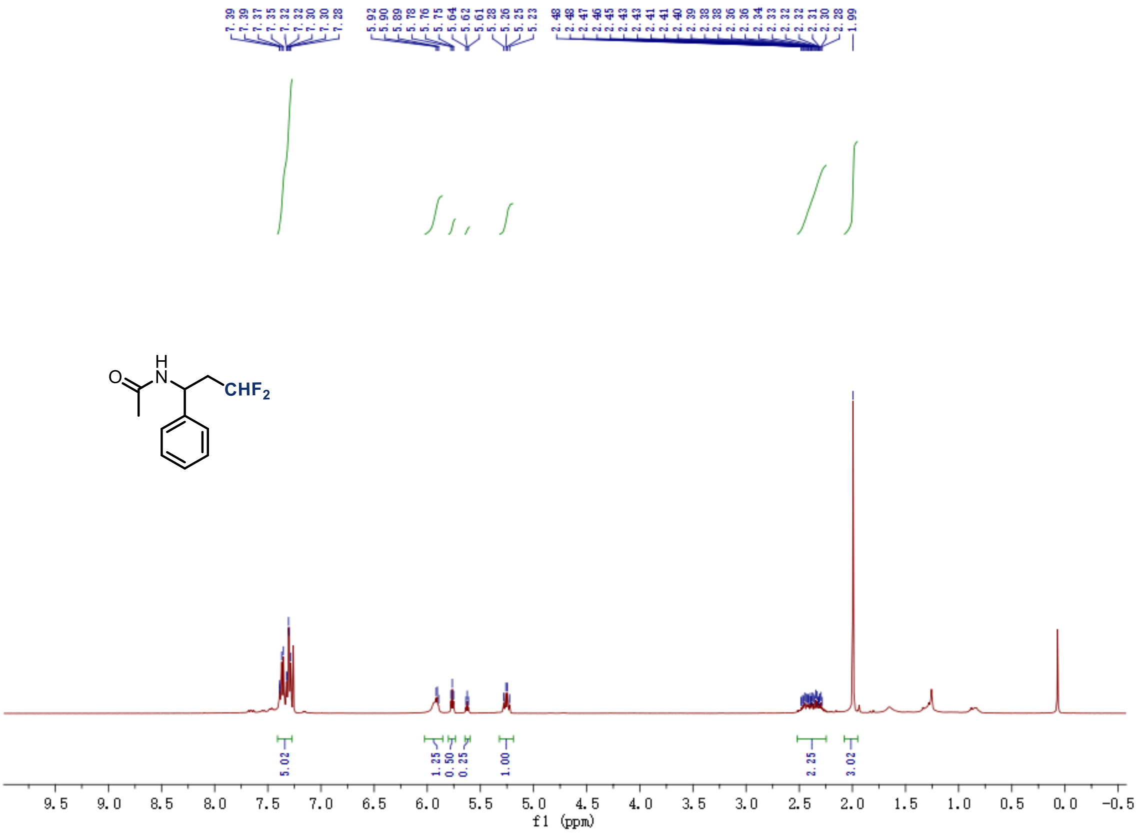

^{13}C-NMR of compound 17 (101 MHz, $CDCl_3$)

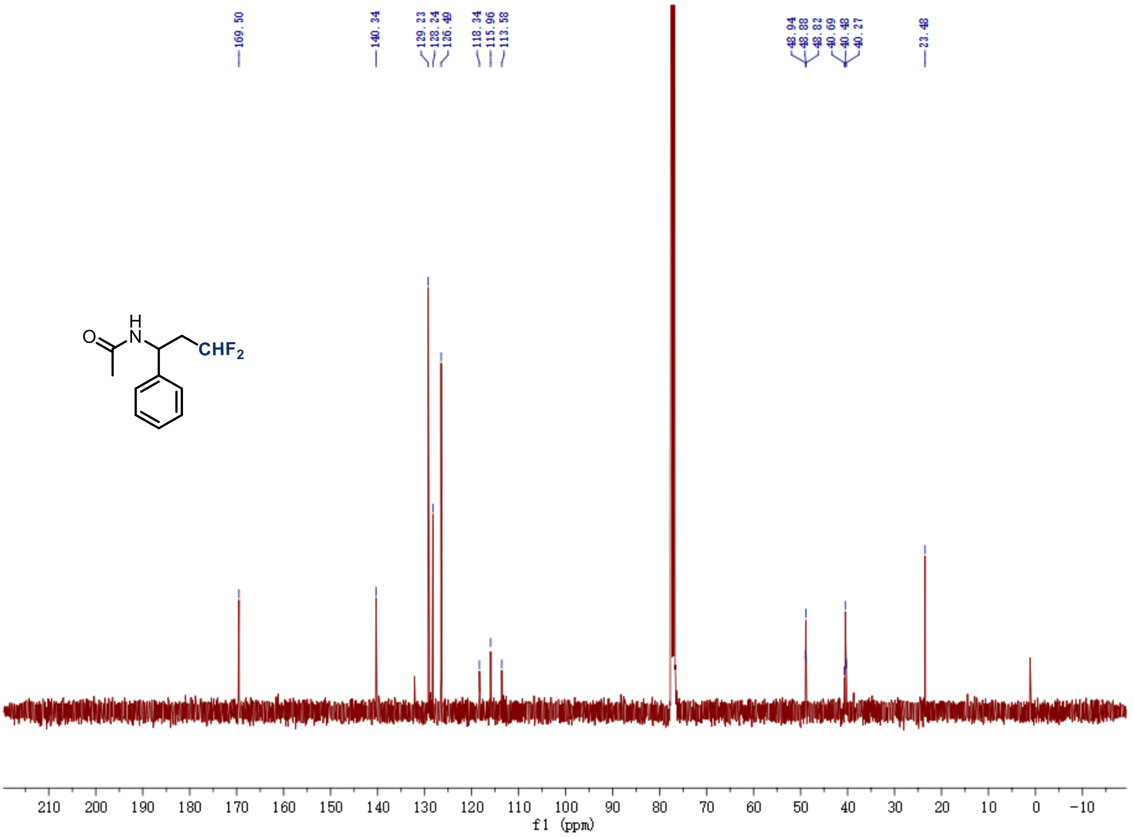

^{19}F-NMR of compound 17 (377 MHz, $CDCl_3$)

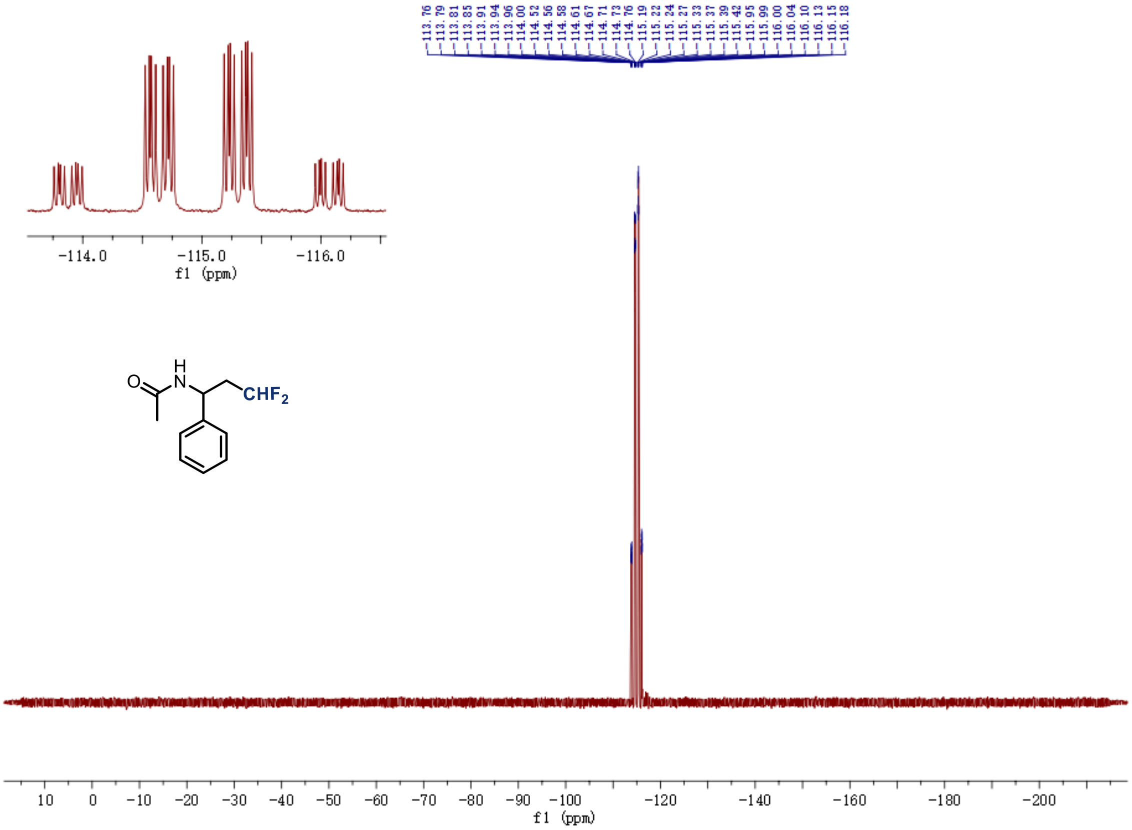

F. References

1 (a) Pegot, B.; Urban, C.; Bourne, A.; Le, T. N.; Bouvet, S.; Marrot, J.; Diter, P.; Magnier, E. *Eur. J. Org. Chem.* **2015**, 3069–3075. (b) Deng, Z.; Lin, J. H.; Cai, J.; Xiao, J. C. *Org. Lett.* **2016**, *18*, 3206–3209. (c) Zheng, J.; Cai, J.; Lin, J.-H.; Guo, Y.; Xiao, J.-C. *Chem. Commun*. **2013**, *49*, 7513–7515.

2 Noto, N.; Koike, T.; Akita, M. *Chem. Sci.* **2017**, *8*, 6375–6379.

3 Huang, H.; Li, X.; Yu, C.; Zhang, Y.; Mariano, P. S. *Angew. Chem. Int. Ed*, **2017**, *56*, 1500–1527.

4 Ahn, Y.; Jang, D. E.; Cha, Y.-B.; Kim, M.; Ahn, K.-H.; Kim, Y. C. *Bull. Korean. Chem. Soc.* **2013**, *34*, 107–111.

5 Alazet, S.; Le Vaillant, F.; Nicolai, S.; Courant, T.; Waser, J. *Chem. Eur. J.* **2017**, *23*, 9501–9504.

6 (a) Jesse L. P.; Scott E. D. *Org. Lett.* **2020**, *22*, 2501–2505. (b) Chen, Z.; Zhou, Q.; Chen, Q.-N.; Chen, P.; Xiong, B.-Q.; Liang, Y.; Tang, K.-W.; Xie, J.; Liu, Y. *Org. Biomol. Chem.* **2020**, *18*, 8677–8685.

7 Oh, C. H.; Jung, H. H.; Kim, K. S.; Kim, N, *Angew. Chem. Int. Ed* **2003**, *42*, 805–808.

8 Walker, P. R.; Campbell, C. D.; Suleman, A.; Carr, G.; Anderson, E. A, *Angew. Chem. Int. Ed.* **2013**, *52*, 9139–9143.

9 Wichmann, J.; Adam, G.; Roever, S.; Hennig, M.; Scalone, M.; Cesura, A. M.; Dautzenberg, F. M.; Jenck. F. *Eur. J. Med. Chem*. **2000**, *35*, 839–851.

10 Karila, D.; Leman, L.; Dodd, R. H. *Org. Lett.* **2011**, 13, 5830–5833.

11 Ang, N. W. J.; Oliveira, J. C. A.; Ackermann, L. *Angew. Chemie Int. Ed*. **2020**, 59 (31), 12842–12847.

12 Song, S.; Shou-Fei, Z.; Shuang, Y.; Shen, L.; Qi-Lin, Z. *Angew. Chem. Int. Ed.*, **2012**, *51*, 2708–2711.

13 Zeng, X.; Miao, C.; Wang, S.; Xia, C.; Sun, W. *Chem. Commun.* **2013**, 49, 2418–2420.

14 Brett N. H.; Andy W. C.; Qiu W. *J. Org. Chem*. **2019**, *84*, 1468-1488.

15 Fragale, G.; Wirth, T. *Eur. J. Org. Chem*. **1998**, 1361–1369.

16 Nakayama, Y.; Ando, G.; Abe, M.; Koike, T.; Akita, M. *ACS Catal*. **2019**, *9*, 6555–6563.

17 C. Adamo, V. Barone, "Toward reliable density functional methods without adjustable parameters: The PBE0 model," *J. Chem. Phys.*, **1999**, *110*, 6158-69. DOI: 10.1063/1.478522.

18 Gaussian 16, Revision B.01, Frisch, M. J.; Trucks, G. W.; Schlegel, H. B.; Scuseria, G. E.; Robb, M. A.; Cheeseman, J. R.; Scalmani, G.; Barone, V.; Petersson, G. A.; Nakatsuji, H.; Li, X.; Caricato, M.; Marenich, A. V.; Bloino, J.; Janesko, B. G.; Gomperts, R.; Mennucci, B.; Hratchian, H. P.; Ortiz, J. V.; Izmaylov, A. F.; Sonnenberg, J. L.; Williams-Young, D.; Ding, F.; Lipparini, F.; Egidi, F.; Goings, J.; Peng, B.; Petrone, A.; Henderson, T.; Ranasinghe, D.; Zakrzewski, V. G.; Gao, J.; Rega, N.; Zheng, G.; Liang, W.; Hada, M.; Ehara, M.; Toyota, K.; Fukuda, R.; Hasegawa, J.; Ishida, M.; Nakajima, T.; Honda, Y.; Kitao, O.; Nakai, H.; Vreven, T.; Throssell, K.; Montgomery, Jr. J. A.; Peralta, J. E.; Ogliaro, F.; Bearpark, M. J.; Heyd, J. J.; Brothers, E. N.; Kudin, K. N.; Staroverov, V. N.; Keith, T. A.; Kobayashi, R.; Normand, J.; Raghavachari, K.; Rendell, A. P.; Burant, J. C.; Iyengar, S. S.; Tomasi, J.; Cossi, M.; Millam, J. M.; Klene, M.; Adamo, C.; Cammi, R.; Ochterski, J. W.; Martin, R. L.; Morokuma, K.; Farkas, O.; Foresman, J. B.; Fox, D. J. Gaussian, Inc., Wallingford CT, 2016.

19 Grimme, S.; Antony, J.; Ehrlich, S.; Krieg, H. A consistent and accurate ab initio parameterization of density functional dispersion correction (DFT-D) for the 94 elements H-Pu. *J. Chem. Phys.*, **2010**, *132*, 154104.

20 Marenich, A. V.; Cramer, C. J.; Truhlar, D. G. Universal solvation model based on solute electron density and a continuum model of the solvent defined by the bulk dielectric constant and atomic surface tensions. *J. Phys. Chem. B*, **2009**, *113*, 6378-96.

21 (a) Hariharan, P. C.; Pople, J. A. Influence of polarization functions on molecular-orbital hydrogenation energies. *Theor. Chem. Acc.*, **1973**, *28*, 213-22. (b) Hehre, W. J.; Ditchfield, R.; Pople, J. A. Self-Consistent Molecular Orbital Methods. 12. Further extensions of Gaussian-type basis sets for use in molecular-orbital studies of organic-molecules *J. Chem. Phys.*, **1972**, *56*, 2257. (c) Ditchfield, R.; Hehre, W. J.; Pople, J. A. Self-consistent molecular orbital methods. 9. Extended Gaussian-type basis for molecular-orbital studies of organic molecules. *J. Chem. Phys.*, **1971**, *54*, 724.

22 https://doi.org/10.19061/iochem-bd-6-282.

23 Álvarez-Moreno, M.; de Graaf, C.; López, N.; Maseras, F.; Poblet, J. M.; Bo, C. Managing the computational chemistry big data problem: The ioChem-BD platform. *J. Chem. Inf. Model.* **2015**, *55*, 95–103.
